# Targeting mannosylation of nicastrin N-glycans attenuates **γ**-secretase activity and notch-dependent leukemia progression

**DOI:** 10.1172/JCI203202

**Published:** 2026-10-01

**Authors:** Hua Jiang, Weixiang Bian, Yanjun Cao, Zhuo Zhang, Yijia Chen, Yue Sui, Hongqiang Qin, Xu Li

**Affiliations:** 1 School of Life Sciences, Westlake University, Hangzhou, Zhejiang, China.; 2 Department of Urology, Institute of Urologic Science and Technology, The First Affiliated Hospital, School of Medicine, and; 3 Department of Radiation Oncology and the State Key Laboratory of Transvascular Implantation Devices, The Second Affiliated Hospital, School of Medicine, Zhejiang University, Hangzhou, Zhejiang, China.; 4 State Key Laboratory of Fine Chemicals, Department of Pharmaceutical Engineering, School of Chemical Engineering, Dalian University of Technology, Dalian, China.

**Keywords:** Cell biology, Oncology, Leukemias, Molecular biology, Signal transduction

## Abstract

γ-Secretase is a transmembrane protease complex that cleaves multiple type I transmembrane proteins, including amyloid precursor protein and neurogenic locus notch homolog protein (NOTCH). Although numerous γ-secretase inhibitors and modulators targeting Notch-dependent cancers have been developed in recent decades, their clinical translation has been hampered by low substrate specificity and on-target gut toxicity. Using a proteomics-based screening approach, we identified dedicator of cytokinesis protein 2 (DOCK2) as an interactor of the γ-secretase subunit nicastrin (NCSTN). We further demonstrate that DOCK2 regulates mannosylation of NCSTN N-glycans, which in turn modulates γ-secretase activity toward NOTCH receptors. Both genetic depletion of DOCK2 and pharmacological inhibition of NCSTN mannosylation with kifunensine attenuated Notch-dependent leukemia progression in vivo. Collectively, these findings uncover a regulatory mechanism underlying substrate-specific activation of γ-secretase and suggest a promising therapeutic strategy for Notch-related diseases.

## Abstract 2

Blocking DOCK2-regulated mannosylation of Nicastrin stops Notch-driven leukemia and avoids gut toxicity.

## Graphical abstract

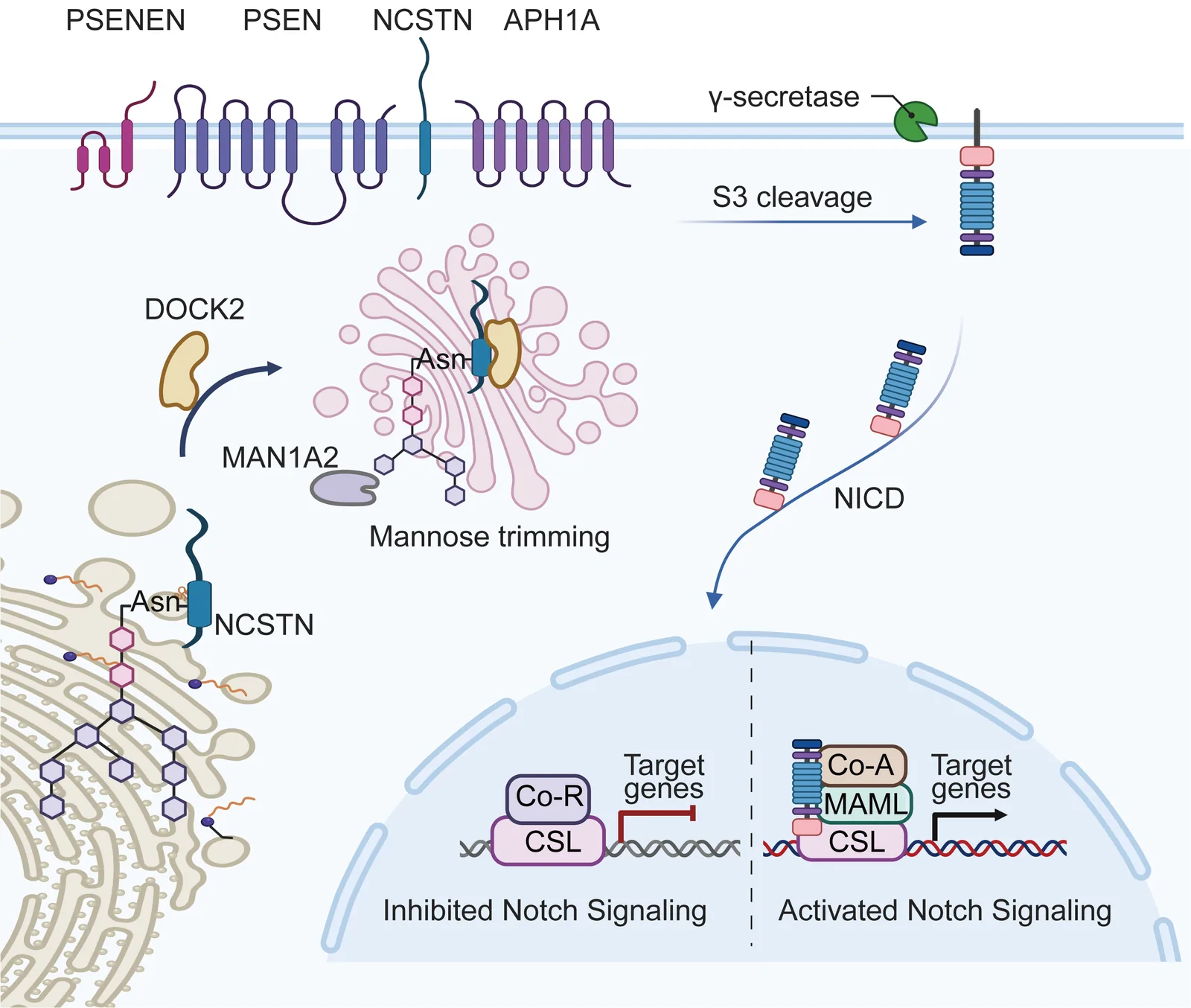

## Introduction

γ-Secretase is a large endoprotease complex composed of 4 core subunits: presenilin 1 (PSEN1) or PSEN2, nicastrin (NCSTN), anterior pharynx defective 1 (APH1), and presenilin enhancer 2 (PSENEN) (1). Among these, presenilin serves as the catalytic subunit of the enzyme complex. NCSTN recognizes over 40 ectodomain-shed substrates, including amyloid precursor protein (APP), neurogenic locus notch homolog protein (NOTCH), and receptor tyrosine-protein kinase erbB-4 (2, 3). APH1 serves as a scaffold to join PSEN and NCSTN (4), while PSENEN facilitates PSEN1/2 endoproteolysis, leading to the generation of cleaved PSEN-NTF and PSEN-CTF fragments (5). Despite the crucial role of the γ-secretase complex in regulating numerous essential cellular processes, its full protein interaction network remains uncharacterized. Further investigation is needed to elucidate the complex’s detailed composition, regulatory mechanisms, and substrate specificity.

γ-Secretase mediates the proteolytic cleavage of more than 140 substrates (6), including well-characterized substrates APP and NOTCH. γ-Secretase inhibitors (GSIs) have been proposed as therapeutic agents for Alzheimer’s disease (AD) and Notch signaling–driven cancers (7, 8). However, their clinical translation has been hindered by low selectivity and dose-limiting toxicities. A deeper understanding of the mechanism by which γ-secretase recognizes distinct substrates — such as APP and NOTCH — is therefore essential.

The Notch signaling pathway is an evolutionarily conserved pathway that plays critical roles in cell proliferation, differentiation, and fate decisions (9). In the canonical Notch pathway, ligand–receptor binding initiates sequential proteolytic cleavage by ADAM metalloprotease and γ-secretase, resulting in the release of the Notch intracellular domain (NICD). NICD then translocates to the nucleus, where it associates with the RBPJ transcription complex to activate target gene expression (10). Dysregulation of Notch signaling is implicated in a wide spectrum of human diseases, ranging from developmental disorders to complex conditions such as metabolic diseases and cancers. For instance, hyperactivation of NOTCH1 signaling has been established as an oncogenic driver in T cell acute lymphoblastic leukemia/lymphoma (T-ALL) (11, 12), chronic lymphocytic leukemia (13, 14), and breast cancer (15–17). Therapeutic approaches targeting the Notch pathway — including chemical and immunological strategies directed against NOTCH receptors, delta ligands, and γ-secretase — have demonstrated promising efficacy in preclinical studies (18–20). These therapeutic agents comprise GSIs, monoclonal antibodies targeting Notch ligands and receptors (18, 21), ADAM10/17 inhibitors (22), SERCA inhibitors (23), HSP90 inhibitors (24), and transcription complex inhibitors (25–27). However, clinical studies of these drugs in NOTCH-driven cancers have demonstrated limited efficacy, calling for the development of new GSIs with improved selectivity and safety profiles (28).

Among these agents, GSIs represent the most extensively studied class of small molecules targeting the Notch pathway. They act by blocking the proteolytic cleavage of NOTCH at the S3 site, thereby reducing intracellular levels of activated NOTCH (29–32). The US FDA recently approved the GSI nirogacestat for treating adults with desmoid tumors, rare soft tissue tumors associated with Notch pathway dysregulation (33). However, clinical trials have revealed limited efficacy of GSIs in other cancers, such as T-ALL, along with dose-limiting on-target and off-target toxicities. These include disruption of gastrointestinal epithelium and abnormalities in lymphoid tissues (30, 34), attributable to the essential role of Notch signaling in intestinal epithelial cell maintenance and the broad substrate specificity of γ-secretase, which influences multiple signaling pathways (35). Therefore, there is an urgent need for more selective therapeutic strategies targeting the γ-secretase/Notch axis.

To further understand the composition of the γ-secretase complex and its substrate recognition mechanisms, we constructed a γ-secretase interactome using tandem affinity purification combined with mass spectrometry (TAP-MS) and investigated potential regulators critical for its activity. We found that dedicator of cytokinesis 2 (DOCK2) physically and functionally interacted with NCSTN, affecting the N-glycosylation of NCSTN at the mannose trimming step and regulating γ-secretase activity to mediate Notch activation.

## Results

### Establishing a comprehensive γ-secretase protein–protein interaction network.

To comprehensively map the protein–protein interaction (PPI) landscape regulating γ-secretase activity, we generated HEK293T cell lines stably expressing each core component of human γ-secretase: PSEN1, PSEN2, APH1A, NCSTN, and PSENEN. We isolated the membrane, soluble, and chromatin fractions of these cell lines and combined these cellular fractions for TAP-MS to identify γ-secretase–associated protein complexes (Figure 1A). The TAP-MS analysis identified a number of prey proteins with diverse subcellular localizations (Supplemental Figure 1, A and B; supplemental material available online with this article; https://doi.org/10.1172/JCI203202DS1). High reproducibility was observed across biological replicates (Supplemental Figure 1, C–G). In total, we identified 144,028 peptides corresponding to 24,040 interacting proteins, which represented 5,395 unique prey proteins (Supplemental Figure 1A and Supplemental Table 1).

To distinguish bona fide γ-secretase–interacting proteins from nonspecific binders commonly encountered in affinity purification–MS studies (36), we employed the SAINT algorithm to assign confidence scores to each binary interaction (37). Using a SAINT score greater than 0.90 as the cutoff, we identified 1,139 high-confidence interactions among the 576 high-confidence interacting proteins (HCIPs) (Supplemental Figure 1A and Supplemental Table 2). Only 42 of these interactions had been previously reported (38), reflecting the limited characterization of the γ-secretase interactome to date. The majority represent unrecognized interactions, expanding the known protein landscape associated with this critical complex (Supplemental Figure 1H). Our dataset includes subunits of several complexes annotated in the CORUM database — such as the exocyst complex, mitofilin complex, COG complex, and ERAD-related proteins — as γ-secretase interactors (Supplemental Figure 1I).

To further evaluate the quality of this interaction network, we randomly selected 12 top-scoring PSEN1- and PSEN2-interacting proteins identified in this study and validated their interactions using co-IP assays (Supplemental Figure 1J). All 12 HCIPs confirmed strong affinity binding to their respective baits, supporting the reasonable quality of the interaction network (Supplemental Figure 1J). Taken together, these results suggest that our γ-secretase PPI network provides a reliable resource for future functional and mechanistic studies.

To obtain a comprehensive functional overview of the HCIPs and identify potential key regulators of specific γ-secretase activities, we performed Kyoto Encyclopedia of Genes and Genomes enrichment analysis (Supplemental Table 3) as well as cellular function and disease association analysis (Supplemental Table 4) using the HCIP dataset. As expected, the HCIPs were strongly enriched in established γ-secretase–associated biological processes, including endoplasmic reticulum protein processing, AD, and glycerophospholipid metabolism (Supplemental Figure 2, A and B). They were also significantly enriched in pathways related to embryonic and tissue development, hematopoiesis, immune response, and various cardiovascular, neurological, and metabolic disorders (Supplemental Figure 2, C–E) — all of which represent core physiological and pathological contexts in which γ-secretase is known to operate. Notably, NCSTN-interacting proteins were specifically enriched in glycosylation-related functions (Supplemental Figure 2F). The ALG family of mannosyltransferases and the MAN1 family of mannosidases were among the top-ranked interactors, suggesting that protein mannosylation of N-glycans may play an essential role in modulating γ-secretase function (Supplemental Figure 2G).

As each component of the γ-secretase complex carries out distinct functions in substrate recognition and processing, selectively inhibiting specific subunits could offer specific therapeutic strategies for γ-secretase–associated diseases (39). To this end, we conducted a detailed analysis of the interaction patterns among the 5 subunits, which revealed considerable diversity in both interaction profiles and disease associations across the HCIPs of individual subunits (Supplemental Figure 3, A and B). Consistent with the reported function and mutation of γ-secretase components, most components were enriched in leukemia and lipid metabolism (Supplemental Figure 3B). PSEN1/2 interactors were more enriched in neurological and cardiovascular diseases, whereas NCSTN interactors were associated with glycosylation-related disorders (40–43). These findings suggest that the HCIPs may regulate γ-secretase function in a subunit- and context-dependent manner.

To further enable targeted intervention, we investigated drug–target relationships and potential drug repurposing candidates using database mining (Supplemental Table 5). We constructed a network linking γ-secretase components, their interactors, and known targeted drugs (Supplemental Figure 3C), which may facilitate the development of subunit-specific therapies. For instance, gemcitabine could be repurposed for AD treatment (44), and telaglenastat (CB-839), a GLS inhibitor, has been shown to reduce Aβ deposition in APP/PS1 mice (45) and suppress Notch-driven breast cancer progression (46). Thus, our interaction network provides a rational foundation for repurposing existing drugs to treat γ-secretase–related diseases, including AD and Notch-driven cancers.

Finally, we integrated the HCIPs with additional annotations from functional databases to reconstruct a refined γ-secretase interaction network. This integrated network clusters HCIPs based on subcellular localization and biological function, offering a comprehensive resource for understanding the functional organization of the γ-secretase complex (Figure 1B).

### DOCK2 is a critical regulator of γ-secretase–mediated NOTCH processing.

γ-Secretase mediates the proteolytic processing of more than 140 substrates (6), among which 42 were identified in our unfiltered TAP-MS data, including well-characterized substrates such as APP, NOTCH, LRP1, and VLDLR (Supplemental Table 1), which attests to the high reliability of the dataset. To identify key regulators of γ-secretase activity in AD, we overlapped our HCIP dataset with proteins previously found to be significantly altered in AD patients (47, 48). This analysis revealed that over 200 AD-associated proteins interact with core components of the γ-secretase complex (Figure 2A), underscoring the reliability of our data and its potential utility in elucidating disease mechanisms and advancing therapeutic strategies for γ-secretase–related disorders.

γ-Secretase–mediated cleavage of NOTCH receptor is essential for Notch activation. Activating mutations in *NOTCH1* are associated with T-ALL (11), while loss-of-function mutations in Notch receptors have been identified in squamous cell carcinomas (SCCs) (49, 50). To explore the relationship between HCIPs and Notch-related cancers, we analyzed expression alterations and mutation profiles of these HCIPs across 4 leukemia and 3 SCC datasets (51, 52). Several HCIP-encoding genes, including *ADGRL2*, *DOCK2*, *ATM*, *ATR*, *DHCR7*, and *FANCA*, exhibited high alteration rates in both cancer types, suggesting their potential involvement in Notch pathway–driven tumorigenesis through modulation of γ-secretase activity on NOTCH processing (Figure 2, B and C). We further overlapped our HCIP dataset with known T-ALL driver genes from a patient cohort (53) and found that 9 of these drivers were enriched among our HCIPs (Figure 2, D and E). Among these, *MOGS*, *DOCK2*, *RNF213*, and *OBSCN* showed positive correlation with Notch signaling, as evidenced by elevated expression of Notch target genes (Figure 2, F–H). *DOCK2* emerged as a candidate driver of Notch-related cancers in both analyses. It is highly expressed in Notch-associated acute lymphoblastic leukemia (ALL) (Figure 2I), and its expression positively correlates with Notch downstream genes (*HES1*, *HEYL*, *HES2*, and *MYC*) in ALL patients (Figure 2, J and K, and Supplemental Figure 4, A and B). These findings position DOCK2 as a top candidate regulator and a promising therapeutic target in Notch-related malignancies.

As a core component of the γ-secretase complex, NCSTN is responsible for substrate recognition. To identify key interactors that specifically regulate γ-secretase activity toward NOTCH, we selected the top NCSTN-interacting proteins from our HCIP dataset and assessed their functional impact on γ-secretase–mediated cleavage of the NOTCH receptors using a Gal4VP16-based (GVP-based) reporter assay (Figure 3A). In this system, the NOTCH1 ΔE-GVP substrate contains a C-terminal GVP moiety and undergoes ligand-independent, constitutive cleavage by γ-secretase. Proteolytic processing at site S3 releases the intracellular domain, which translocates to the nucleus and activates luciferase expression under the control of the upstream activating sequence (UAS) promoter (Figure 3A). Using this GVP/UAS-Luc assay, we found that the selected interactors modulated γ-secretase activity to varying degrees, with DOCK2 exhibiting the most pronounced enhancement of NOTCH processing (Figure 3B). Consistent with the luciferase results, overexpression of these candidates also altered the levels of NICD1, especially DOCK2 and endoplasmic reticulum mannosyl-oligosaccharide 1,2-α-mannosidase (Figure 3C). Notably, DOCK2 increased NICD abundance in a dose-dependent manner and led to subsequent upregulation of Notch target genes (Figure 3, D and E).

To further validate DOCK2 as a bona fide NCSTN interactor, we performed reciprocal TAP-MS using cells expressing DOCK2 and established its interaction network (Supplemental Figure 4C). NCSTN was consistently identified as a top interactor of DOCK2. Functional analysis revealed that DOCK2-associated proteins participate in diverse processes, including focal adhesion, lipid metabolism, signal transduction, and proteolysis (Supplemental Figure 4D). Overlap between DOCK2 and NCSTN interactomes suggests that DOCK2 may specifically facilitate NCSTN function (Supplemental Figure 4, E and F).

We further validated the interaction between DOCK2 and NCSTN using epitope-tagged constructs, which confirmed a direct association (Figure 3, F and G). This interaction was corroborated by an in vitro pull-down assay with purified recombinant proteins (Figure 3H). Cross-linking MS via NCSTN pulldown also confirmed the existence of DOCK2 in the γ-secretase complex (Supplemental Figure 4G). To map the domain of DOCK2 responsible for binding NCSTN, we generated a series of DOCK2 deletion mutants (Figure 3I). The DHR2 domain (amino acids 1,196–1,620) was identified as essential for the interaction. Consistent with this, deletion of the DHR2 domain reduced both γ-secretase activity and the level of NICD1 compared with WT DOCK2 (Figure 3, J and K). Furthermore, DOCK2 overexpression enhanced the interaction between NCSTN and NOTCH1 ΔE, while DOCK2 KO weakened it (Figure 3, L and O). Correspondingly, NICD1 levels were reduced in DOCK2-KO cells (Figure 3M). KO of DOCK2 also decreased nuclear accumulation of active NOTCH1 ΔE, increased its membrane retention (Figure 3N), and impaired NCSTN–NOTCH1 ΔE binding (Figure 3O). A luciferase-based reporter assay confirmed that NOTCH1 ΔE-GVP processing was suppressed upon DOCK2 KO (Figure 3P). This defect was rescued by reexpression of either DOCK2 (Figure 3Q) or NICD1 (Figure 3R), indicating that γ-secretase activity depends on functional DOCK2. Together, these results demonstrate that DOCK2 positively regulates γ-secretase–mediated cleavage of NOTCH.

### Knocking out DOCK2 attenuates Notch activation–dependent leukemia cell invasion, migration, and tumorigenesis.

Aberrant activation of the Notch pathway is closely linked to the initiation and progression of T-ALL. Since DOCK2 plays a key role in γ-secretase–mediated NOTCH cleavage, we investigated its potential contribution to leukemogenesis. Consistent with its tissue expression profile, DOCK2 was highly expressed in all the T-ALL cell lines we tested (Figure 4A). For subsequent functional studies, we selected JURKAT and DND41 cell lines, both of which exhibit hyperactivated Notch signaling. Knocking out DOCK2 significantly decreased the level of NICD1 (Figure 4, B and C, and Supplemental Figure 4, H–K) and attenuated the expression of Notch target genes (Figure 4, D and E). DOCK2 has been reported to be a GEF that activates Rac1 and Rac2 (54). To test whether the enzymatic activity of DOCK2 regulates Notch signaling, we treated JURKAT cells with different doses of the DOCK2 inhibitor CPYPP. No prominent change in NICD1 level was observed (Supplemental Figure 5A). Overexpressing the DOCK2 enzymatically dead mutant produced a similar effect on NICD1 level as WT DOCK2 (Supplemental Figure 5B). These results indicate that DOCK2 is required for the activation of Notch signaling activation in T-ALL cells through a mechanism independent of its canonical GEF activity.

To further investigate the role of DOCK2 in leukemia pathogenesis, we generated DOCK2-KO JURKAT and DND41 cell lines and evaluated their phenotypic effects on proliferation, invasion, migration, and tumorigenesis. DOCK2 KO significantly impaired cell invasion (Supplemental Figure 5, C–F), migration (Figure 4, F and G, and Supplemental Figure 5, G and H), and anchorage-independent growth (Figure 4, H and I, and Supplemental Figure 5, I and J). Overexpression of NICD1 rescued these phenotypes resulting from DOCK2 KO (Supplemental Figure 5, K–N). In vivo, mice injected with DOCK2-KO cells exhibited markedly reduced tumor size and weight compared with controls (Figure 4, J–L). Tumors derived from DOCK2-KO cells showed decreased levels of NICD1 and downregulation of Notch target genes (Figure 4M and Supplemental Figure 5O), confirming that DOCK2 promotes tumorigenesis through Notch signaling.

To evaluate DOCK2’s contribution to leukemogenesis in vivo, we established luciferase-expressing JURKAT cells and injected them into immunodeficient NSG (NOD.Cg-Prkdcscid IL2rgtm1Wjl/SzJ) mice via the tail vein. Leukemia progression was monitored weekly using flow cytometry for T cell surface glycoprotein CD5 expression and bioluminescence imaging for luciferase activity (Figure 4N). DOCK2 KO significantly reduced leukemic burden and disease progression (Figure 4, O–R), mitigated splenomegaly (Figure 4, S and T), and attenuated infiltration of leukemia cells into the liver and spleen, as confirmed by H&E staining and CD5 immunohistochemistry (Figure 4, U and V). Together, these results demonstrate that DOCK2 is essential for Notch-dependent leukemia tumorigenesis in vivo.

### DOCK2 regulates NCSTN N-glycosylation and intracellular trafficking.

To elucidate the molecular mechanism by which the DOCK2–NCSTN interaction enhances γ-secretase activity and promotes Notch activation, we first investigated whether DOCK2 influences NCSTN protein level. Overexpression of DOCK2 increased NCSTN protein levels (Supplemental Figure 6A), while KO of DOCK2 decreased them (Figure 5A). To determine whether DOCK2 stabilizes NCSTN, we treated cells with cycloheximide to inhibit new protein synthesis and compared NCSTN degradation rates in WT versus DOCK2-KO cells (Supplemental Figure 6B). NCSTN was degraded faster in DOCK2-KO cells than in WT controls (Figure 5B), indicating that DOCK2 is required for NCSTN stability. Immunofluorescence staining showed that NCSTN and DOCK2 interact mostly in cell membrane and cytoplasm, with some interaction taking place in Golgi apparatus (Supplemental Figure 6C). Reduced membrane localization of NCSTN in DOCK2-KO cells was observed compared with WT cells (Figure 5C and Supplemental Figure 6, D and E). Consistent with this, DOCK2 KO decreased NCSTN levels at the membrane and increased its accumulation in the Golgi apparatus (Figure 5, D–F). A band shift of NCSTN was also observed (Figure 5D), suggesting potential alterations in its posttranslational modification or processing. Together, these results demonstrate that DOCK2 plays a critical role in regulating NCSTN processing, trafficking, and stability.

Our MS data suggest that NCSTN is extensively modified by N-glycosylation and palmitoylation, modifications known to cause significant protein band shifting. To determine how DOCK2 regulates NCSTN processing, we treated cells with inhibitors of palmitoylation and N-glycosylation. Inhibiting protein palmitoylation with 2-bromohexadecanoic acid (55) did not markedly affect the DOCK2 KO-induced band shift of NCSTN (Figure 5G). We then treated the cell lysates with endoglycosidase H (EndoH), which specifically cleaves oligomannose-type N-glycans added in the endoplasmic reticulum, or with peptide N-glycosidase F (PNGF), which cleaves both the oligomannose-type and the complex-type N-glycans added in the Golgi apparatus. This strategy enabled us to simultaneously determine whether the molecular weight increase was due to N-glycosylation and to assess the complexity of the glycan structures involved. Both EndoH and PNGF treatment induced a band shift in NCSTN. However, only PNGF abolished the difference between WT and DOCK2-KO cells (Figure 5H). This result suggests that DOCK2 primarily influences the complex-type N-glycosylation of NCSTN.

To characterize the alterations in NCSTN glycosylation, we immunoprecipitated NCSTN from WT, DOCK2-overexpressing, and DOCK2-KO JURKAT cells and performed lectin blotting to detect glycan modifications (Figure 5I). Changes in DOCK2 expression levels strongly correlated with changes in complex-type N-glycosylation on NCSTN, as probed with phytohemagglutinin-L. In contrast, mannose-rich glycans detected by concanavalin A remained unaffected by DOCK2 modulation (Figure 5I). These findings indicate that loss of DOCK2 primarily impairs the complex-type N-glycosylation and structural maturation of NCSTN, thereby preventing its trafficking from the Golgi apparatus to the plasma membrane.

### DOCK2 controls NCSTN mannose trimming to enable Notch recognition by γ-secretase.

We predicted potential N-glycosylation sites on NCSTN using NetNGlycan and experimentally validated them by glycoproteomic MS, identifying 10 modified sites from 16 predicted N-glycan sites in NCSTN (Supplemental Table 6). To determine which site(s) mediates DOCK2-dependent glycosylation and γ-secretase activation, we generated a series of NCSTN mutants with asparagine-to-glutamine substitutions at each glycosite and assessed their electrophoretic mobility in the presence or absence of DOCK2. Mutation of N45 to glutamine (N45Q) resulted in an obvious reduction in molecular weight (Figure 5J), indicating that DOCK2 regulates NCSTN N45 glycosylation. To evaluate whether N45 glycosylation functionally influences NCSTN and γ-secretase activity, we established NCSTN-KO cells (Figure 5K). Loss of NCSTN suppressed γ-secretase–mediated processing of NOTCH1 ΔE-GVP. Reexpression of WT NCSTN rescued this phenotype, whereas the N45Q mutant only had a partial effect (Figure 5L). Consistently, in DND41 cells, NCSTN KO impaired leukemic cell invasion and anchorage-independent growth. These phenotypes were rescued by WT NCSTN but not by the N45Q mutant (Supplemental Figure 6, F–J). Further glycomic MS analysis revealed that DOCK2 KO significantly reduced the abundance of glycans with N-acetylneuraminic acid moieties (Figure 5M and Supplemental Figure 6K).

Since the N-acetylneuraminic acid moiety is incorporated during the sialylation step of N-glycosylation, we investigated whether DOCK2 influences NCSTN sialylation. We treated WT and DOCK2-KO JURKAT cells with sialylation and fucosylation inhibitors. However, no prominent change was observed (Supplemental Figure 6L), suggesting that DOCK2 functions upstream of the sialylation step. We next compared the effects of N-glycosylation inhibitors kifunensine (56, 57), an α-mannosidase I inhibitor, and NB-DNJ, an α-glucosidase inhibitor, on NCSTN electrophoretic mobility. Kifunensine but not NB-DNJ treatment abolished the difference in NCSTN migration between WT and DOCK2-KO cells (Figure 5N) and blocked DOCK2-induced NCSTN modifications (Supplemental Figure 6M), indicating that mannose trimming is essential for DOCK2-mediated glycosylation of NCSTN. Consistent with this, kifunensine also suppressed DOCK2-enhanced γ-secretase activity in the NOTCH1 ΔE-GVP processing assay (Supplemental Figure 6N). Furthermore, we observed that DOCK2 depletion specifically reduced the interaction between NCSTN and MAN1A2, but not other glycosidases (Supplemental Figure 6O). RNA-seq analysis showed that there is an overlap of downregulated genes between DOCK2 KO, Dibenzazepine, a potent γ-secretase inhibitor (DBZ), and kifunensine treatment compared with WT cells (Supplemental Figure 7, A–D). Further enrichment analysis showed they were associated with hematopoietic cell lineage and the Notch signaling pathway (Supplemental Figure 7E), indicating DOCK2 and kifunensine function in Notch signaling regulation. Although Notch receptor activation depends on complex N-glycosylation during its maturation, DOCK2 KO or kifunensine treatment did not markedly affect precursor NOTCH1 levels or other γ-secretase components (Supplemental Figure 7, F and G), indicating that DOCK2 modulates Notch signaling primarily through NCSTN. We further detected NCSTN ubiquitination upon DOCK2 KO as glycosylation usually crosstalk with ubiquitination for modulating protein stability. Increased ubiquitination intensity of NCSTN in DOCK2-KO cells was observed under MG132 pretreatment (Figure 5O). Together, these results demonstrate that DOCK2 regulates the MAN1A2-mediated mannose trimming step of NCSTN N-glycosylation, which in turn governs γ-secretase activity toward NOTCH substrates (Figure 5P).

### Therapeutic targeting of DOCK2-regulated NCSTN mannosylation inhibits leukemogenesis in vivo.

GSIs inevitably induce severe gut toxicity as a result of complete inhibition of NOTCH signaling in intestinal cells. Analysis of single-cell data from the Human Protein Atlas (HPA) revealed that DOCK2 is highly expressed in the hematological system — particularly in immune cells — but is rarely detected in intestinal tissues (Figure 6, A and B). Consistent with this, tissue staining confirmed strong DOCK2 expression in the spleen, while no detectable expression was observed in the intestine (Figure 6, C and D). These findings suggest that targeting DOCK2 could mitigate the dose-limiting, on-target toxicities associated with conventional GSIs.

Kifunensine, a potent and selective α-mannosidase I inhibitor, has demonstrated favorable safety profiles in preclinical studies (56–58). Given its ability to effectively inhibit DOCK2-mediated NCSTN N-glycosylation and subsequent γ-secretase–dependent NOTCH activation, we evaluated its therapeutic potential in Notch-driven leukemia. Kifunensine effectively suppressed T-ALL cell growth with an IC_50_ of approximately 20 μM (Figure 6E). It also abolished DOCK2-enhanced colony formation (Figure 6, F and G) and cellular invasion (Figure 6, H and I). A hERG (human Ether-à-go-go-Related Gene) assay showed that kifunensine exhibited an IC_50_ exceeding 100 μM (Figure 6J and Supplemental Table 7), suggesting an acceptable cardiac safety margin. Pharmacokinetic analysis after tail vein injection revealed a *t_1/2_* of 12.5 h (Supplemental Figure 7H and Supplemental Table 7), and its stability in human liver microsomes was indicated by a *t_1/2_* greater than 184 minutes (Supplemental Table 7). Notably, due to the low expression of DOCK2 in intestinal tissue, high-dose kifunensine treatment did not affect intestinal goblet cells (Figure 6K), suggesting the absence of on-target intestinal toxicity typically associated with conventional Notch pathway inhibitors. These results support kifunensine as a promising therapeutic candidate for Notch-related cancers with a reduced risk of on-target adverse effects.

We assessed the therapeutic efficacy of kifunensine in both cell line–derived (Figure 7, A–E) and patient-derived xenograft leukemia mouse models (Figure 7, F–J). Bioluminescence imaging and histological analysis showed that kifunensine treatment significantly reduced leukemic infiltration and alleviated associated pathological damage (Figure 7, A–E). In patient-derived xenograft leukemia models, kifunensine effectively suppressed tumor progression, as indicated by a decreased proportion of CD5^+^ cells in peripheral blood (Figure 7, G and H), improved tissue morphology in H&E-stained sections (Figure 7I), and extended host survival (Figure 7J).We also confirmed the safety of kifunensine in gastrointestinal tissue and blood-brain barrier penetration (Figure 7, K–M). Only minimal histopathological changes were observed in the stomach. No abnormalities were observed in other gastrointestinal organs, and body weight remained normal (Figure 7, K and L). Gross examination revealed no remarkable findings. Brain penetration was evaluated by measuring drug concentrations in plasma and brain tissues at multiple time points following dosing (Figure 7M). The unbound brain/plasma ratio was approximately 0.025, indicating very limited brain penetration.

Collectively, our results demonstrate that targeting DOCK2 or DOCK2-mediated NCSTN N-glycosylation represents a promising therapeutic strategy for Notch-dependent leukemia, with the potential to minimize on-target side effects.

## Discussion

Elucidating the control of NOTCH processing is particularly important for selectively targeting γ-secretase in Notch-driven cancers, while avoiding the adverse effects, such as those observed in AD therapeutic attempts, that arise from nonspecific inhibition of Notch signaling.

Here, we established a PPI network for the core subunits of γ-secretase and identified many previously unrecognized interactors that critically modulate γ-secretase activity. Among these, DOCK2, an interacting partner of NCSTN, showed a strong functional correlation with γ-secretase–mediated NOTCH1 processing. DOCK2 is a GEF that explicitly activates the small GTPase Rac (54) and was reported to promote tumor progression across several cancer types (59–63), many of which involve Notch signaling (63–65). Our findings reveal that DOCK2 specifically regulates γ-secretase–dependent cleavage of NOTCH receptors and is essential for Notch signaling activation in T-ALL cells, suggesting that beyond its established immunomodulatory roles, DOCK2 may represent a potential therapeutic target in leukemia.

During its maturation and incorporation into the γ-secretase complex, NCSTN undergoes complex N-glycosylation in the endoplasmic reticulum and Golgi apparatus — a process essential for its function within the active enzyme (66–68). Our study demonstrates that DOCK2 primarily regulates the mannosidase-mediated mannose trimming step of N-glycosylation of NCSTN. Both genetic ablation of DOCK2 and pharmacological inhibition of NCSTN glycosylation with kifunensine attenuated Notch-dependent leukemia progression, highlighting the therapeutic potential of targeting the DOCK2/NCSTN axis in Notch-activated leukemia. Furthermore, the favorable biosafety and pharmacokinetic profiles of kifunensine support its promise for future clinical translation.

This study, while establishing DOCK2 as a key regulator of mannosylation of NCSTN N-glycans and Notch signaling in T-ALL, has several limitations. First, due to the pleiotropic nature of both DOCK2 and protein N-glycosylation, we cannot fully exclude the possibility that some of the observed antileukemic effects integrate Notch-independent mechanisms. Although our in vitro rescue experiments support a central role for Notch signaling, definitive quantification of its specific contribution in vivo would require future studies employing strategies such as NICD1 reconstitution in animal models. Second, at the mechanistic level, further investigation is needed to elucidate the precise means by which DOCK2 recruits or modulates the MAN1 family of mannosidases and to obtain direct structural-functional insights into how glycosylation fine-tunes NCSTN conformation and substrate selectivity. Third, from a translational perspective, while the preliminary biosafety and pharmacokinetic profile of kifunensine is promising, comprehensive long-term toxicity and immunogenicity studies are needed before clinical application can be considered. Finally, the development of agents that directly and specifically disrupt the DOCK2–NCSTN PPI would provide more definitive causal evidence and potentially enhance therapeutic specificity compared with broader glycosylation inhibitors. Taken together, while the DOCK2/NCSTN/mannosylation axis represents a novel and potent vulnerability in T-ALL, addressing these mechanistic, pharmacological, and translational gaps is crucial for its future development as a therapeutic strategy.

## Methods

Detailed specifications for all constructs, compounds, cell lines, and procedures are provided in Supplemental Methods.

### Sex as a biological variable.

Our study examined male and female mice, and no discernible differences between sexes were identified.

### Statistics.

The data were analyzed via Prism 7.0 software (GraphPad) and are presented as the mean ± SEM. Unpaired 2-tailed Student’s *t* tests were used to determine statistically significant differences between 2 groups. Multiple-group comparisons were performed via 1-way ANOVA. *P* values < 0.05 were considered significant.

### Study approval.

All the animal experiments were performed in accordance with a protocol approved by the Institutional Animal Care and Use Committee of Westlake University (AP#20-023-LX).

### Data availability.

All the data needed to evaluate the conclusions of the paper are presented in the paper and supplemental materials. Values for all data points in graphs are reported in the Supporting Data Values file. The MS proteomics data have been deposited in the ProteomeXchange Consortium (http://proteomecentral.proteomexchange.org) via the PRIDE partner repository with the dataset identifier PXD057036. The glycoproteomic data have been deposited to the ProteomeXchange Consortium via the iProX partner repository (69, 70) with the dataset identifier PXD065491. RNA-seq data has been deposited in the Gene Expression Omnibus (GSE320527).

## Author contributions

XL and HQ were responsible for conceptualization, funding acquisition, resource provision, and supervision of the study. HJ and WB developed methodology. HJ, WB, Y Cao, ZZ, Y Chen, and YS performed experimental investigations. HJ, WB, and XL wrote the manuscript, and all authors contributed to review and editing of the manuscript. Authorship order among co–first authors was determined based on the relative magnitude of their contributions to the study.

## Conflict of interest

XL is a cofounder and scientific consultant for Groovy Medicine.

## Funding support

- Key Regional Research and Development Program, China Ministry of Science and Technology (2023CSJZN0600 to XL).
- Natural Science Foundation of China (32270764 and 91954103 to XL, 32400615 to HJ, and 32300591 to WB).
- Natural Science Foundation of Zhejiang Province (LR23C070001 to XL).

## Supplementary Material

Supplemental data

Unedited blot and gel images

Supplemental table 1

Supplemental table 2

Supplemental table 3

Supplemental table 4

Supplemental table 5

Supplemental table 6

Supplemental table 7

Supporting data values

## Figures and Tables

**Figure 1:**
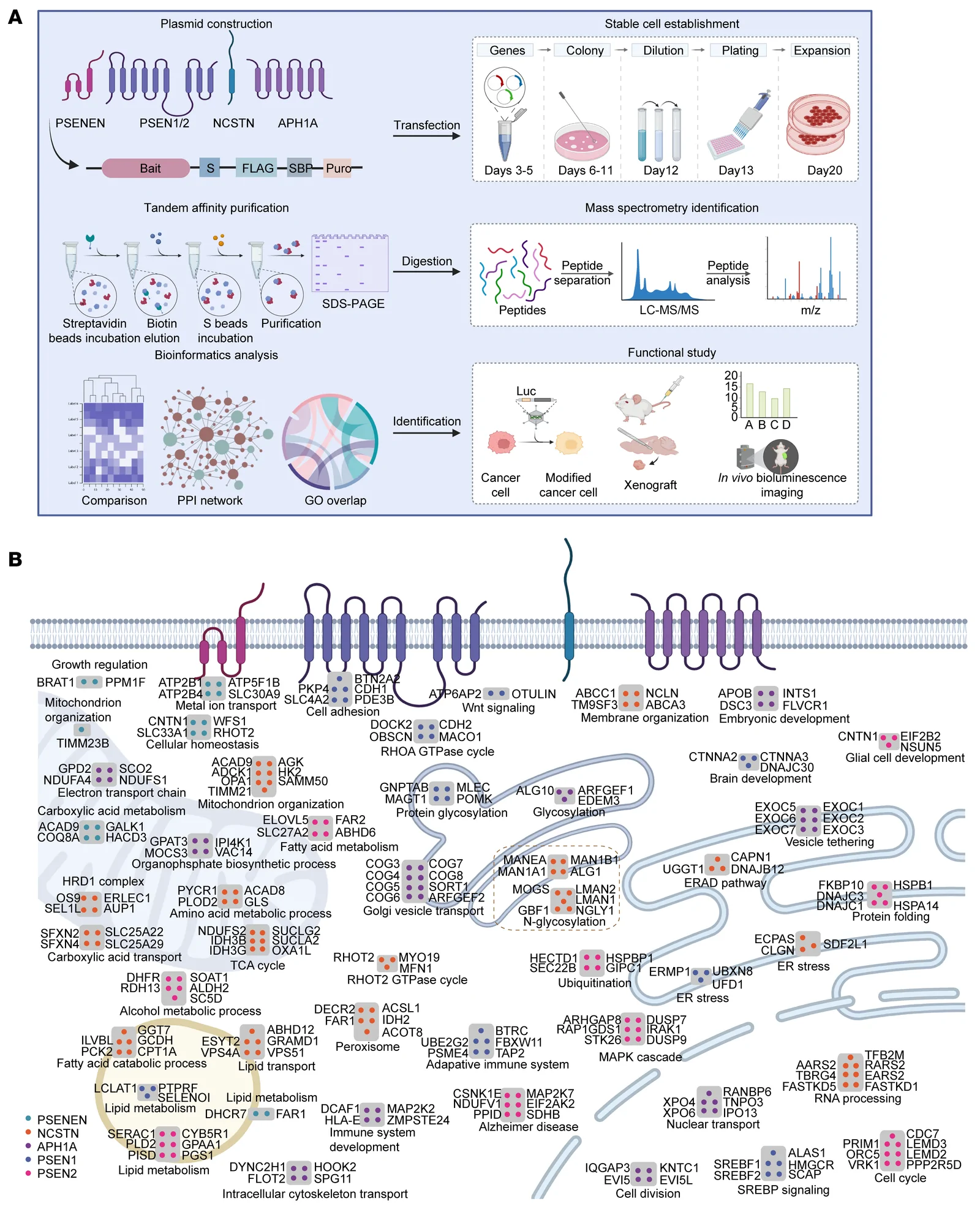
Establishing a comprehensive γ-secretase PPI network. (**A**) Schematic overview of the integrated proteomic and computational workflow used to construct the human γ-secretase PPI network in HEK293T cells. LC-MS/MS, liquid chromatography–tandem mass spectrometry. Created in BioRender. (**B**) Integrated interaction landscape of the human γ-secretase complex. HCIPs were categorized according to cellular function and subcellular localization based on Gene Ontology analysis and literature curation. Colored nodes represent HCIPs identified for each bait protein.

**Figure 2:**
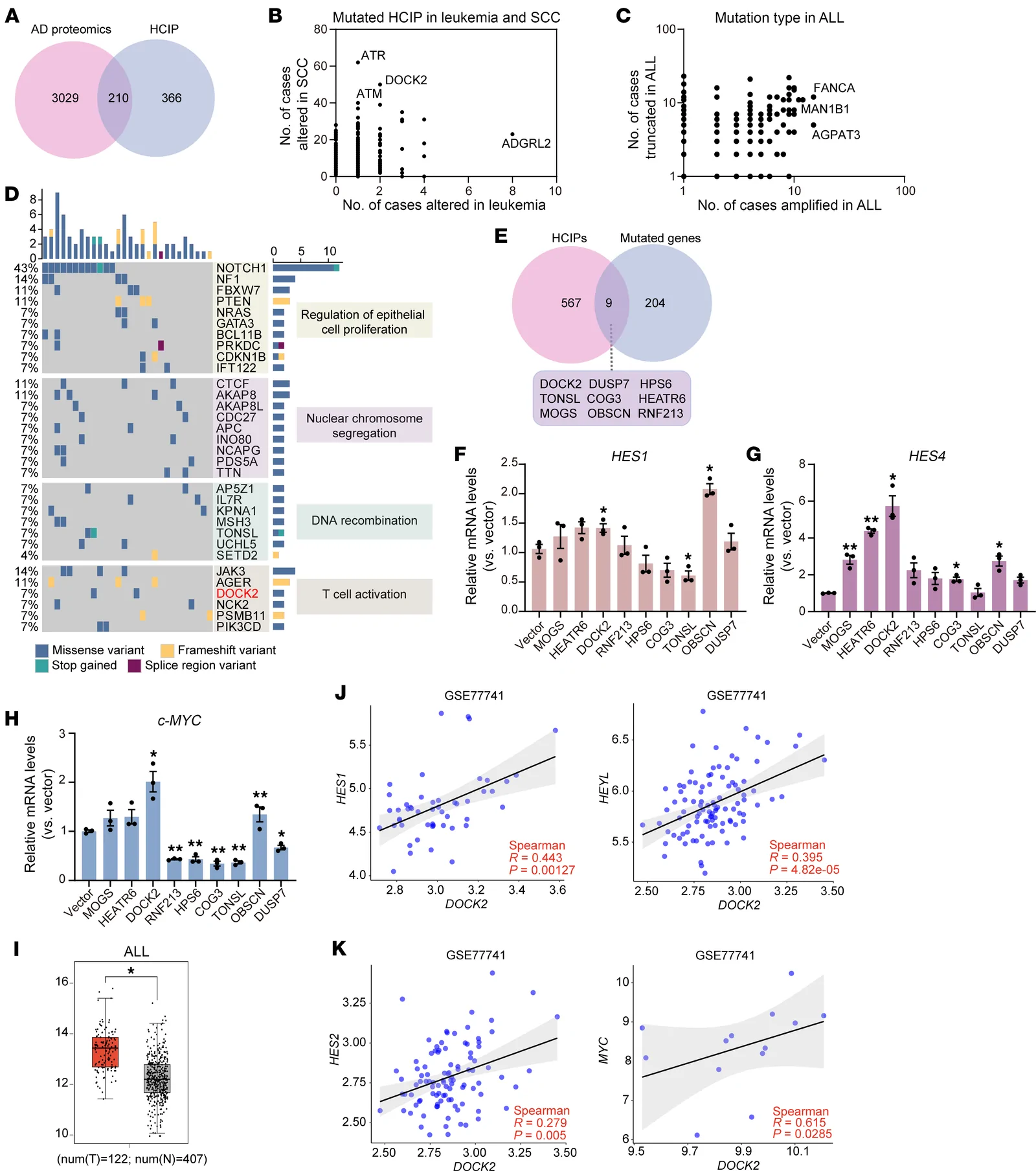
Identification of DOCK2 as a critical regulator of γ-secretase–mediated NOTCH processing. (**A**) Venn diagram showing the overlap between the HCIP set from this study and proteins dysregulated in patients with AD. (**B**) Alteration profiles of individual HCIPs in leukemia versus SCC datasets. (**C**) Frequency of truncations/deep deletions versus amplifications of HCIPs in ALL datasets. (**D**) Top mutated HCIP-encoding genes identified by whole-exome sequencing in 130 patients with T-ALL. (**E**) Venn diagram showing overlap between HCIPs and frequently mutated genes in T-ALL, highlighting 9 shared genes. (**F**–**H**) Effects of modulating the 9 overlapping genes on Notch target gene expression, as measured by RT-qPCR in JURKAT cells. (**I**) DOCK2 expression levels in Notch-associated cancers based on TNMplot. The box-and-whisker plots depict the minimum and maximum values (whiskers), the upper and lower quartiles, and the median. (**J** and **K**) Correlation between *DOCK2* and *HES1*, *HEYL*, *HES2*, and *MYC* expression in patients with T-ALL. Only samples with complete data for the respective gene pair were included in each correlation analysis. Mean ± SEM; **F**–**H**, *n* = 3; *P < 0.05, **P < 0.01, 1-way ANOVA; **I**, *n* as indicated.

**Figure 3:**
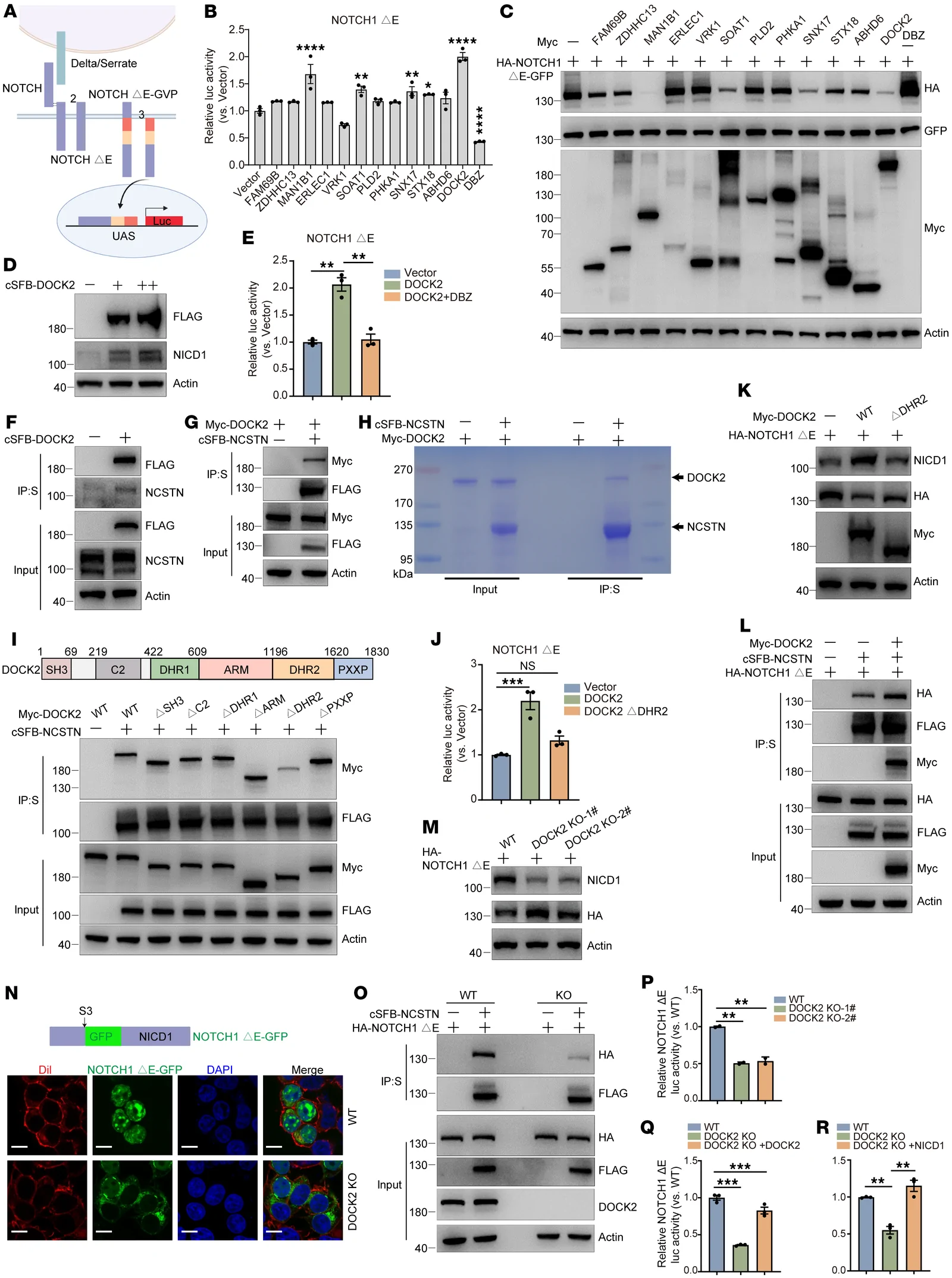
DOCK2 directly interacts with NCSTN and promotes γ-secretase–mediated NOTCH processing. (**A**) Schematic of ligand-induced Notch receptor processing and the γ-secretase reporter assay. (**B**) HEK293T cells were transfected, and the cleavage of NOTCH1 ΔE-GVP was analyzed. DBZ: 10 μM, 12 h, as positive control. (**C**) Evaluation of NOTCH1 ΔE processing in HEK293T cells expressing HA-NOTCH1 ΔE-GFP. (**D**) DOCK2-overexpressing HEK293T cells were subjected to Western blotting against NICD1. (**E**) γ-Secretase activity assay in HEK293T cells expressing DOCK2 with or without DBZ treatment. (**F** and **G**) HEK293T cells were transfected as indicated and subjected to co-IP using S beads. (**H**) In vitro pull-down assay using purified cSFB-NCSTN and Myc-DOCK2 incubated with S beads, followed by SDS-PAGE and Coomassie blue staining. (**I**) Interaction between cSFB-NCSTN and Myc-tagged WT or truncated DOCK2 mutants was assessed in HEK293T cells. (**J**) γ-Secretase activity assay evaluating NOTCH1 ΔE processing in HEK293T cells expressing DOCK2 ΔDHR2. (**K**) Western blot analysis of NICD1 in HEK293T cells expressing the DOCK2 ΔDHR2 mutant. (**L**) HEK293T cells were cotransfected as indicated, subjected to co-IP assay with S beads, and probed using FLAG, Myc, HA, and actin. (**M**) WT or DOCK2-KO HEK293T cells were subjected to Western blotting. (**N**) WT or DOCK2-KO HEK293T cells were transfected with GFP-NOTCH1 ΔE and subjected to immunofluorescence detection for nuclear translocation. Actin-tracker Dil was used for membrane labeling, and DAPI was used for nuclear staining. Scale bars: 10 μm. (**O**) WT or DOCK2-KO HEK293T cells were transfected and subjected to a co-IP assay, followed by Western blotting. (**P**–**R**) NOTCH1 ΔE processing was evaluated in WT or DOCK2-KO cells treated as indicated. Mean ± SEM; **B**–**H** and **J**–**R**, *n* = 3; *P < 0.05, **P < 0.01, ***P < 0.001, ****P < 0.0001, 1-way ANOVA.

**Figure 4:**
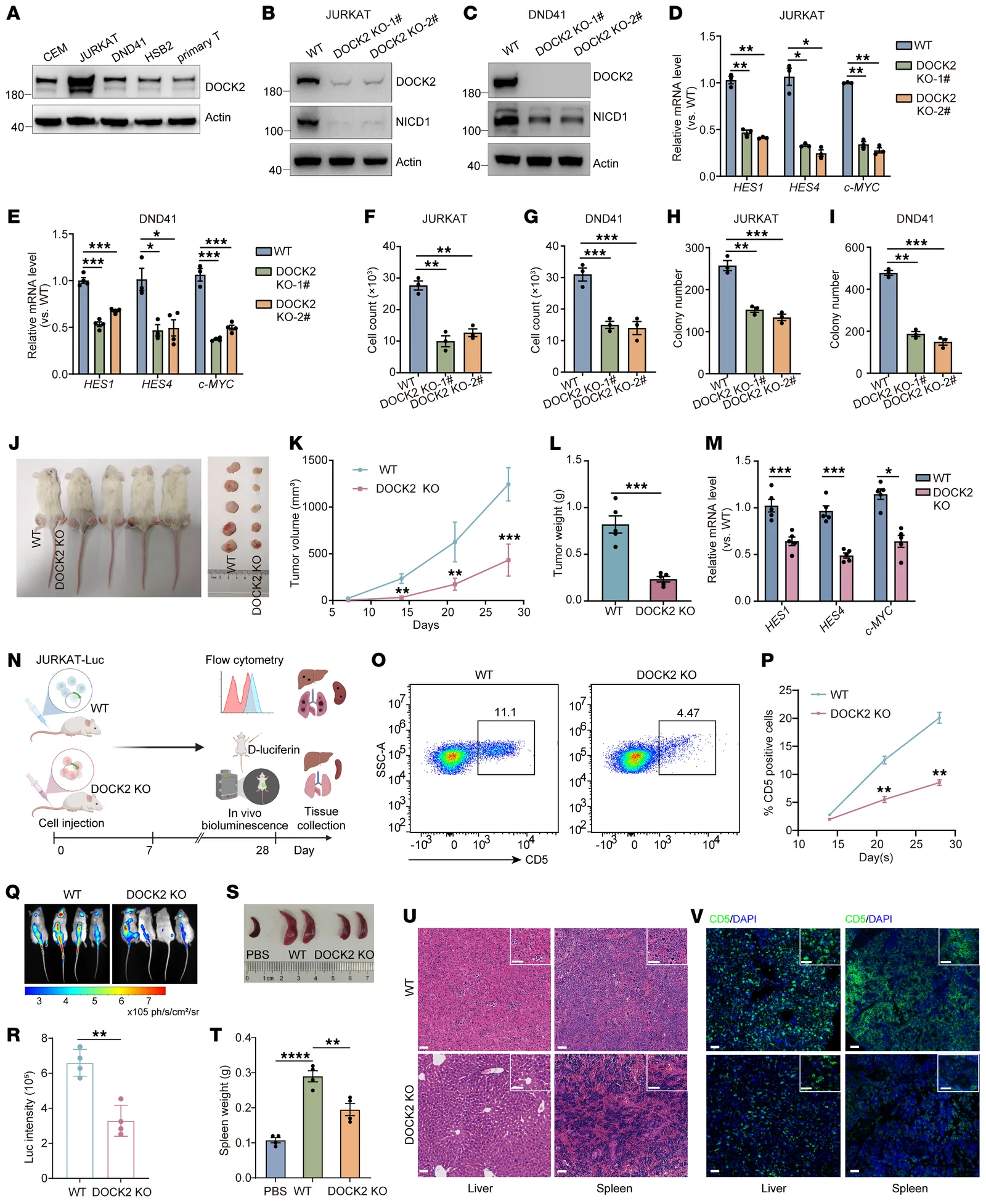
DOCK2 KO attenuates Notch signaling–dependent leukemia invasion, migration, and tumorigenesis. (**A**) Western blot analysis of DOCK2 and actin expression across multiple leukemia cell lines. (**B** and **C**) CRISPR/Cas9-mediated KO of DOCK2 in JURKAT (**B**) and DND41 (**C**) cells was confirmed by Western blotting. (**D** and **E**) mRNA levels of Notch target gene expression in DOCK2-KO JURKAT (**D**) and DND41 (**E**) cells were determined by RT-qPCR. (**F** and **G**) The migration abilities of WT and DOCK2-KO JURKAT (**F**) and DND41 (**G**) cells were assessed via a Transwell migration assay. Migrated cells in the lower chamber were quantified. (**H** and **I**) Anchorage-independent tumorigenesis of WT and DOCK2-KO JURKAT (**H**) and DND41 (**I**) cells was measured via a soft agar colony formation assay. The number of colonies was counted. (**J**–**L**) Xenograft tumor growth assays were performed using WT or DOCK2-KO JURKAT cells. Mice were euthanized 4 weeks after injection; tumors were excised, photographed, and analyzed. Tumor volume (**K**) and weight (**L**) were measured. (**M**) qPCR analysis of Notch target genes in tumor tissues. (**N**) NSG mice received tail vein injections of luciferase-expressing WT or DOCK2-KO JURKAT cells. Leukemia progression was monitored via flow cytometry and bioluminescence imaging. (**O** and **P**) Percentage of CD5^+^ leukemia cells in peripheral blood, assessed weekly by flow cytometry (**O**) and summarized (**P**). (**Q**) Representative bioluminescence image illustrating in vivo leukemia infiltration. (**R**) Quantitative analysis of luciferase intensity in **Q**. (**S**) Representative spleen images from each experimental group. (**T**) Spleen weight from each experimental group. (**U** and **V**) Leukemic infiltration in liver and spleen tissues evaluated by H&E staining (**U**) and CD5 immunofluorescence (**V**). Scale bars: 50 μm. Mean ± SEM; **A**–**I**, *n* = 3; **J**–**M**, *n* = 5; **O**–**T**, *n* = 4; *P < 0.05, **P < 0.01, ***P < 0.001, ****P < 0.0001, 1-way ANOVA.

**Figure 5:**
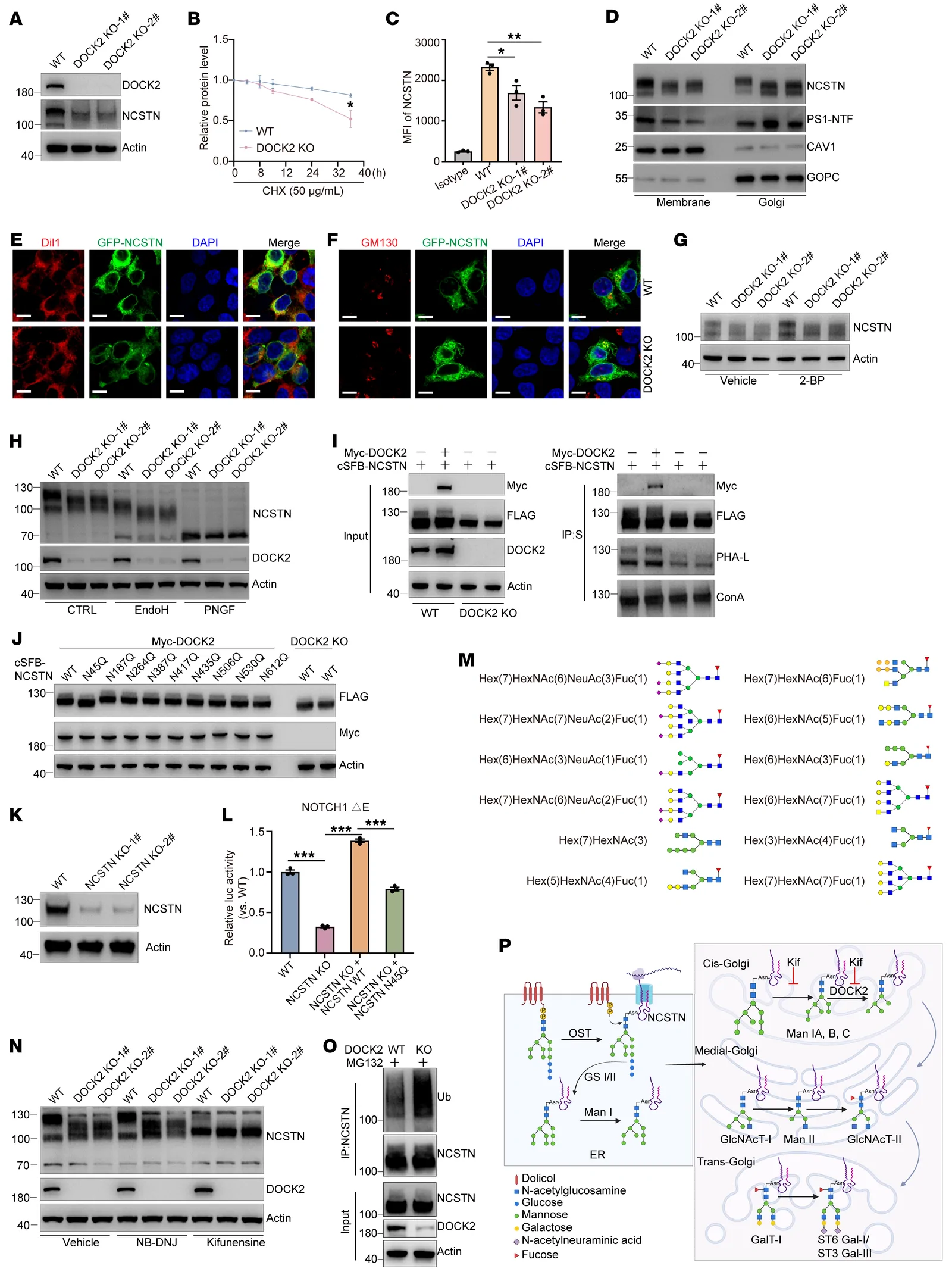
DOCK2 regulates NCSTN intracellular trafficking via N-glycosylation. (**A**) NCSTN protein level in DOCK2-KO HEK293T was evaluated. (**B**) WT and DOCK2-KO HEK293T cells were treated with 50 μg/mL cycloheximide (CHX) for the indicated times. Protein stability was evaluated and quantified. (**C**) Quantification of membrane-localized NCSTN mean fluorescence intensity in intact WT and DOCK2-KO JURKAT cells. (**D**) Subcellular fractionation of WT and DOCK2-KO JURKAT cells. Lysates were immunoblotted. (**E** and **F**) Immunofluorescence of WT and DOCK2-KO HEK293T cells transfected with GFP-NCSTN. Scale bars: 10 μm. (**G**) WT and DOCK2-KO JURKAT cells were treated with 2-bromohexadecanoic acid (2-BP) (50 μM, 12 h) and subjected to Western blotting. (**H**) Cell lysates from WT and DOCK2-KO JURKAT cells were treated with the glycosylases EndoH or PNGF and subjected to Western blotting. (**I**) Lectin blots of NCSTN immunoprecipitated from HEK293T cells. (**J**) HEK293T cells were cotransfected with Myc-DOCK2 and cSFB-tagged NCSTN or its glycosite mutants. The cell lysates were analyzed by Western blotting. (**K**) Validation of NCSTN-KO HEK293T cells generated via CRISPR/Cas9. (**L**) WT or NCSTN-KO HEK293T cells transfected with WT-NCSTN or N45Q mutant were subjected to a GVP-UAS–based γ-secretase assay. (**M**) Structural profiling of N-linked glycans at the NCSTN N45 site by MS. (**N**) WT and DOCK2-KO JURKAT cells were treated with *N*-butyl-deoxynojirimycin (NB-DNJ; 1 mM) or kifunensine (10 μM) for 24 h. The cell lysates were immunoblotted with NCSTN. (**O**) WT and DOCK2-KO JURKAT cells were pretreated with MG132 (10 μM) for 6 h, then the cell lysates were subjected to co-IP analysis against NCSTN and immunoblotted with ubiquitin (Ub). (**P**) Schematic illustrating regulation of NCSTN N-glycosylation by DOCK2 and inhibition by kifunensine. Created in BioRender. Mean ± SEM; **A**–**L** and **N**, *n* = 3; *P < 0.05, **P < 0.01, ***P < 0.001, 1-way ANOVA.

**Figure 6:**
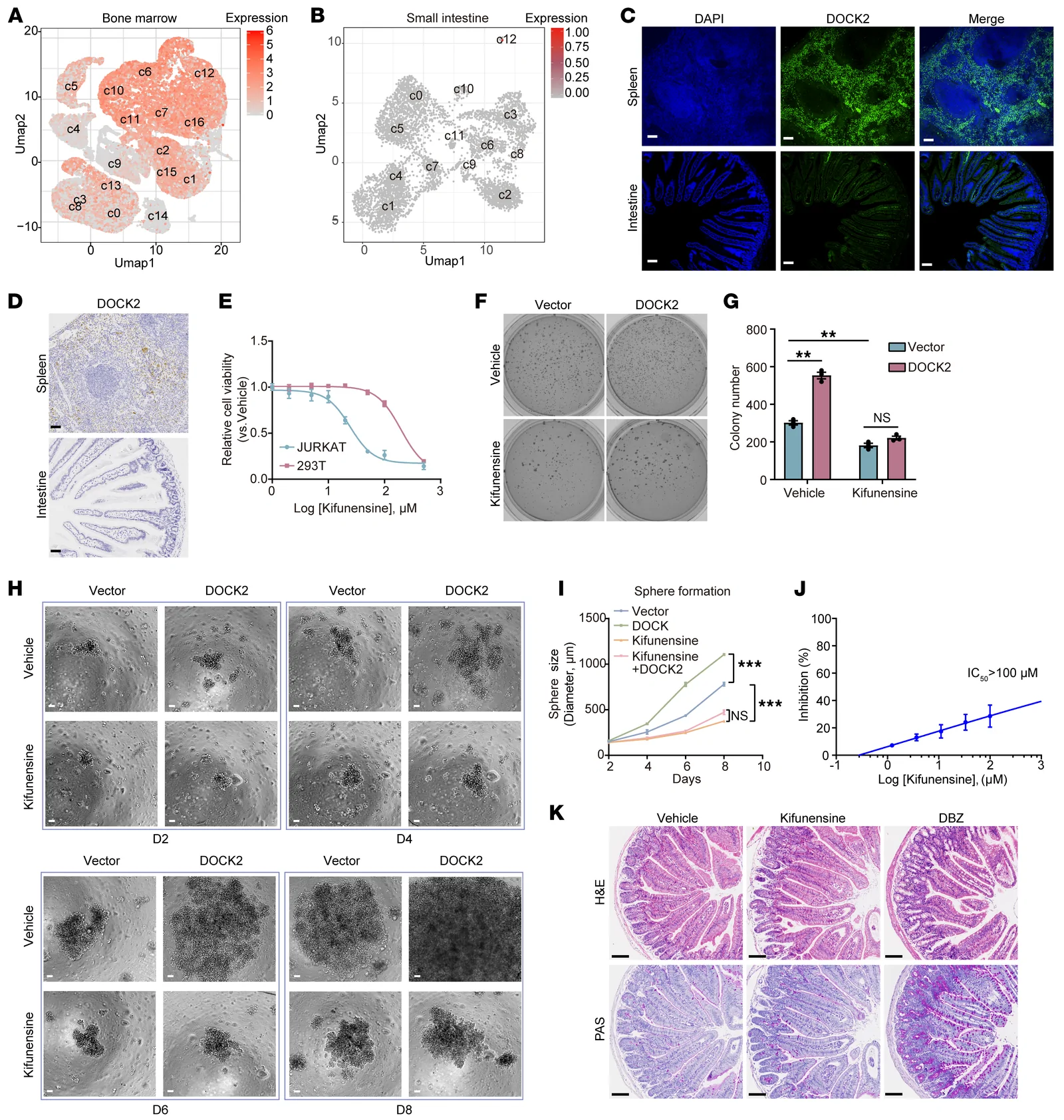
Inhibition of DOCK2-mediated NCSTN N-glycosylation by the α-mannosidase inhibitor kifunensine. (**A**) scRNA-seq analysis of DOCK2 expression in human bone marrow (HPA database). Cell clusters: c0/3/8/14 (T cell); c1/15 (monocyte); c2 (hematopoietic precursor cell); c4 (plasma cell).c5 (B cell); C6/7/10/11/12/16 (neutrophil); c9 (erythroid progenitor cell); c13 (NK cell). (**B**) scRNA-seq analysis of DOCK2 expression in human small intestine (HPA database). Cell clusters: c0/1/4/7 (proximal enterocyte); c2/8 (goblet cell); c3/6/9 (undifferentiated cell); c10 (Paneth cell); c11 (enteroendocrine cell); c12 (mixed immune cell). (**C**) Immunofluorescence staining of DOCK2 in mouse spleen and intestine tissues. Scale bars: 50 μm. (**D**) Immunohistochemistry staining of DOCK2 in mouse spleen and intestine tissues. Scale bars: 50 μm. (**E**) Cell viability of JURKAT and HEK293T cells treated with kifunensine was evaluated by a CCK-8 assay. (**F**–**I**) WT and DOCK2-overexpressing JURKAT cells were treated with vehicle or 10 μM kifunensine and then subjected to colony formation (**F** and **G**) and sphere formation (**H** and **I**) assays. Scale bars: 50 μm. (**J**) The hERG channel inhibition assay for kifunensine. (**K**) H&E and periodic acid–Schiff (PAS) staining of intestinal goblet cells in mice treated with vehicle, 10 mg/kg kifunensine, or 5 mg/kg GSI DBZ for 3 weeks. Scale bars: 100 μm. Mean ± SEM; **C**–**L**, *n* = 3; **P < 0.01, ***P < 0.001, 1-way ANOVA.

**Figure 7:**
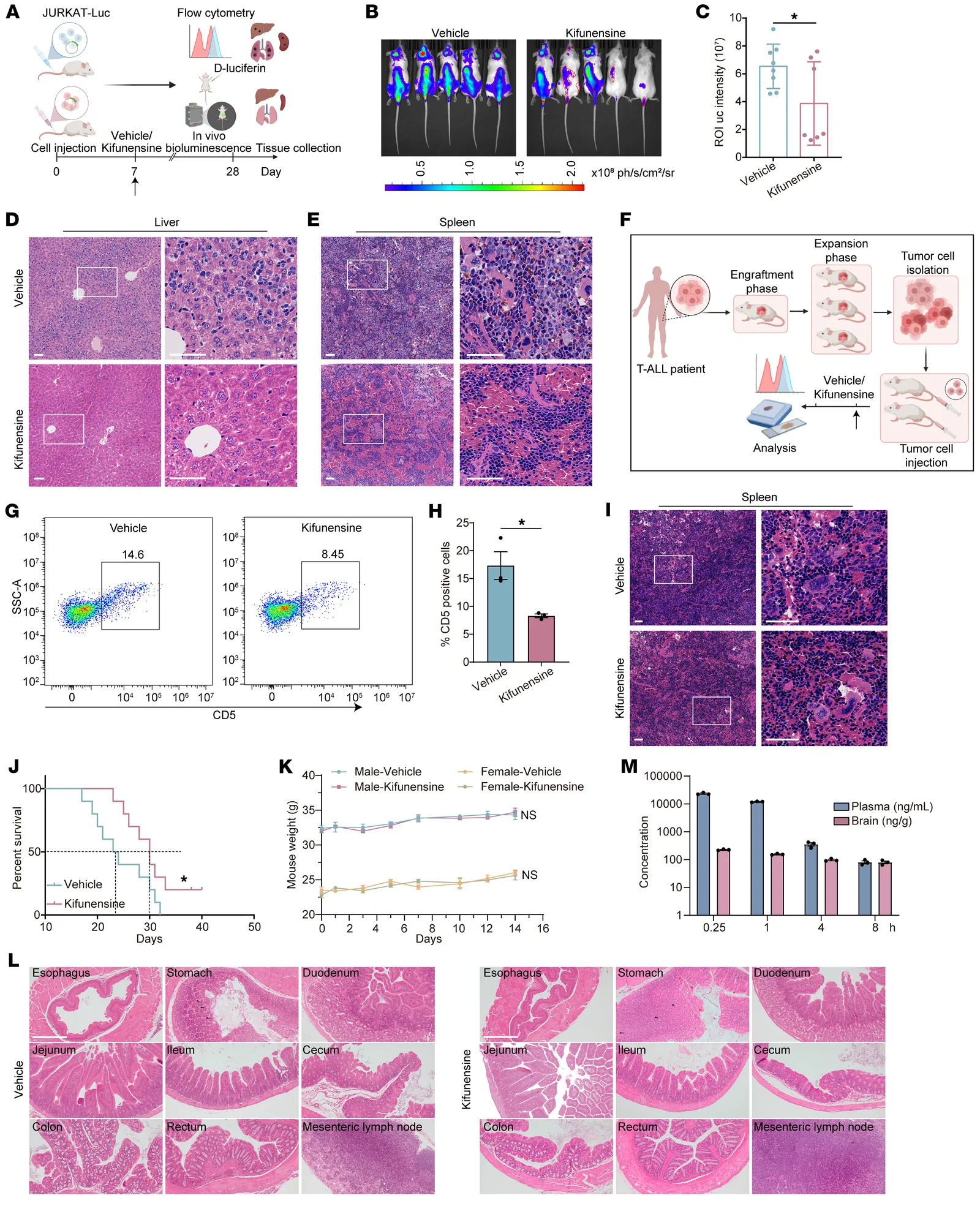
Inhibition of N-glycosylation with kifunensine attenuates Notch-dependent leukemia in vivo. (**A**) NSG mice received tail vein injections of luciferase-expressing JURKAT cells, followed by a 3-week treatment with 10 mg/kg kifunensine or vehicle. Leukemia progression was monitored by bioluminescence imaging. (**B**) Representative bioluminescence image showing leukemia infiltration in vivo. *n* = 5. (**C**) Quantitative analysis of the luciferase intensity presented in **B**. (**D** and **E**) Tumor cell infiltration in liver and spleen tissues from each group was evaluated via H&E staining. Scale bars: 50 μm. (**F**) Schematic of the patient-derived xenograft model establishment and treatment workflow. Patient-derived leukemia cells were subcutaneously engrafted in NSG mice, expanded, then transplanted intravenously into secondary NSG recipients to establish a disseminated leukemia model for kifunensine efficacy testing. (**G** and **H**) Flow cytometry analysis of CD5^+^ leukemia cells in peripheral blood. Representative dot plots are shown. (**I**) H&E staining of spleen sections demonstrating extent of leukemic infiltration across treatment groups. Scale bars: 50 μm. (**J**) Survival curves of PDX model mice from **F** following kifunensine or vehicle treatment. (**K**) Mouse weight after kifunensine administration. (**L**) H&E staining of gastrointestinal tissues. Scale bars: 50 μm. (**M**) Plasma and brain concentration of kifunensine. Mean ± SEM; **B**–**E**, *n* = 5; **G**–**I**, *n* = 3; J, *n* = 10, **K** and **L**, *n* = 6, 3 male and 3 female; **M**, *n* = 3; *P < 0.05, unpaired 2-tailed Student’s *t* test.

## Version 1 10/01/2026

Electronic publication
