# Supplementary material for: Targeting mannosylation of nicastrin N-glycans attenuates γ-secretase activity and notch-dependent leukemia progression
Source: J Clin Invest. 2026 Oct 1;136(19):e203202. doi: 10.1172/JCI203202 (PMC13626847; doi:10.1172/JCI203202)
Supplement: Supplementary file 1 [file jci-136-203202-s062.pdf]

Supplemental Methods

Constructs. Genes encoding all the components of the γ-secretase complex and candidates were amplified from cDNA via PCR, cloned and inserted into a pDONR201 vector (Invitrogen, CA) as entry clones, and transferred to Gateway-compatible SFB- or MYC-tagged destination vectors for protein expression. Deletion mutants of DOCK2 and point mutations of NCSTN were generated via a site-directed mutagenesis kit (C112-02, Vazyme) and verified by sequencing.

Compounds. Lith-O-Asp analog (10 μM, GC31977, Glpbio), P-3FAX-Neu5Ac (50 μM, GC50255, Glpbio) were used as sialylation inhibitors and SGN-2FF (100 μM, GC33031, Glpbio) was used as fucosylation inhibitor. 2-BP (50 μM, GC19850, Glpbio) was used as a palmitoylation inhibitor. NB-DNJ (1 mM, GC12631, Glpbio) was used to inhibit the ER α-glucosidases I and II. Mannosidases inhibitor Kifunensine (10 μM, GC17735, Glpbio) was used to inhibit N-glycan processing by EDEMs.

Cell culture and transfection. HEK293T and JURKAT were purchased from the ATCC. DND41 cells were contributed by laboratory of MD Anderson. HEK293T cells were cultured in DMEM supplemented with 10% fetal bovine serum and 1% penicillin/streptomycin (15140122, Thermo Fisher Scientific). JURKAT and DND41 cells were cultured in RPMI 1640 medium supplemented with 10% fetal bovine serum and 1% penicillin/streptomycin in a humidified incubator with 5% CO_2 at 37°C.

To establish HEK293T cells that stably expressed SFB-tagged γ-secretase proteins, the cells were transfected with the respective plasmids with polyethyleneimine (23966, Polysciences) and selected in DMEM supplemented with 2 μg/mL puromycin (A610593, Sangon) for at least 2 weeks. Protein expression confirmation was then performed by immunostaining and western blotting.

Generation of CRISPR-induced KO lines. The DOCK2-knockout cell lines were established via CRISPR/Cas9-mediated genome editing. The target sequences for CRISPR interference were designed via the Benchling tool (2021), ligated into a lentiCRISPR v2 plasmid (Addgene #52961, (1)) with the BsmBI restriction site, and packaged into lentivirus via cotransfection with the packaging plasmids pMD2.G (Addgene #12259) and psPAX2 (Addgene #12260) in HEK293T

cells. HEK293T and JURKAT cells were infected with the lentiCRISPR virus at the desired titer and then selected with puromycin. Individual clones were further expanded, and the knockout efficiency was confirmed by immunoblotting and DNA sequencing. The sgRNA sequences for DOCK2 were as follows: sg1: 5'-CACCGAAAGCTGACAAGGAGCGGCA; sg2: 5'-CACCGTCTCTCAGGAACCTTACCCA.

Tandem affinity purification (TAP). TAP purification was performed as previously described (2). Briefly, 1×10^8 HEK293T cells stably expressing cSFB-tagged γ-secretase components were lysed in 5 mL of NETN buffer (with protease inhibitors) at 4°C for 30 min, followed by TurboNuclease treatment. The lysates were then incubated with streptavidin-conjugated beads (17-5113-01, GE) for 2 h at 4°C. After washing with NETN buffer, the bound proteins were eluted with NETN buffer containing 2 mg/mL biotin (B4501, Sigma) for 2 h at 4°C. The eluates were then incubated with S-protein beads (69704, EMD Millipore) for 4 h. The beads were washed three times with NETN buffer and subjected to SDS–PAGE, followed by Coomassie blue staining. The whole bands were excised and subjected to in-gel trypsin digestion and MS analysis.

LC–MS. LC and MS were performed as previously described (3). Briefly, the trypsin-digested peptides were dried and reconstituted in 5 mL of high-performance liquid chromatography solvent A (2.5% acetonitrile and 0.1% formic acid). After the high-performance liquid chromatography capillary column was equilibrated, each sample was loaded onto the column via an autosampler. A gradient was formed, and the peptides were eluted with increasing concentrations of solvent B (97.5% acetonitrile and 0.1% formic acid). The eluted peptides were subjected to electrospray ionization and then analyzed via an Orbitrap Fusion Lumos Tribrid mass spectrometer (Thermo Fisher Scientific). The data-dependent acquisition mode was used. Peptide sequences and, hence, protein identities were determined by matching fragmentation patterns in protein databases via the Mascot software program (Matrix Science). The database searched was Swiss-Prot (*Homo sapiens*). Spectral matches were filtered such that the false discovery rate was less than 1% at the peptide level via the target-decoy method (4), and protein inference was considered to follow the general rules (5), with manual annotation applied when necessary.

Profiling of N-linked glycans from NCSTN. On-beads digestion: The NCSTN N-glycosylation sites and glycan structures were detected as previously reported (6, 7) with modifications. Briefly, NCSTN was purified from WT and DOCK2-KO JURKAT cells. A sufficient amount of protein was then taken for pre-mass spectrometry treatment, digestion with trypsin according to the ratio of 1:20 (w/w). Incubate on a 37°C shaker at 200 rpm for more than 16 h. The peptides were subsequently desalted using C18 cartridges (Waters, MA, USA) and dried using a Speed Vac.

Enrichment of N-Linked peptides using HILIC method: 5 mg click maltose material was washed three times with 80% ACN/1% TFA, and the digested proteins were re-dissolved in the loading buffer (80% ACN/1% TFA). The digests were mixed with the HILIC materials and the resulting suspension was shaken for 45 min. Then, the mixture was pipetted into a HILIC tip and washed with 40μL loading buffer twice. Finally, the enriched N-Linked peptides were eluted with 100 μL of 30% ACN/1%FA twice. The peptides were desalted using HLB C18 cartridges (Waters, MA, USA) following the manufacturer's protocol and dried before MS analysis.

LC-MS/MS analysis of the N-Linked peptides: The peptides were solubilized using 20 μLof 0.1% FA (v/v) and 15 μL was injected to an LC–MS/MS system which consists of a DionexTM UltiMate 3000 liquidchromatography and a Q Exactive HFTM mass spectrometer (Thermo Fisher Scientific, USA). Peptide separation was performed on a capillary analytical column (15 cm length, 150 μm i.d.) packed in-house with reverse phase C18 resins (1.8 μm, 150-Å pore size, Dr Maisch GmbH, Germany) with a 60 min gradient from 9% to 80% (v/v) ACN in 0.1% (v/v) FA.

Data analysis: The Glyco-Decipher (version 1.04) software5(6) was used for the interpretation of intact glycopeptides, enzyme was set as trypsin with up to 3 missed cleavage sites. Carbamidomethyl (C) was set as fixed modification and oxidation (M) was set as variable modification. For the peptide backbone identification, the presence of N-X-S/T motif was required. The mass torlerence for precursor ion and fragment ion was set to 10 ppm and 20 ppm. FDR threshold was set to 1% at both spectrum level for PSM identification and peptide level for peptide sequence identification.

Bioinformatic analyses. MS data analysis was performed via the SAINT algorithm as previously described to assign quality scores for the identified PPIs. A SAINT score was assigned to each

identified interaction, and any interaction with a SAINT score > 0.9 and raw average spectral counts greater than 5, as well as a frequency <20% in the CRAPome database (V1.1, 2014.1) (8), was considered to indicate an HCIP. The overall and individual interactomes of the γ-secretase core components were enriched in signaling pathways and functional categories according to the HCIP datasets. P values were estimated via the knowledge base included with the Ingenuity Pathway Analysis software program (Ingenuity Systems, USA), which contains findings and annotations from multiple sources, including the Gene Ontology, KEGG pathway, and PANTHER pathway databases. Only statistically significant correlations ($P < 0.05$) are shown. Cancer patient datasets were downloaded from cBioPortal (9, 10), and exome sequencing data from 130 T-ALL patients were obtained (11). Proteomaps of γ-secretase HCIPs were generated via online tools (12). The potential NCSTN glycosylation sites were predicted via NetNglyc (13).

Cross-linking mass spectrometry (XL-MS). XL-MS was performed as previously described(14). The SFB-tagged NCSTN overexpressing HEK293T cells were first treated with DSSO for cross-linking. After quenching the reaction, the cell was lysed, immunoaffinity pulldown using FLAG magnetic beads, followed with trypsin digestion and sample preparation for mass spectrometry as described above in LC-MS section. Data are processed using Proteome Discover 2.4 and the identified protein that cross-linked with NCSTN were used for probing protein-protein interactions.

Cell-based GVP reporter gene assay. γ-Secretase activity was evaluated via a previously described cell-based reporter gene assay (15). A cDNA encoding the DNA binding/transactivation domain GVP was amplified by PCR and cloned and inserted into NOTCH1 ΔE, generating the NOTCH1 ΔE-GVP construct (16). The upstream activator sequence (UAS) of Gal4 was cloned and inserted into a pGL3-luc vector, generating the UAS-luc reporter gene construct. HEK293T cells were transiently cotransfected with a mixture of DNA (450 ng/well) in a 24-well plate containing the reporter construct (UAS-luc, 100 ng/well), Renilla-luc (50 ng/well), substrate (NOTCH1 ΔE-GVP, 100 ng/well) and candidate constructs or empty vector (200 ng/well). NOTCH1 ΔE-GVP, a truncated form constitutively cleaved at S2 and S3 sites, releases an intracellular domain containing a Gal4VP16 (GVP) moiety upon γ-secretase cleavage (S3). This

fragment translocates to the nucleus and activates luciferase expression under the UAS promoter. After 24 h of culture, the reporter gene activity was assayed with a dual-luciferase assay kit (11402ES60, Yeasen) and normalized to Renilla activity.

Western blotting and immunoprecipitation. The cells were washed twice with phosphate-buffered saline (PBS) and dissolved in NETN lysis buffer (20 mM Tris-HCl, pH 8.0; 100 mM NaCl; 0.5% NP-40; and 1 mM EDTA) supplemented with protease and phosphatase inhibitors. Whole-cell lysates were subjected to SDS–PAGE (ExpressPlus™ PAGE Gel, M42015C, GenScript) and immunoblotted with specific antibodies.

For immunoprecipitation, 1×10^7 cells were lysed with NETN buffer on ice for 30 min. The lysates were then incubated with 30 μL of conjugated S-beads (for the SFB-tagged pull-down assay) for 2 h at 4°C or incubated with antibodies against endogenous proteins for 1 h at 4°C, followed by the addition of 20 μL of protein A/G agarose and incubation for 2 h at 4°C. The immunoprecipitates were washed with lysis buffer three times before immunoblotting analysis.

The following primary antibodies were used: rabbit anti-DOCK2 (10127S, CST), rabbit anti-NCSTN (14071-1-AP, Proteintech, RRID: AB_2149583), THE™ HA Tag (A01244, Genscript), THE™ c-MYC Tag (A00704, Genscript), ANTI-FLAG® M2 antibody (B3111, Sigma–Aldrich, RRID:AB_2910145), NICD1 (4147S, CST), PSEN1-NTF (MAB1563, Millipore, RRID:AB_11215630), CAV1 (3267S, CST), GOPC (8576S, CST) and rabbit anti-β-Actin (AC026, ABclonal, RRID:AB_2768234). The following secondary antibodies were used: Goat Anti-Mouse IgG Antibody (H&L) [HRP] (A00160, Genscript), Goat Anti-Rabbit IgG Antibody (H&L) [HRP] (A00178, Genscript).

Lectin blotting. Immunoprecipitated proteins were subjected to SDS–PAGE and transferred to PVDF membranes. The membranes were first incubated in 5% BSA/PBST for 1 h. Biotin and streptavidin blocking was performed via a commercial kit (SP-2002, Vector Laboratories) according to the manufacturer's instructions. The membranes were incubated with the respective biotinylated lectin (0.5 μg/mL in lectin buffer (10 mM HEPES, pH 7.5, 150 mM NaCl, 0.1 mM CaCl2, and only ConA 10 μM MnCl2)) for 2 h at room temperature. After three washing steps, the blots were incubated with a streptavidin-HRP conjugate (0.5 μg/mL, diluted in PBST) at room

temperature for 30 min. After three additional washing steps, the blots were imaged via conventional ECL chemistry (P10200, NCM).

Golgi apparatus protein extraction. Golgi apparatus proteins were isolated according to the manufacturer's instructions (BB-314533, BestBio). Briefly, the cells were first lysed and homogenized in cold buffer A. The supernatants were collected, and WT buffer was added. Following a series of centrifuges, the pellets were finally dissolved in protein extraction buffer D for subsequent use.

Quantitative real-time PCR. Total RNA was isolated from cells via TRIzol reagent (9109, Takara), and cDNA synthesis was performed via the addition of 1 μg of total RNA with HiScript III reverse transcriptase (R212-02, Vazyme). The mRNA levels of specific genes were quantified via SYBR Green qPCR according to the manufacturer's instructions on a Jena Qtower 3G quantitative PCR system. The relative mRNA levels were determined via the comparative Ct method, with Actin used as the reference gene, following the formula $2^{-\Delta\Delta Ct}$. The primers that were used were as follows: *HES1*-F: 5'-CCTGTCATCCCCGTCTACAC; *HES1*-R: 5'-CACATGGAGTCCGCCGTAA; *HES4*-F: 5'-AGACACTCGTGGAAGCCG; *HES4*-R: 5'-ATGACCGTGAGACACCTGC; *c-MYC*-F: 5'-GGCTCCTGGCAAAAGGTCA; *c-MYC*-R: 5'-CTGCGTAGTTGTGCTGATGT; *Actin*-F: 5'-TTGCCGACAGGATGCAGAAGGA; *Actin*-R: 5'-AGGTGGACAGCGAGGCCAGGAT.

Immunofluorescence. The cells were seeded in cell culture dishes, fixed with 4% paraformaldehyde at room temperature for 10 min, permeabilized for 5 min with 0.1% Triton X-100, washed with PBS and blocked in 5% BSA in PBS for 30 min before being labeled with anti-NCSTN (14071-1-AP, Proteintech, RRID: AB_2149583), anti-ERp72 (14712-1-AP, Proteintech, RRID: AB_2160973), anti-GM130 (11308-1-AP, Proteintech, RRID: AB_2115327) primary antibodies or 647-conjugated DYKDDDDK tag Monoclonal antibody (CL647-66008, Proteintech) at room temperature for 1 h. After incubation, the cells were washed twice with PBS, stained with goat-anti-rabbit Alexa Fluor® 488-labeled IgG (ab150077, Abcam, RRID: AB_2630356) or goat-anti-mouse Alexa Fluor® 594-labeled IgG (ab150116, Abcam, RRID: AB_2650601) at room temperature for 1 h, and subjected to 4',6-diamidino-2-phenylindole (DAPI) staining (4083S,

CST). Coverslips were mounted using FluorSave™ Reagent (345789, Millipore). The cells were stained with DiI before primary antibody incubation for membrane staining. The cells were viewed via an Olympus FV3000 Microscope Imaging System (Olympus, Japan).

Cell Counting Kit-8 assay. Cell viability was measured via the Cell Counting Kit-8 (CCK-8) assay according to the manufacturer's instructions (C0005, TargetMol). JURKAT and 293T cells were incubated with vehicle or Kifunensine (GC17735, GLPBIO) at different concentrations as indicated for 36 h. Then, the cells were treated with the CCK-8 reagent and incubated for another hour. The absorbance was detected at 450 nm, and the related cell viability was measured.

hERG toxicity assessment. To evaluate the safety of Kifunensine, the whole-cell patch-clamp technique was employed to record hERG potassium currents from cultured cells. The amplitude of the hERG current was defined as the peak current during the repolarizing step. Vehicle control was applied first to establish a baseline. Cell stability was confirmed by recording for 120 s prior, only cells with stable baseline recordings were used. Currents in the presence of Kifunensine were recorded for approximately 5 minutes until a steady-state block was reached, after which five consecutive sweeps were captured. For concentration-response relationships, five cumulative concentrations were applied sequentially from lowest to highest.

Pharmacokinetics measurement. Mice (Jihui, China) were first administrated with Kifunensine (10 mg/kg) via tail vein. Then the plasma sample at different points were prepared. Calibration standards (0.5-1000 ng/mL) and quality control (QC) samples (1, 2, 50, and 800 ng/mL) were prepared by spiking the appropriate working solutions into blank plasma. For sample preparation, 50 µL of plasma (calibrators, QCs, or samples) was mixed with 5 µL of a blank solution (for study samples) or working solution (for calibrators and QCs). Then, 200 µL of acetonitrile/methanol (1:1, v/v) containing the internal standard was added to precipitate proteins. After vortex and centrifugation, the supernatant was diluted three-fold with water. A 2 µL aliquot of the processed sample was injected into the LC-MS/MS system for quantitative analysis.

Brain Penetration PK. Kifunensine (10 mg/kg) was administered via intravenous injection followed with plasma and brain tissue sample preparation at predetermined time points. Plasma and brain homogenate samples were processed by protein precipitation and analyzed using a

validated LC–MS/MS method. The unbound fraction in plasma (fu,plasma) and brain (fu,brain) was determined by equilibrium dialysis. Plasma samples and brain homogenates (prepared at 1:4 dilution) were spiked with the test compound and dialyzed against phosphate buffer (pH 7.4) at 37°C until equilibrium was reached.

RNA-sequencing and data analysis. Total RNA was extracted from WT DND41 cells treated with or without Kifunensine or GSI (DBZ) (10 μM for 24 h), as well as DOCK2-KO cells using RNAeasy Mini Kit (74104, QIAGEN), according to the manufacturer's protocol. Libraries for RNA sequencing were constructed using the Illumina Stranded Total RNA Prep with Ribo-Zero Plus kit. The quality and size distribution of the amplified libraries were assessed using a NanoDrop spectrophotometer (Thermo Fisher Scientific) and an Agilent 2100 Bioanalyzer, respectively. Sequencing was performed on Illumina NovaSeq X Plus using a paired-end run (Novogene). Raw sequencing data underwent quality control filtering to obtain high-quality reads. Differential gene expression analysis was performed using the DESeq2 R package. Genes with an adjusted p-value of less than 0.05 and an absolute log2 fold change greater than 1 were considered significantly differentially expressed.

Mouse model. All the animal experiments were performed in accordance with a protocol approved by the Institutional Animal Care and Use Committee of Westlake University (AP#20-023-LX). Five to ten mice were randomly assigned to each group for each study. WT and DOCK2-KO JURKAT cells (5×10^6) were separately resuspended in 100 μL of Matrigel (356237, Corning) diluted with PBS at a 1:1 ratio and subcutaneously injected into the left and right flanks, respectively, of anesthetized 6- to 8-week-old female BALB/c nude mice (Jihui, China). Starting on day 7, tumor formation was observed biweekly. The mice were euthanized after 4 weeks of injection, and the tumors were excised, photographed, and weighed.

For the invasion assay, a leukemia model was established in NSG mice (Jihui, China). WT and DOCK2-KO JURKAT-luc reporter cells (5×10^6) were resuspended in 100 μL of PBS and intravenously injected into 6- to 8-week-old female NSG mice via the tail vein. Starting on day 7, peripheral blood leukemia cells were analyzed by detecting the percentage of CD5+ cells via flow cytometry. Leukemia cells infiltration was monitored via bioluminescence imaging. At the end of

the study, the mice were euthanized, and the spleen tissues were excised, photographed, fixed in 4% paraformaldehyde, paraffin-embedded, and stained with hematoxylin and eosin or anti-CD5 antibodies.

For the evaluation of Kifunensine efficacy in vivo, 6- to 8-week-old female NSG mice were intravenously injected with 5×10^6 JURKAT cells or patient-derived leukemia cells and then randomly divided into the vehicle and Kifunensine groups (10 mg/kg). The progression of leukemia was evaluated by bioluminescence imaging, and the percentage of CD5-positive human cells in the peripheral blood was analyzed using flow cytometry. At the end of the study, spleen tissues were isolated and subjected to histological analysis.

In vivo bioluminescence imaging. To monitor tumor growth in living animals, the JURKAT cells that were used for the animal studies were transduced with firefly luciferase via lentiviral infection. Then, the cells were infected with the lentiCRISPR virus to knock out DOCK2, and these infected cells were intravenously engrafted into 6- to 8-week-old female NSG mice. For imaging analysis, the animals were intraperitoneally administered 150 mg/kg D-luciferin (40902ES01, Yeasen) and anesthetized with isoflurane. Tumor luciferase images were captured with an IVIS imaging system (PE IVIS Spectrum).

Histological analysis. Liver and spleen tissues were collected from mice in the different groups, fixed in 4% paraformaldehyde and immersed in fixative for 24 h. After being embedded in paraffin, 4-μm sections were prepared and placed on poly-L-lysine-coated slides. Morphological changes were analyzed via hematoxylin and eosin staining. Leukemia cell infiltration was analyzed by staining with anti-human CD5 antibodies.

Flow cytometry analysis. Peripheral blood was collected from NSG mice, and red blood cells were removed via RBC lysis (C3702, Beyotime). After washing with PBS, cells were labelled in suspension with FITC mouse anti-human CD5 (561896, BD Pharmingen) for 30 min at 4°C. Cells were then washed three times with PBS, the CD5 intensity was analyzed with a CytoFLEX6 flow cytometer and FlowJo software according to the manufacturer’s instructions.

References

1. Sanjana NE, Shalem O, and Zhang F. Improved vectors and genome-wide libraries for CRISPR screening. *Nat Methods.* 2014;11(8):783-4.
2. Bian W, Tang M, Jiang H, Xu W, Hao W, Sui Y, et al. Low-density-lipoprotein-receptor-related protein 1 mediates Notch pathway activation. *Developmental cell.* 2021;56(20):2902-19 e8.
3. Jiang H, Bian W, Sui Y, Li H, Zhao H, Wang W, et al. FBXO42 facilitates Notch signaling activation and global chromatin relaxation by promoting K63-linked polyubiquitination of RBPJ. *Sci Adv.* 2022;8(38):eabq4831.
4. Elias JE, and Gygi SP. Target-decoy search strategy for increased confidence in large-scale protein identifications by mass spectrometry. *Nat Methods.* 2007;4(3):207-14.
5. Nesvizhskii AI, and Aebersold R. Interpretation of shotgun proteomic data: the protein inference problem. *Mol Cell Proteomics.* 2005;4(10):1419-40.
6. Fang Z, Qin H, Mao J, Wang Z, Zhang N, Wang Y, et al. Glyco-Decipher enables glycan database-independent peptide matching and in-depth characterization of site-specific N-glycosylation. *Nature communications.* 2022;13(1):1900.
7. Qin H, Zhao L, Li R, Wu R, and Zou H. Size-selective enrichment of N-linked glycans using highly ordered mesoporous carbon material and detection by MALDI-TOF MS. *Anal Chem.* 2011;83(20):7721-8.
8. Mellacheruvu D, Wright Z, Couzens AL, Lambert JP, St-Denis NA, Li T, et al. The CRAPome: a contaminant repository for affinity purification-mass spectrometry data. *Nat Methods.* 2013;10(8):730-6.
9. Cerami E, Gao J, Dogrusoz U, Gross BE, Sumer SO, Aksoy BA, et al. The cBio cancer genomics portal: an open platform for exploring multidimensional cancer genomics data. *Cancer Discov.* 2012;2(5):401-4.
10. Gao J, Aksoy BA, Dogrusoz U, Dresdner G, Gross B, Sumer SO, et al. Integrative analysis of complex cancer genomics and clinical profiles using the cBioPortal. *Sci Signal.* 2013;6(269):pl1.
11. Chen B, Jiang L, Zhong ML, Li JF, Li BS, Peng LJ, et al. Identification of fusion genes and characterization of transcriptome features in T-cell acute lymphoblastic leukemia. *Proceedings of the National Academy of Sciences of the United States of America.* 2018;115(2):373-8.
12. Liebermeister W, Noor E, Flamholz A, Davidi D, Bernhardt J, and Milo R. Visual account of protein investment in cellular functions. *Proceedings of the National Academy of Sciences of the United States of America.* 2014;111(23):8488-93.
13. Gupta R, and Brunak S. Prediction of glycosylation across the human proteome and the correlation to protein function. *Pac Symp Biocomput.* 2002:310-22.
14. Khan K, Baleanu-Gogonea C, Willard B, Gogonea V, and Fox PL. An optimized protocol for in vitro and in cellulo structural determination of the multi-tRNA synthetase complex by cross-linking mass spectrometry. *STAR Protoc.* 2022;3(1):101201.
15. Karlstrom H, Bergman A, Lendahl U, Naslund J, and Lundkvist J. A sensitive and quantitative assay for measuring cleavage of presenilin substrates. *J Biol Chem.* 2002;277(9):6763-6.
16. Taniguchi Y, Karlstrom H, Lundkvist J, Mizutani T, Otaka A, Vestling M, et al. Notch receptor cleavage depends on but is not directly executed by presenilins. *Proceedings of the National Academy of Sciences of the United States of America.* 2002;99(6):4014-9.

Supplemental Data

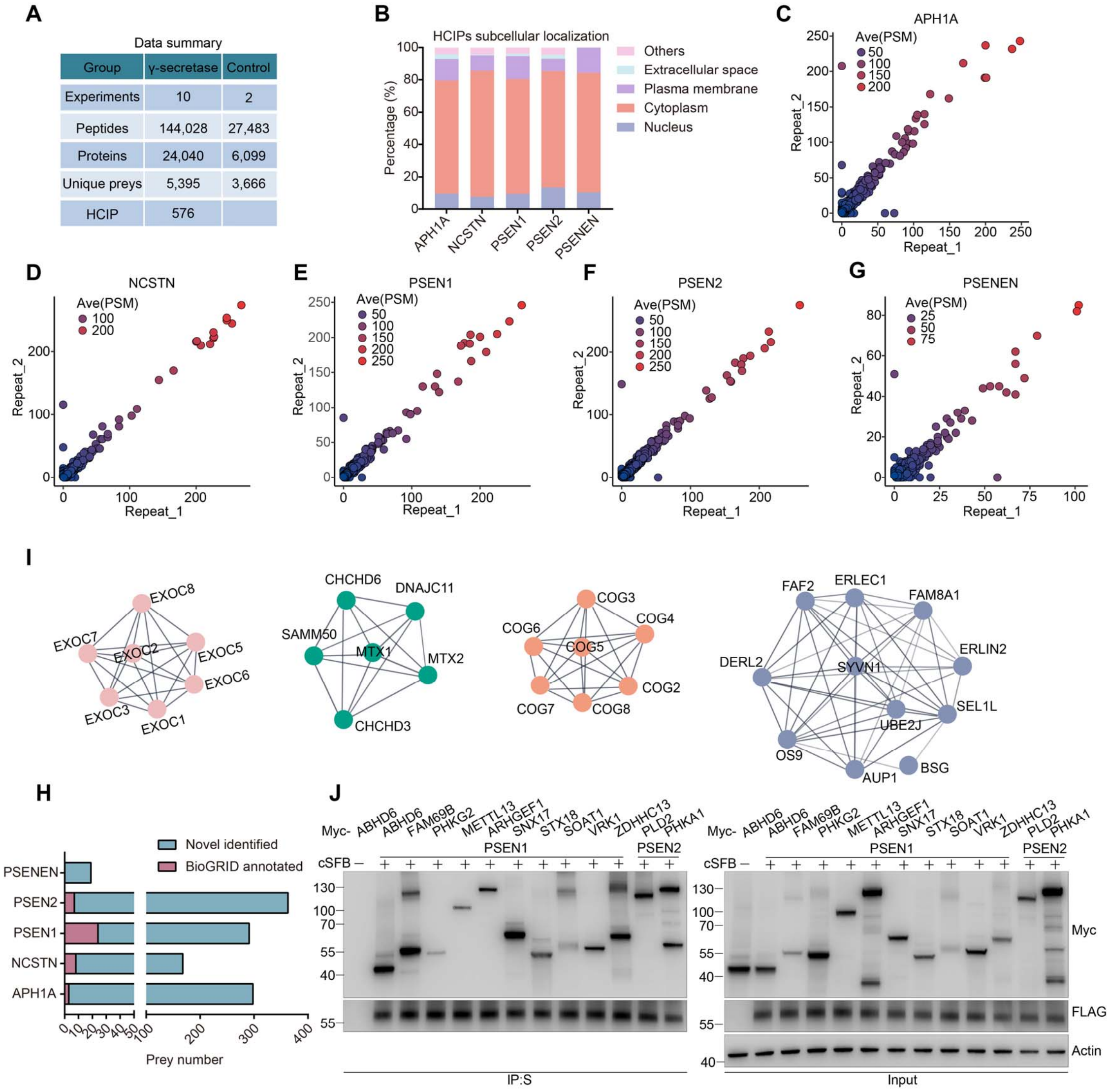

Supplemental Figure 1. Proteomic analysis and validation of the γ-secretase protein–protein interaction network. (A) Summary of mass spectrometry data and high-confidence interacting protein (HCIP) selection criteria. Total numbers of identified peptides and proteins are shown. HCIPs were defined using a SAINT score cutoff > 0.85 and CRAPome frequency < 20%. **(B)** Subcellular localization distribution of prey proteins identified in each bait purification. **(C–G)** Reproducibility assessment of the γ-secretase interaction network. Two biological replicates of TAP-MS were performed, and prey proteins were compared to evaluate reproducibility at the protein level. **(H)** Overlap between HCIPs identified in this study and previously reported interactors in the BioGRID database. **(I)** Protein complexes enriched among HCIPs, as annotated using the Corum database. **(J)** Co-immunoprecipitation validation of selected PSEN1- and PSEN2-interacting HCIPs. HEK293T cells were co-transfected with Myc-tagged candidate interactors and cSFB-tagged PSEN1 or PSEN2. Lysates were incubated with S beads, and bound proteins were detected using anti-FLAG (bait) and anti-Myc (prey) antibodies. Input lanes represent 5% of total lysate; actin served as a loading control. J, n=3.

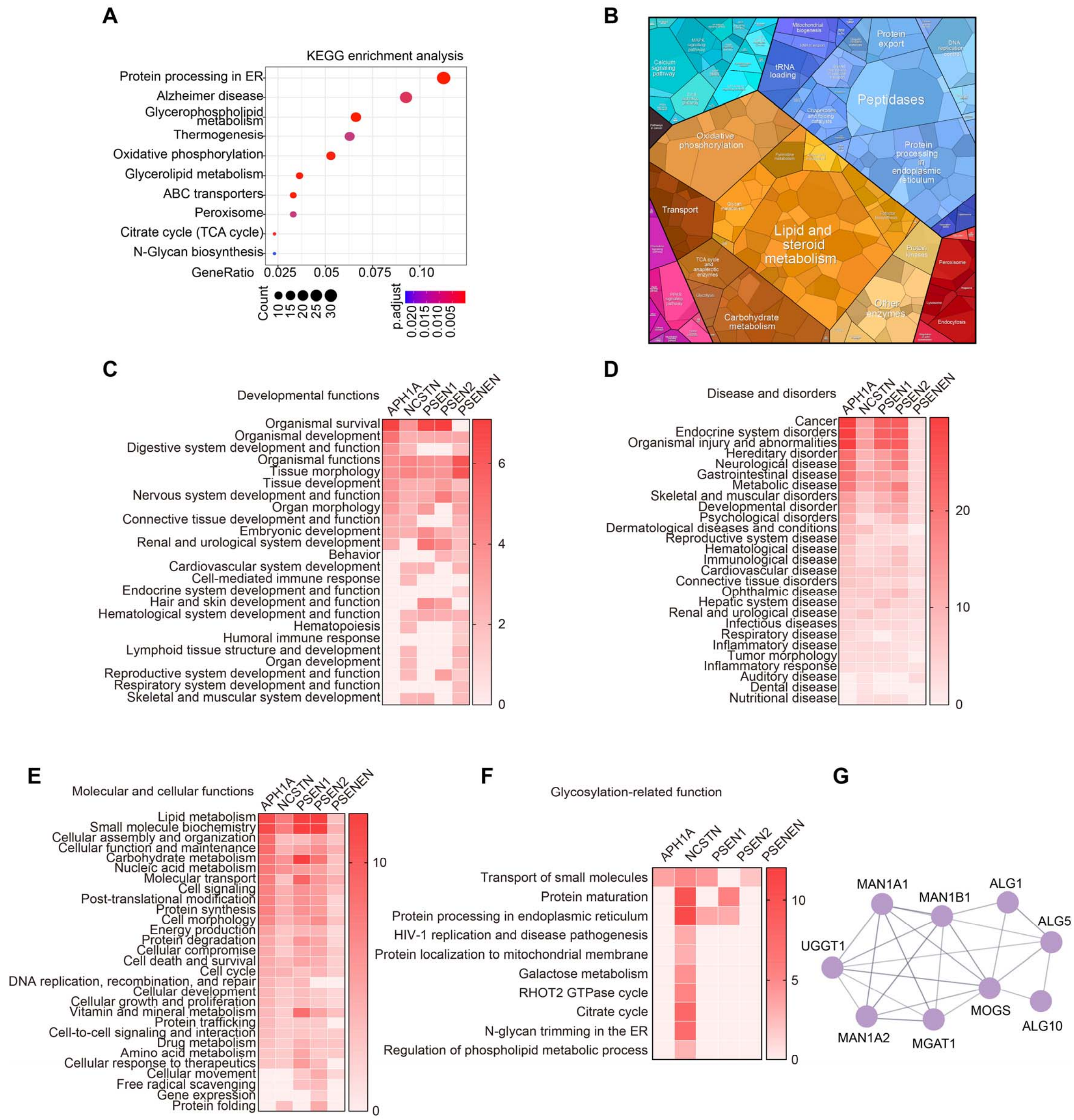

Supplemental Figure 2. Pathway and Gene Ontology (GO) enrichment analysis of γ-secretase HCIPs. (A and B) Enrichment analysis of high-confidence interacting proteins (HCIPs) using the Kyoto Encyclopedia of Genes and Genomes (KEGG, A) and Proteomaps (B) databases. Each polygon represents a significantly enriched biological process category among HCIPs. **(C–F)** Functional enrichment analysis of γ-secretase HCIPs in molecular and cellular functions (C), developmental processes (D), diseases and disorders (E), and glycosylation-related functions (F). Only statistically significant terms ($P < 0.05$) are displayed; the $-\log_{10}$(P value) and associated HCIPs are indicated for each function. **(G)** Protein interaction subnetwork of glycosylation-related NCSTN interactors, as reconstructed from the STRING database.

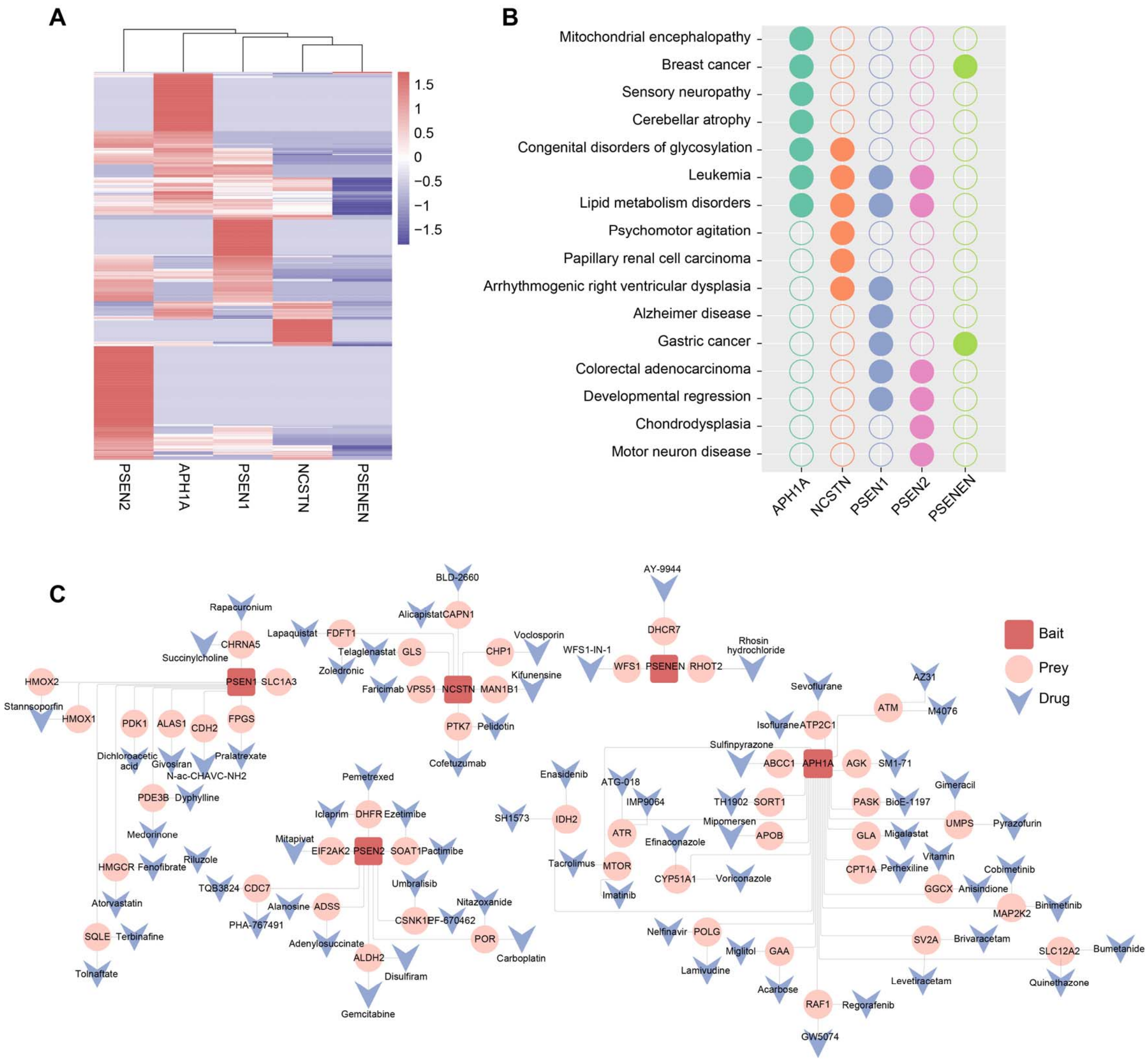

Supplemental Figure 3. Differential analysis of the γ-secretase subunits interaction networks. (A) Heatmap displaying distinct interaction profiles among core components of the γ-secretase complex. **(B)** Bubble plot illustrating disease terms significantly associated with interactors of each γ-secretase subunit. Filled bubbles represent enriched terms; empty bubbles indicate non-enriched terms. **(C)** Drug-target interaction network highlighting potential therapeutic agents targeting HCIPs identified in this study.

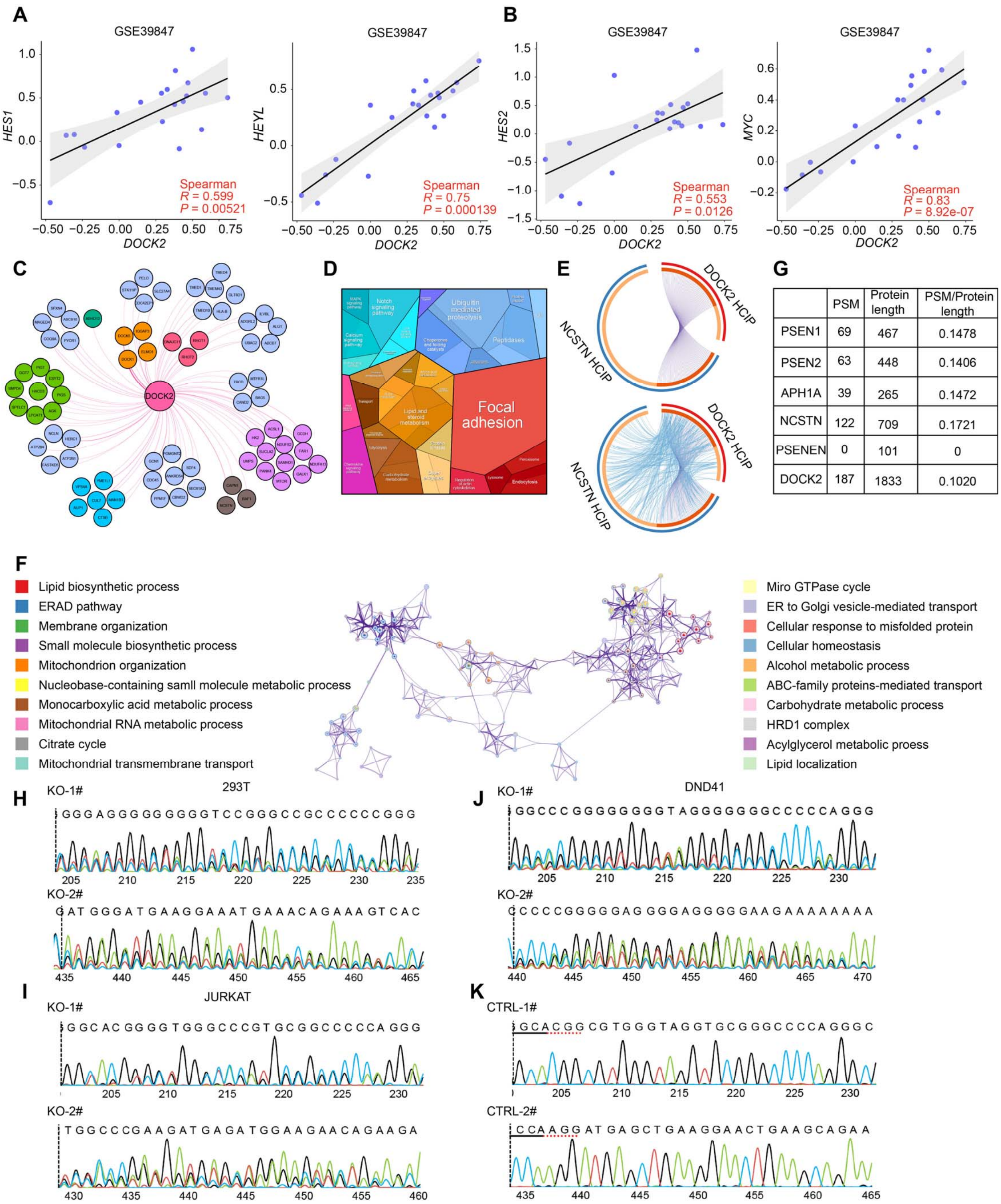

	PSM	Protein length	PSM/Protein length
PSEN1	69	467	0.1478
PSEN2	63	448	0.1406
APH1A	39	265	0.1472
NCSTN	122	709	0.1721
PSENEN	0	101	0
DOCK2	187	1833	0.1020

Supplemental Figure 4. Protein interaction profiling of DOCK2. (A and B) Correlation analysis between DOCK2 expression and *HES1*, *HEYL, HES2 and MYC* in acute lymphoblastic leukemia (ALL) patients from GEO database. **(C)** Functional interaction network of DOCK2. Interacting proteins are grouped and color-coded by biological process. **(D)** Proteomaps functional annotation of DOCK2 high-confidence interacting proteins (HCIPs). Each polygon represents a significantly enriched biological process category. **(E)** Circos plot illustrating overlap between NCSTN and DOCK2 interactors. Purple lines connect shared genes; blue lines link distinct genes participating in common ontology terms. **(F)** Shared GO terms between NCSTN and DOCK2 interactomes, analyzed using Metascape (https://metascape.org). Enriched terms are represented as pie charts color-coded by gene list origin, with sector size proportional to hit count from each dataset. **(G)** PSM value and PSM/protein length ratio of cross-linking MS data for

the γ-secretase complex. **(H-K)** Sanger sequencing alignment of DOCK2 KO cell lines for the knockout efficiency confirmation.

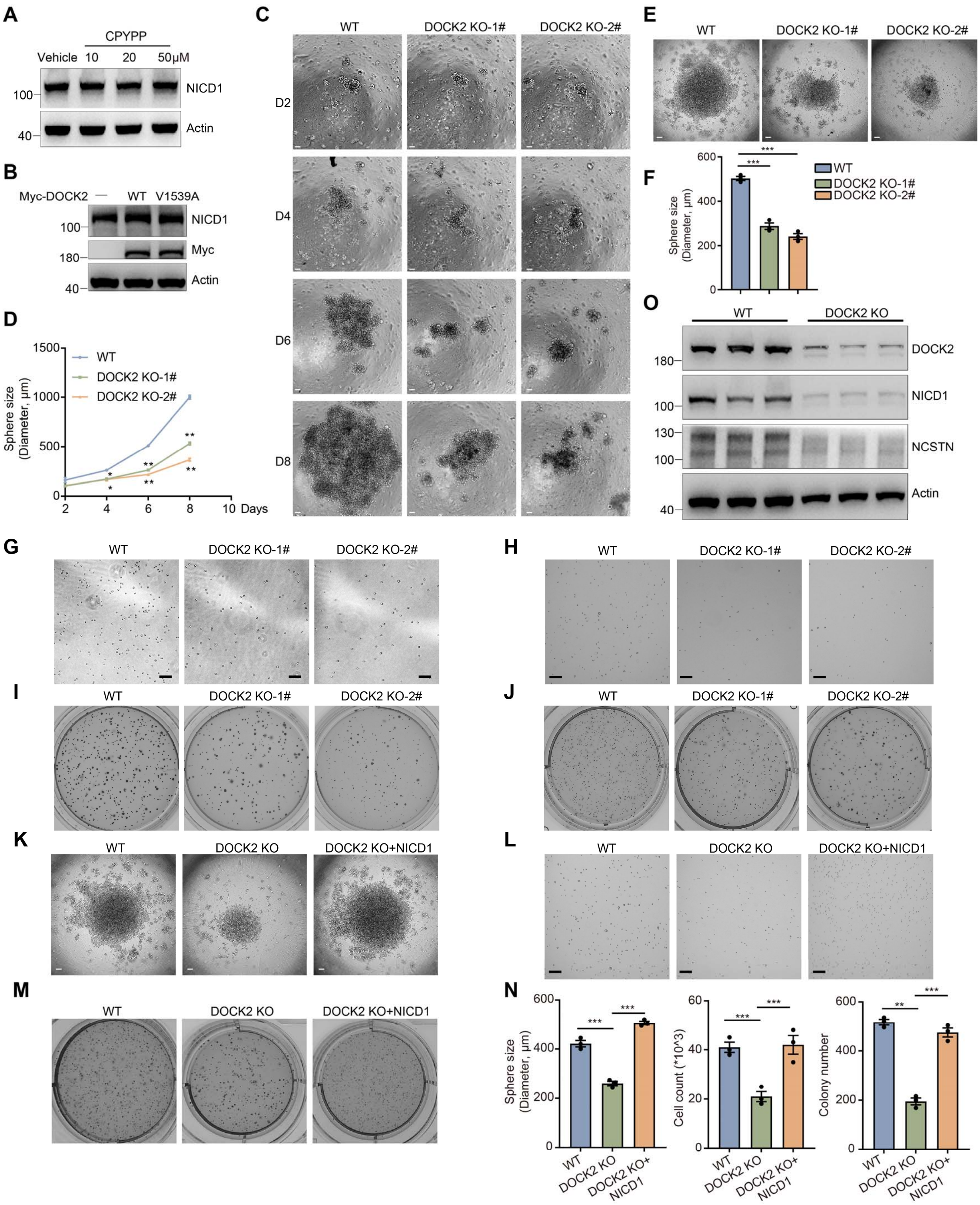

Supplemental Figure 5. DOCK2 knockout impairs tumor growth in vitro. (A) Western blot analysis of NICD1 levels in JURKAT cells treated with the DOCK2 enzymatic inhibitor CPYPP. **(B)** NICD1 levels in HEK293T cells transfected with wild-type DOCK2 or a catalytically dead mutant, assessed by western blot. **(C–F)** Invasion capacity of WT and DOCK2-KO JURKAT (C, D) and DND41 (E, F) cells evaluated using a 3D Matrigel culture system. Scale bars: 50 μm; sphere diameters were quantified. **(G** and **H)** Transwell migration assay of WT and DOCK2-KO JURKAT (G) and DND41 (H) cells. Scale bars: 200 μm. **(I** and **J)** Anchorage-independent growth of WT and DOCK2-KO JURKAT (I) and DND41 (J) cells measured by soft agar colony formation assay. **(K–N)** Rescue experiments in DND41 cells: DOCK2-KO cells with or without NICD1

overexpression were subjected to 3D Matrigel invasion (K; scale bars: 50 μm), Transwell migration (L; scale bars: 200 μm), and soft agar colony formation (M) assays to evaluate NICD1-mediated reversal of DOCK2-KO phenotypes. **(O)** Western blot analysis of NICD1 and NCSTN levels in tumors derived from WT versus DOCK2-KO JURKAT cells. Mean ± SEM; A-N, n=3, * $P < 0.05$, ** $P < 0.01$, *** $P < 0.001$, one-way ANOVA.

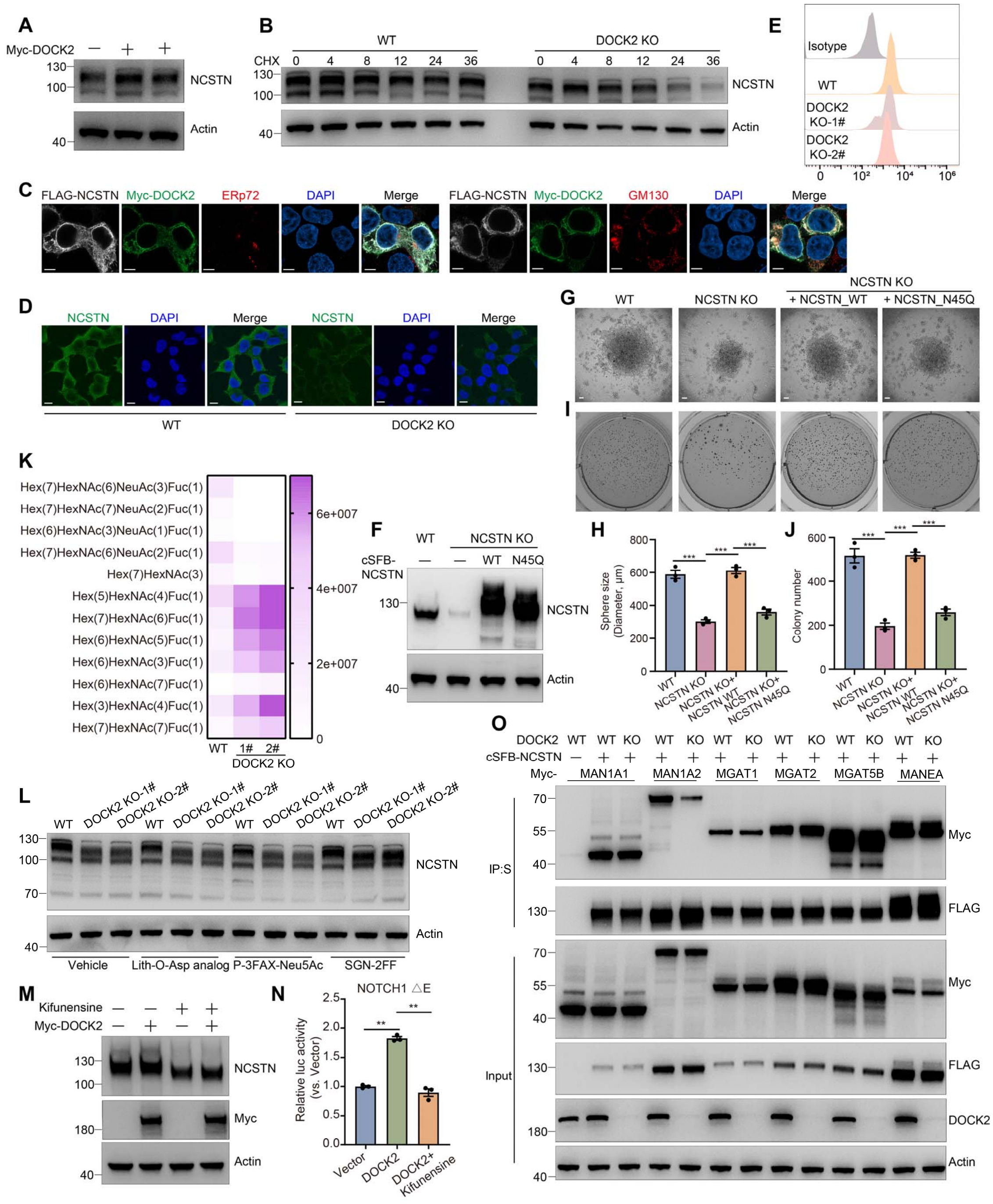

Supplemental Figure 6. DOCK2 regulates NCSTN N-glycosylation at N45 site. (A) Western blot analysis of NCSTN protein levels in DOCK2-overexpressing HEK293T cells. **(B)** NCSTN protein stability assay: WT and DOCK2-KO HEK293T cells treated with cycloheximide (CHX, 50 μg/mL) for indicated durations. Endogenous NCSTN levels were assessed by western blot. **(C)** Immunofluorescence staining of FLAG-NCSTN, Myc-DOCK2 with ER marker ERp72 or Golgi marker GM130 in HEK293T cells. **(D)** Immunofluorescence staining of NCSTN (green) and DAPI (blue) in fixed and permeabilized WT and DOCK2-KO HEK293T cells. Scale bars: 10 μm. **(E)** Flow cytometry analysis of membrane NCSTN levels in intact WT and DOCK2-KO JURKAT cells. X-axis: APC-NCSTN fluorescence intensity. **(F)** Western blot validation of NCSTN expression in NCSTN-KO DND41 cells rescued with WT or N45Q mutant NCSTN. **(G and H)** 3D

Matrigel culture assay in NCSTN-KO DND41 cells expressing WT NCSTN or it N45Q mutant. Scale bars: 50 μm; sphere diameters were quantified. **(I and J)** Soft agar colony formation assay in NCSTN-KO DND41 cells reconstituted with WT NCSTN or it N45Q mutant. **(K)** Heatmap of differentially expressed N-glycans on NCSTN N45 site in WT versus DOCK2-KO JURKAT cells identified by N-glycoproteomics. The value is corresponding to the ion intensity in Table S6. **(L)** Western blot analysis of NCSTN in WT and DOCK2-KO JURKAT cells treated with sialylation and fucosylation inhibitors for 24 h. Lith-O-Asp analog (10 μM), P-3FAX-Neu5Ac (50 μM) were used as sialylation inhibitors and SGN-2FF (100 μM) was used as fucosylation inhibitor. **(M)** Western blot of NCSTN in WT and DOCK2-overexpressing HEK293T cells treated with or without Kifunensine (10 μM) for 24 h. **(N)** γ-Secretase activity assay (GVP-UAS) evaluating NOTCH1 ΔE processing in DOCK2-overexpressing HEK293T cells treated with Kifunensine (10 μM) for 24 h. **(O)** Co-immunoprecipitation assay: Lysates from WT and DOCK2-KO HEK293T cells co-expressing cSFB-NCSTN and Myc-tagged glycosylation-related proteins were incubated with S beads and immunoblotted with anti-FLAG or anti-Myc antibodies. Mean ± SEM; A-I, K-N, n=3, ** $P < 0.01$, *** $P < 0.001$, one-way ANOVA.

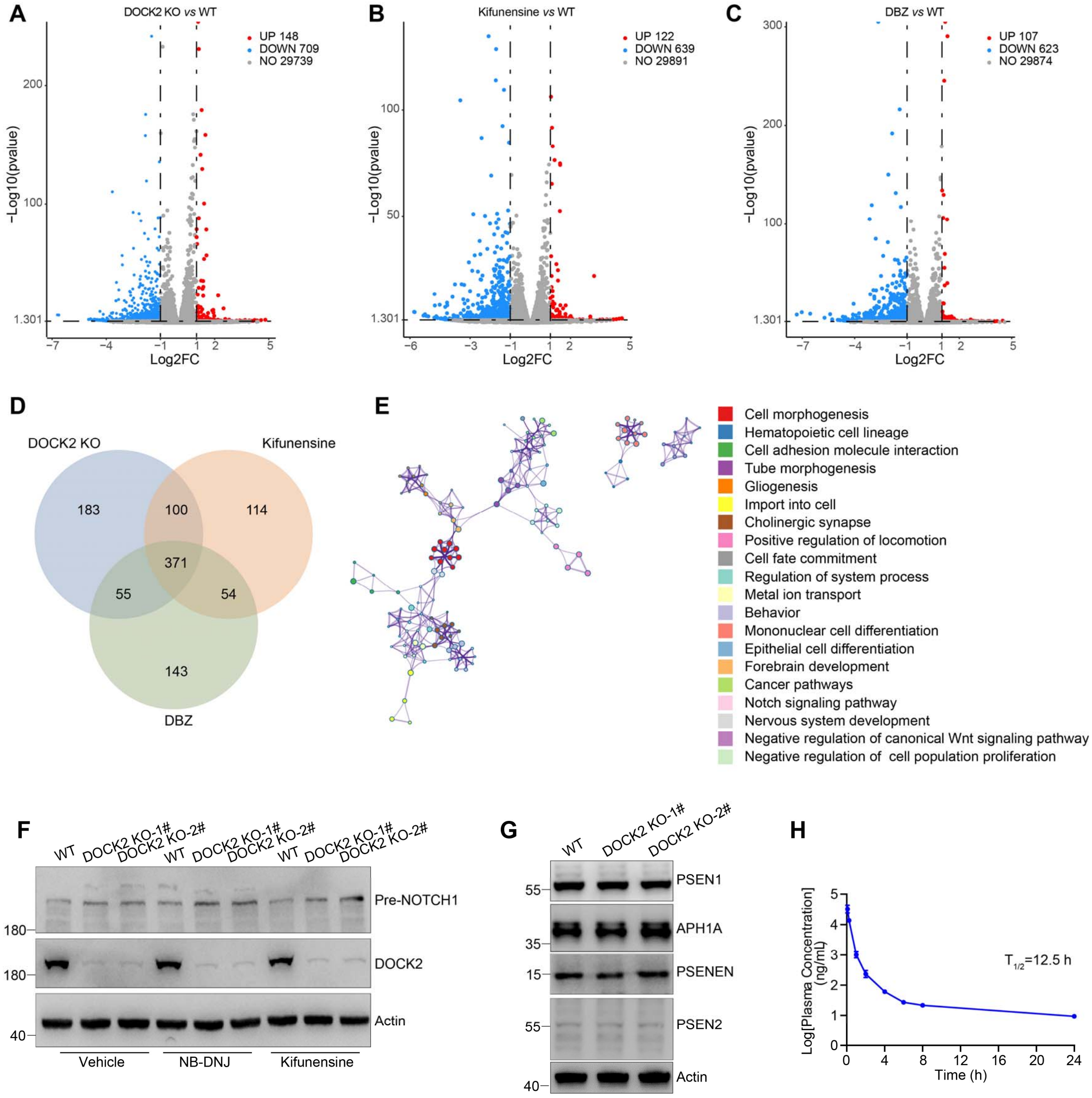

Supplemental Figure 7. DOCK2 knockout does not affect glycosylation of other Notch pathway components. (A-C) Volcano plots of differentiated genes derived from RNA-seq data in DND41 cells with indicated treatment. **(D)**Venn diagram showing overlap of downregulated genes in each group compared with WT. **(E)** Enrichment analysis of the overlapped genes in (D) using metascape website. **(F)** Western blot analysis of NOTCH1 precursor in WT and DOCK2-KO JURKAT cells treated with N-butyl-deoxynojirimycin (NB-DNJ, 1 mM) or Kifunensine (10 μM) for 24 h. **(G)** Western blot of core γ-secretase components (PSEN1, PSEN2, APH1A, PSENEN) in WT and DOCK2-KO DND41 cells to assess their expression and glycosylation status. (**H**) Pharmacokinetic profile of Kifunensine after intravenous (i.v.) administration. A-G, n=3.

Supplemental Table 1: Complete protein identification list from TAP-MS of γ-secretase subunits.

Full list of proteins identified by TAP-MS for each core component of the human γ-secretase complex.

Supplemental Table 2: High-confidence interacting proteins (HCIPs) of γ-secretase subunits.

Curated list of HCIPs identified for each γ-secretase subunit following stringent filtering using SAINT algorithm.

Supplemental Table 3: Gene Ontology and KEGG pathway enrichment of HCIPs.

Human γ-secretase core components were enriched in gene ontology and KEGG using their HCIP sets.

Supplemental Table 4: Functional enrichment analysis of HCIPs.

Human γ-secretase core components were enriched in molecular and cellular functions, diseases and disorders, and developmental functions using their HCIP sets.

Supplemental Table 5: Drug repurposing analysis targeting HCIPs.

Integration of HCIPs with the Drug Repurposing Hub database, listing FDA-approved drugs with potential targeting relevance to γ-secretase interactors.

Supplemental Table 6: Glycoproteomic profiling of WT and DOCK2-KO cells.

Site-specific N-glycosylation data for NCSTN, including glycan compositions and abundances, in wild-type and DOCK2-knockout cells.

Supplemental Table 7: Pharmacokinetic parameters of Kifunensine.

Metabolic stability and pharmacokinetic properties of Kifunensine.
