# Supplementary material for: Targeting mannosylation of nicastrin N-glycans attenuates γ-secretase activity and notch-dependent leukemia progression
Source: J Clin Invest. 2026 Oct 1;136(19):e203202. doi: 10.1172/JCI203202 (PMC13626847; doi:10.1172/JCI203202)

Full unedited blot
for Figure 3C

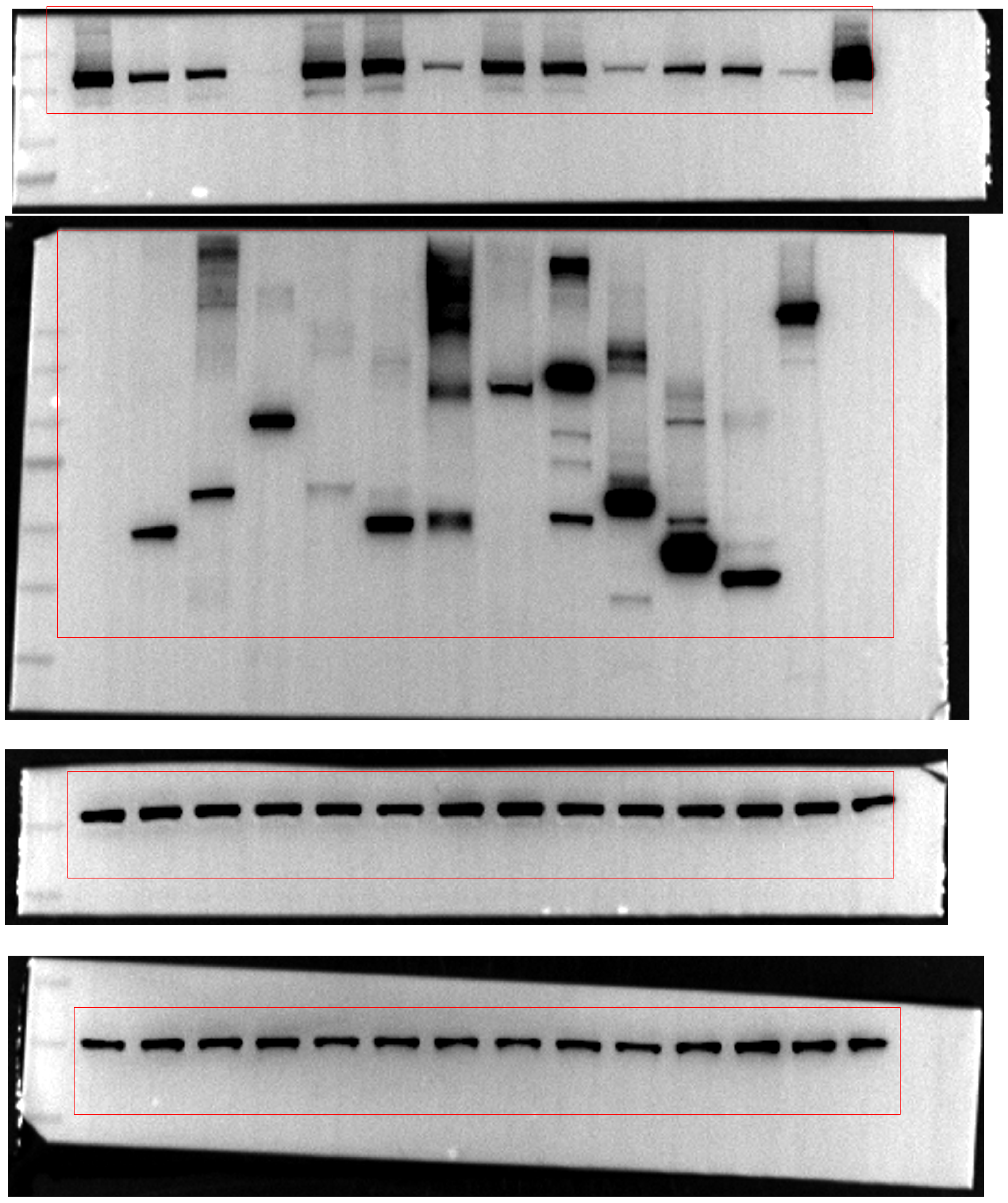

Full unedited blot
for Figure 3D

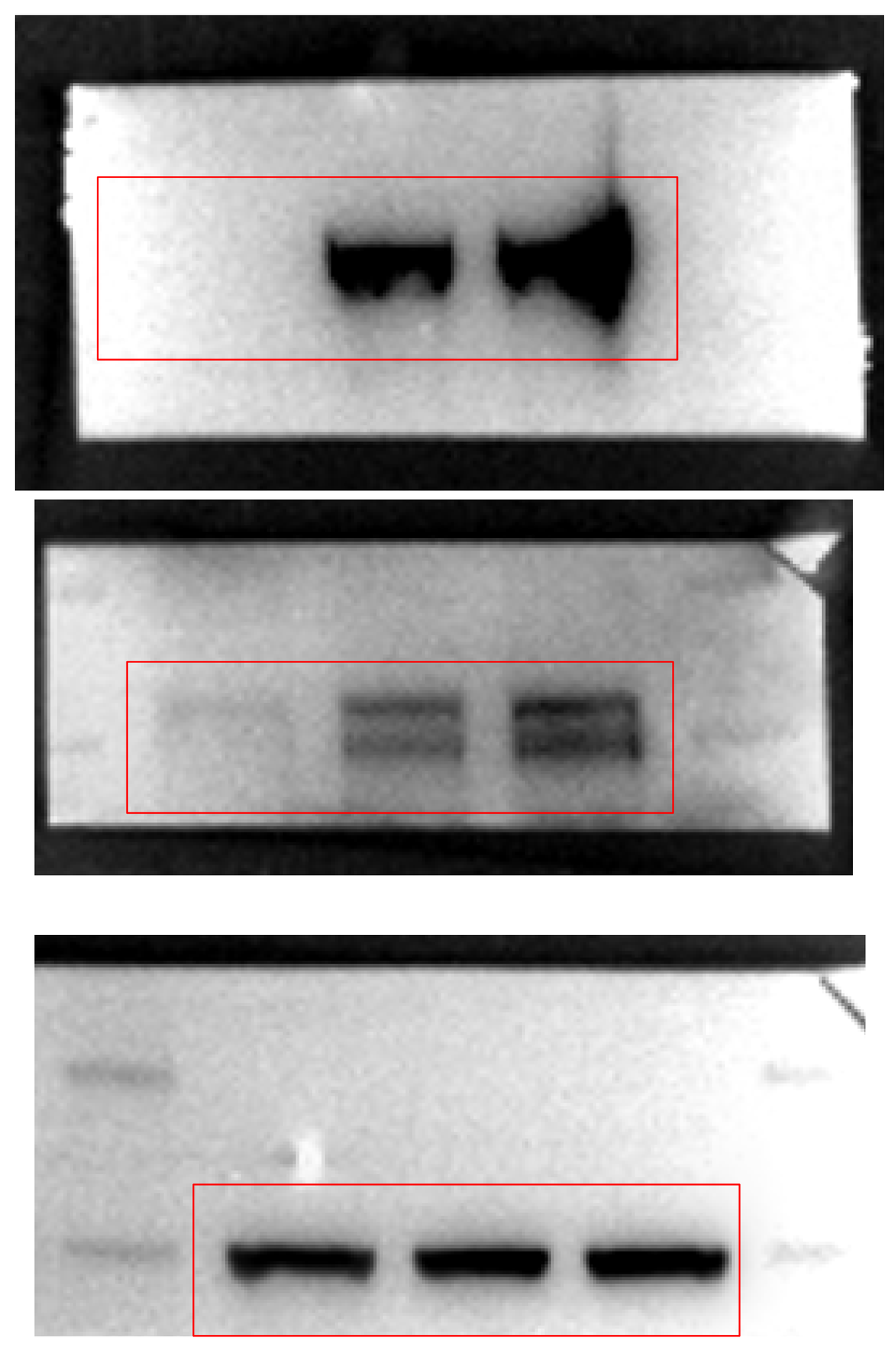

Full unedited blot
for Figure 3F

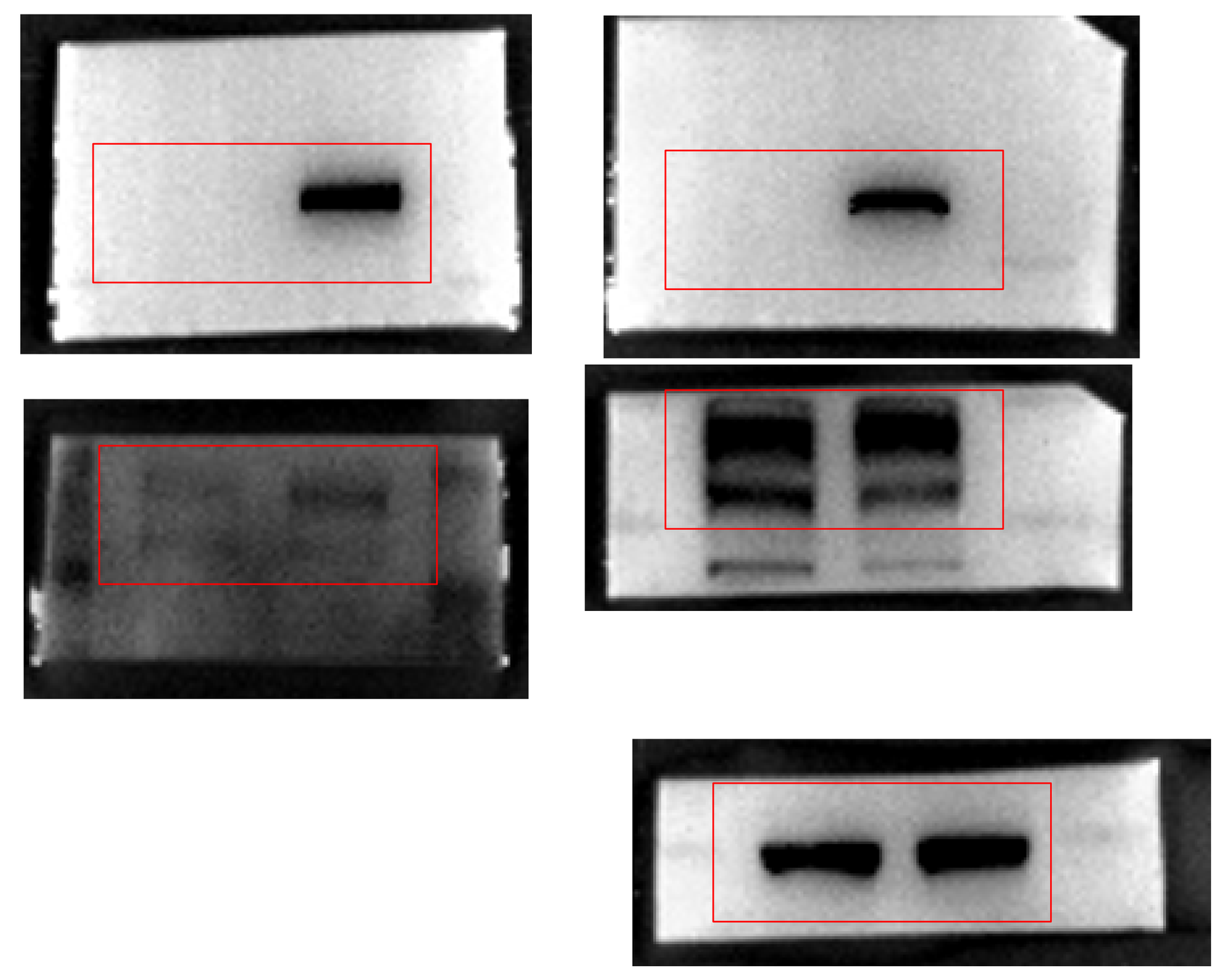

Full unedited blot
for Figure 3G

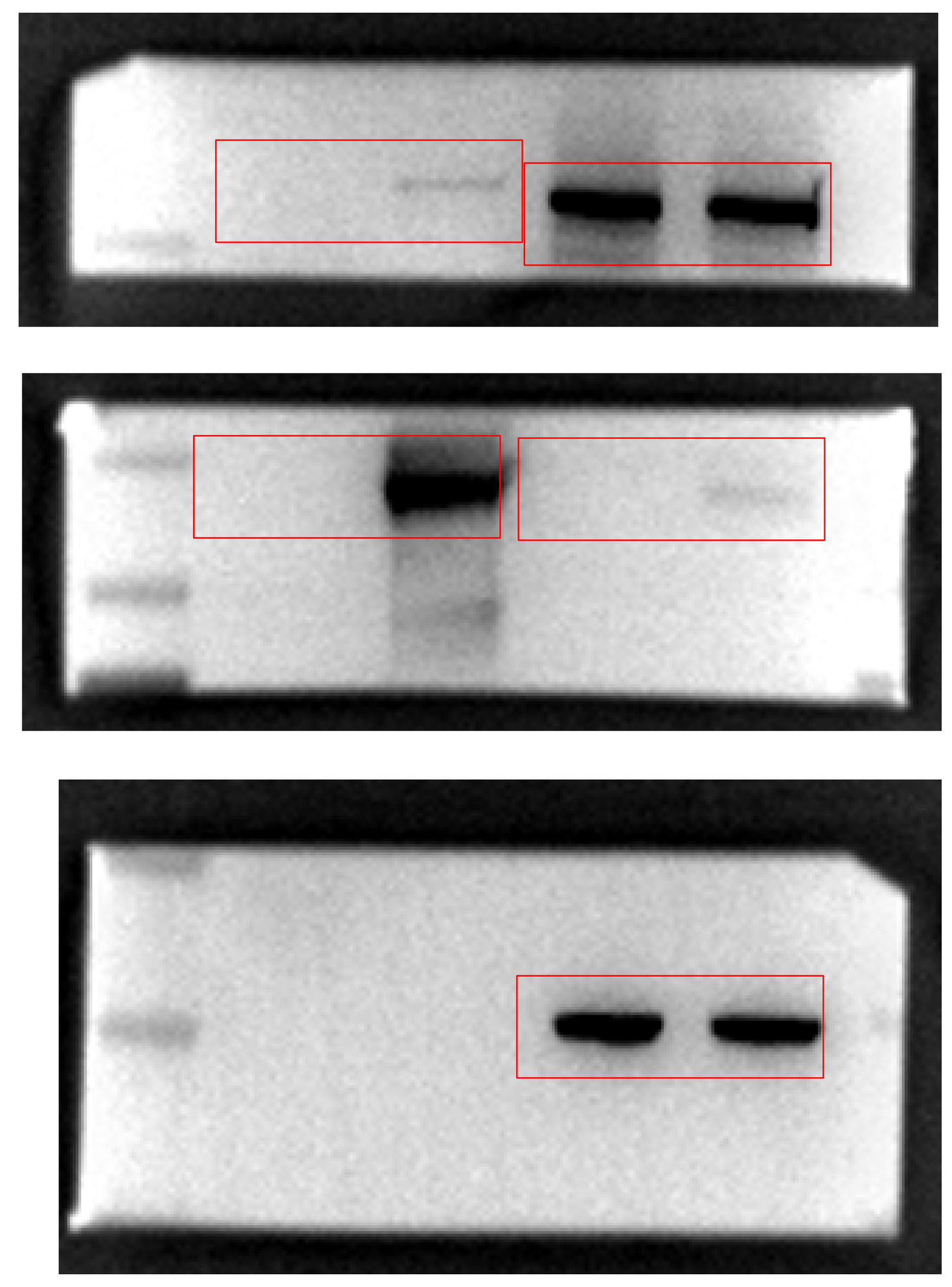

Full unedited gel
for Figure 3H

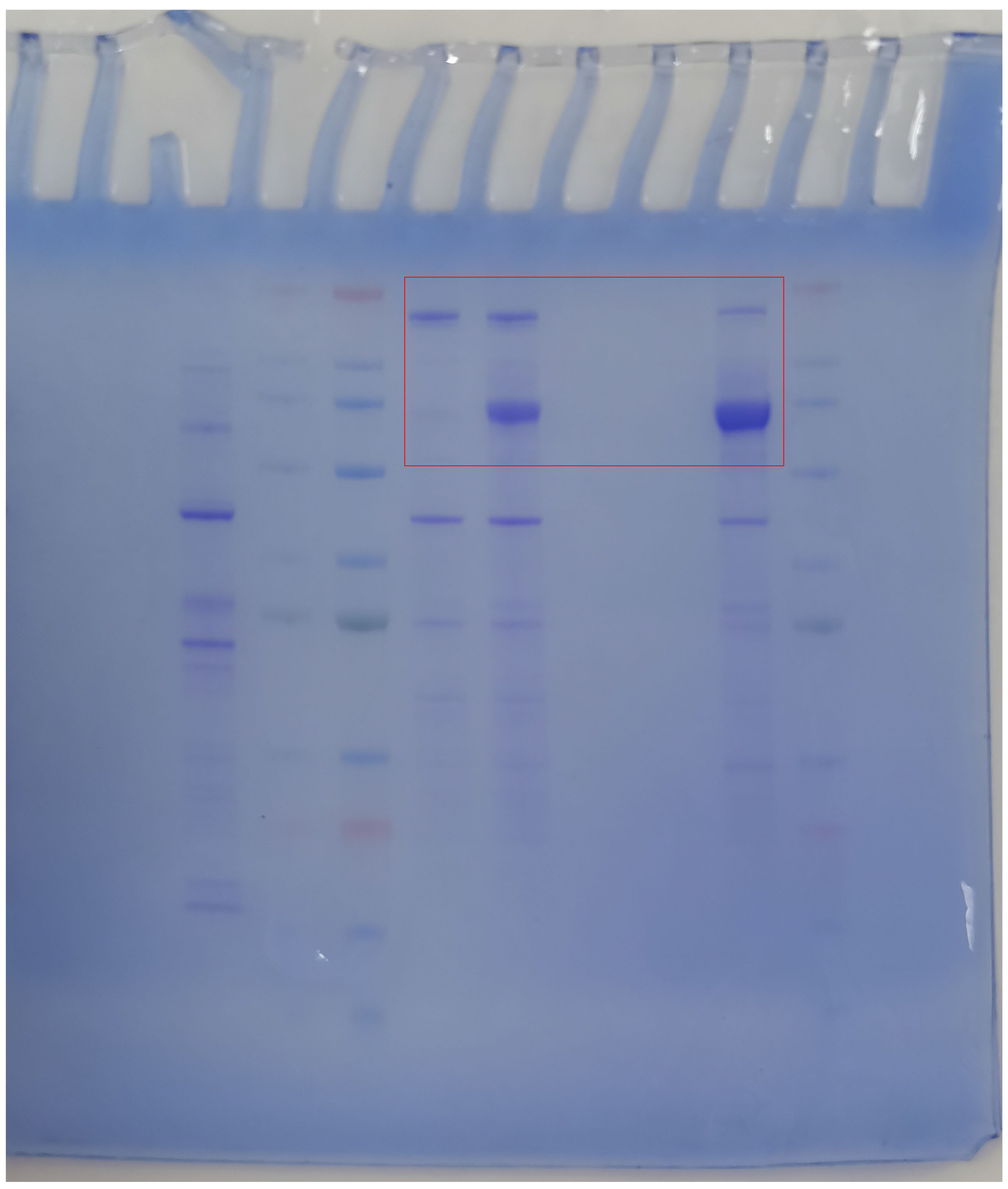

Full unedited blot
for Figure 3I

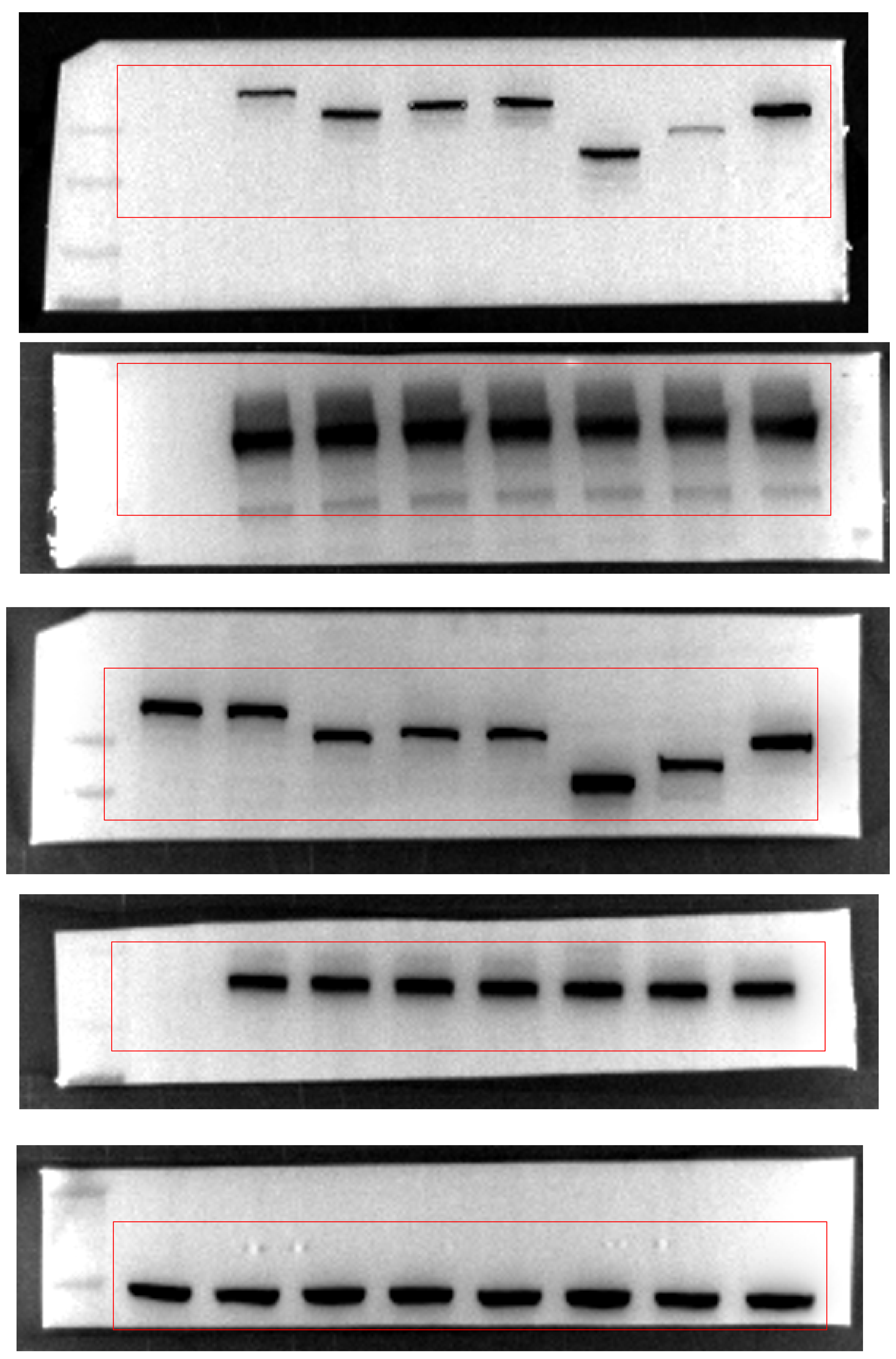

Full unedited blot
for Figure 3K

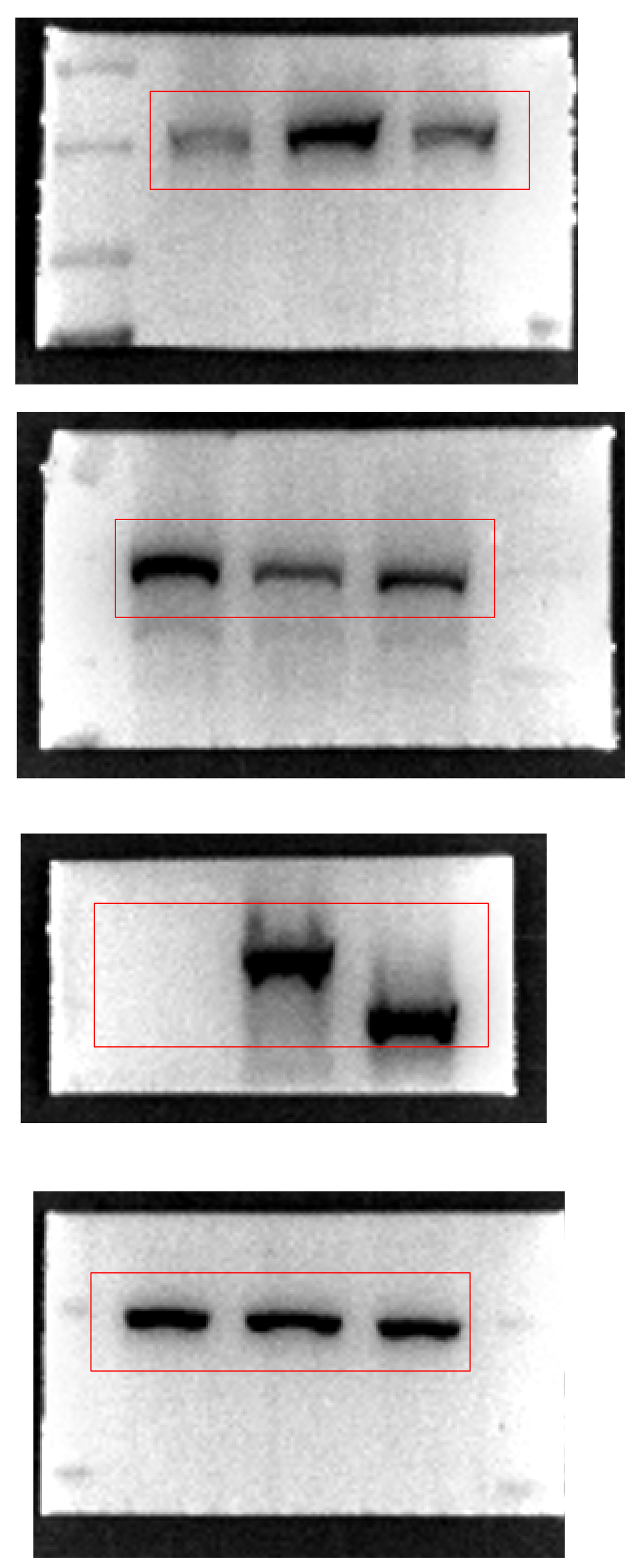

Full unedited blot
for Figure 3L

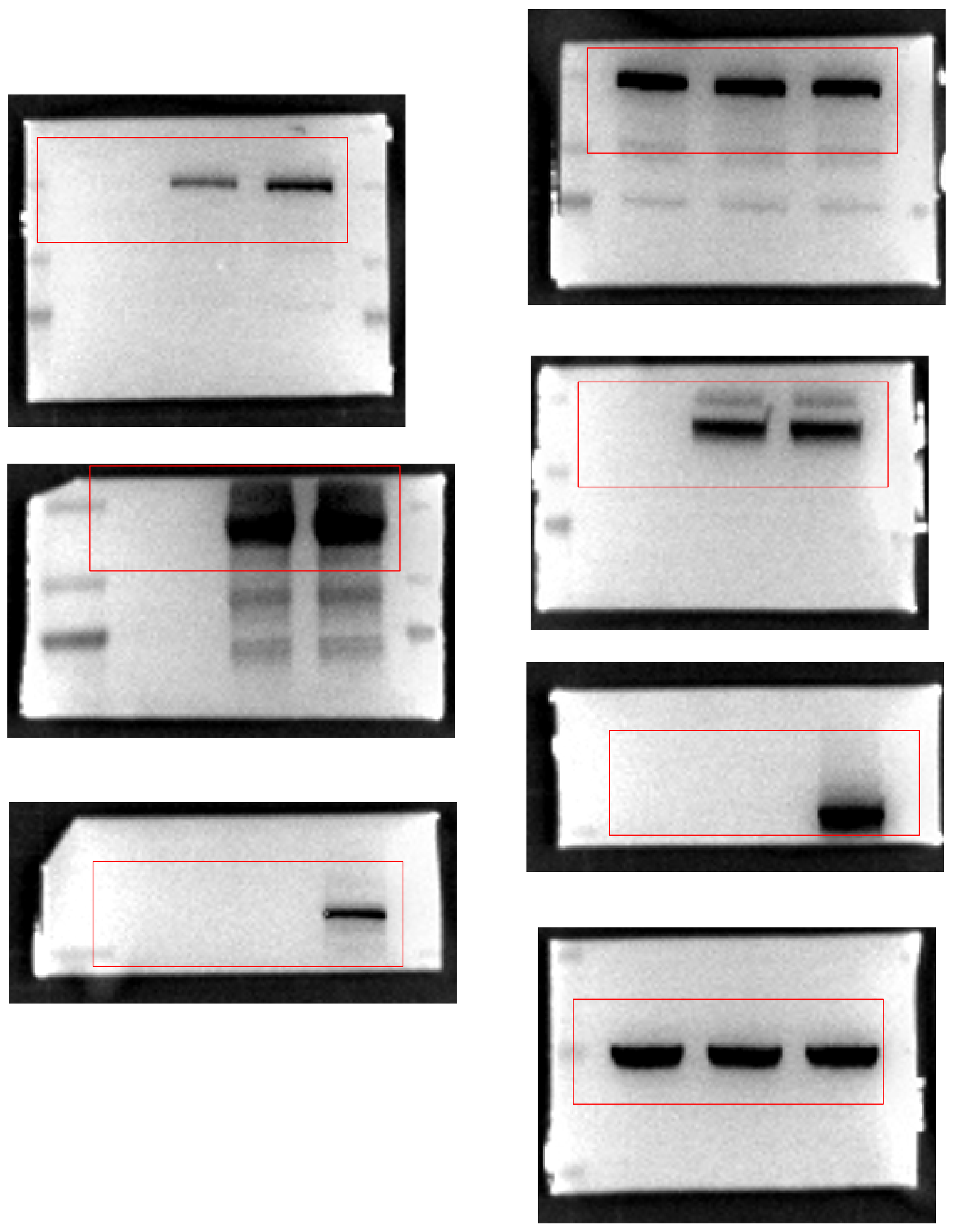

Full unedited blot
for Figure 3M

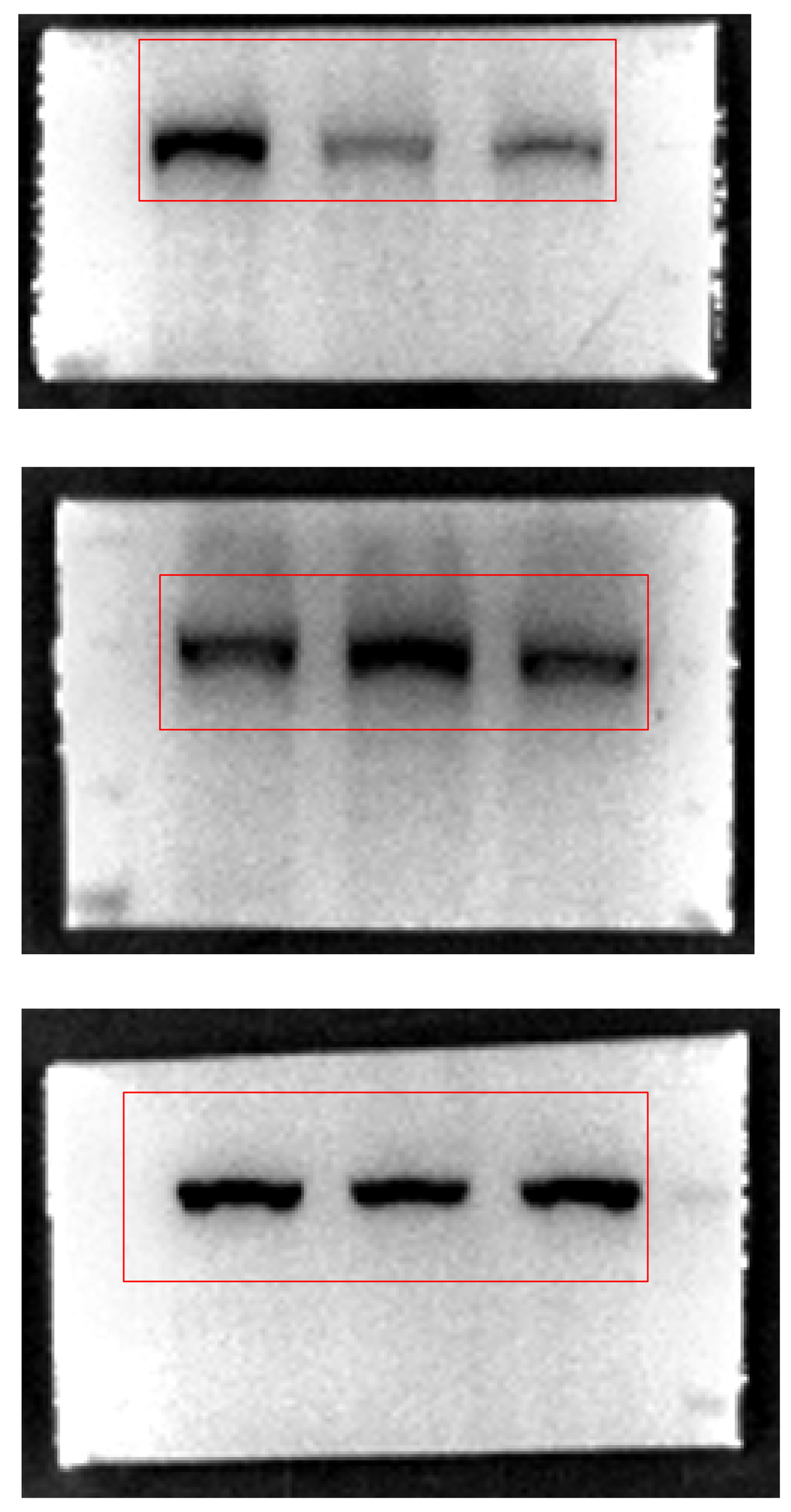

Full unedited blot
for Figure 3O

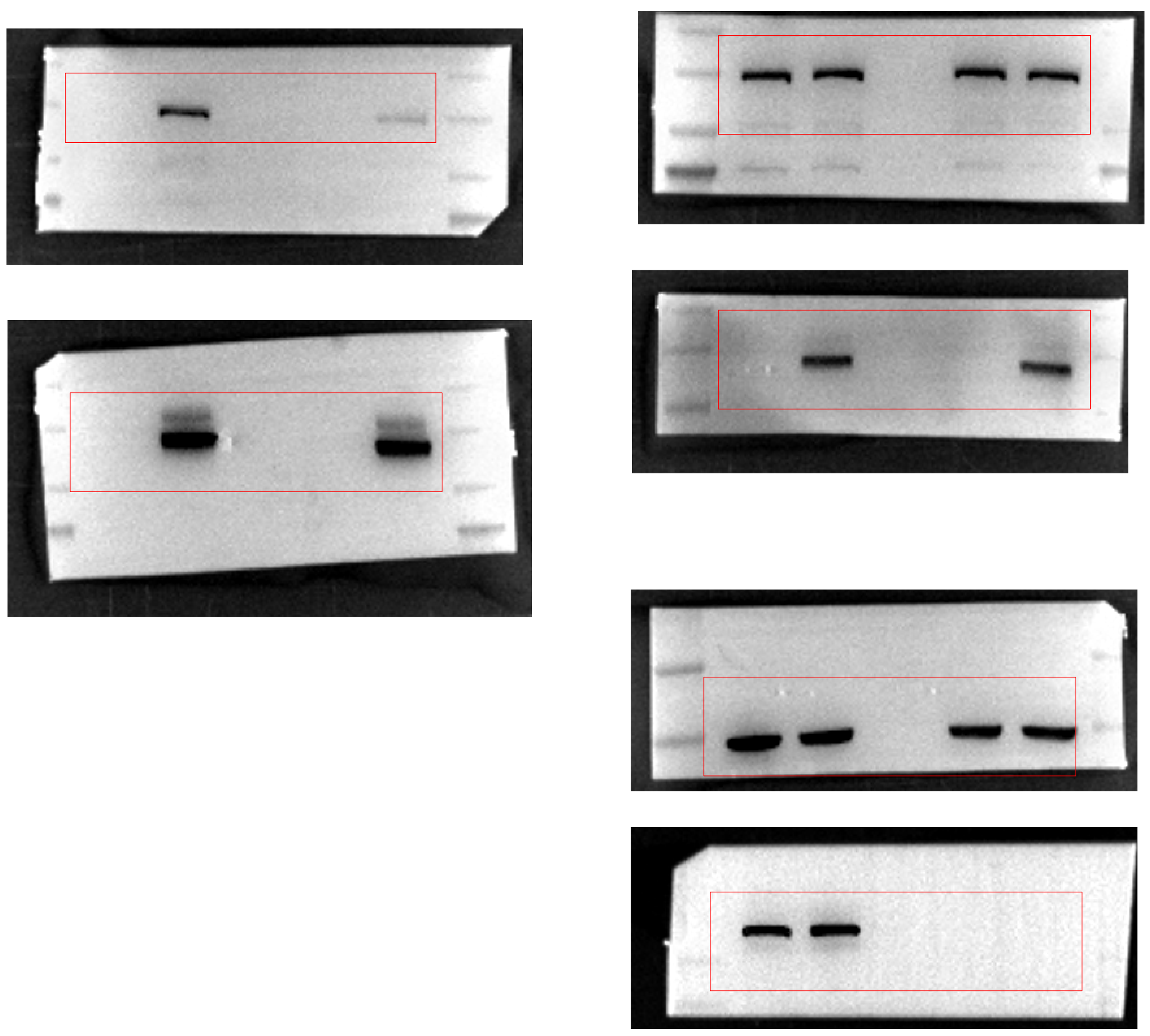

Full unedited blot
for Figure 4A

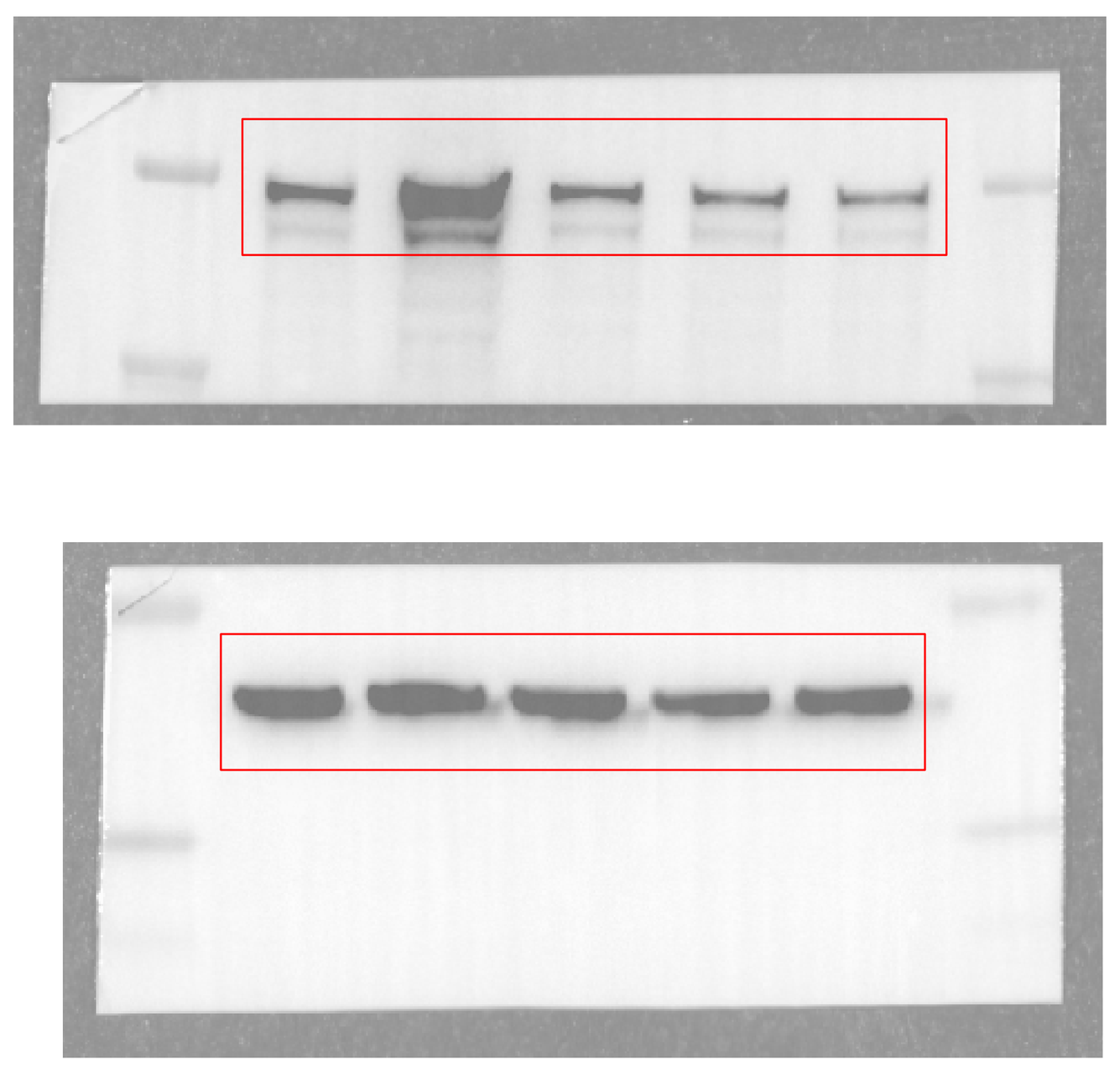

Full unedited blot
for Figure 4B

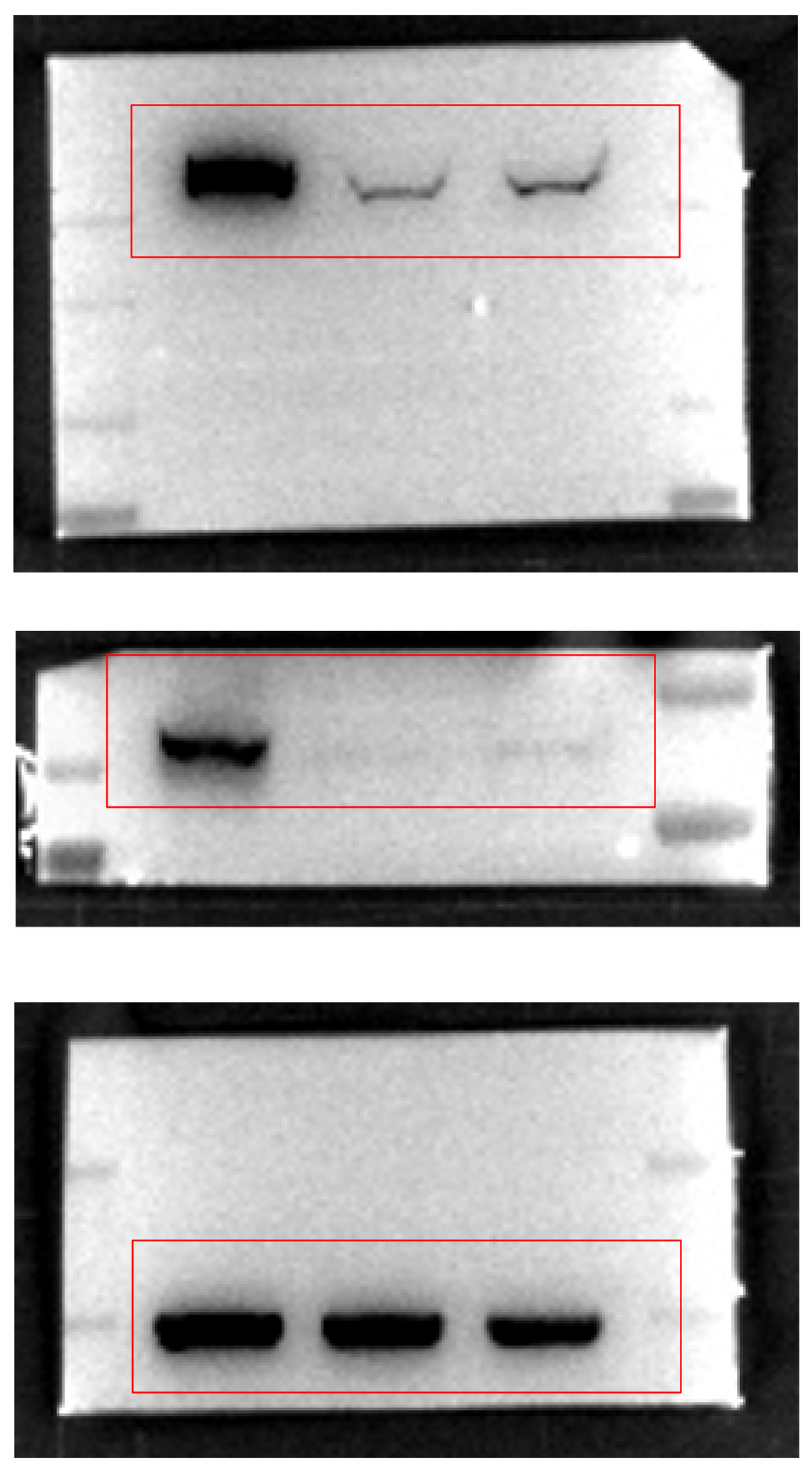

Full unedited blot
for Figure 4C

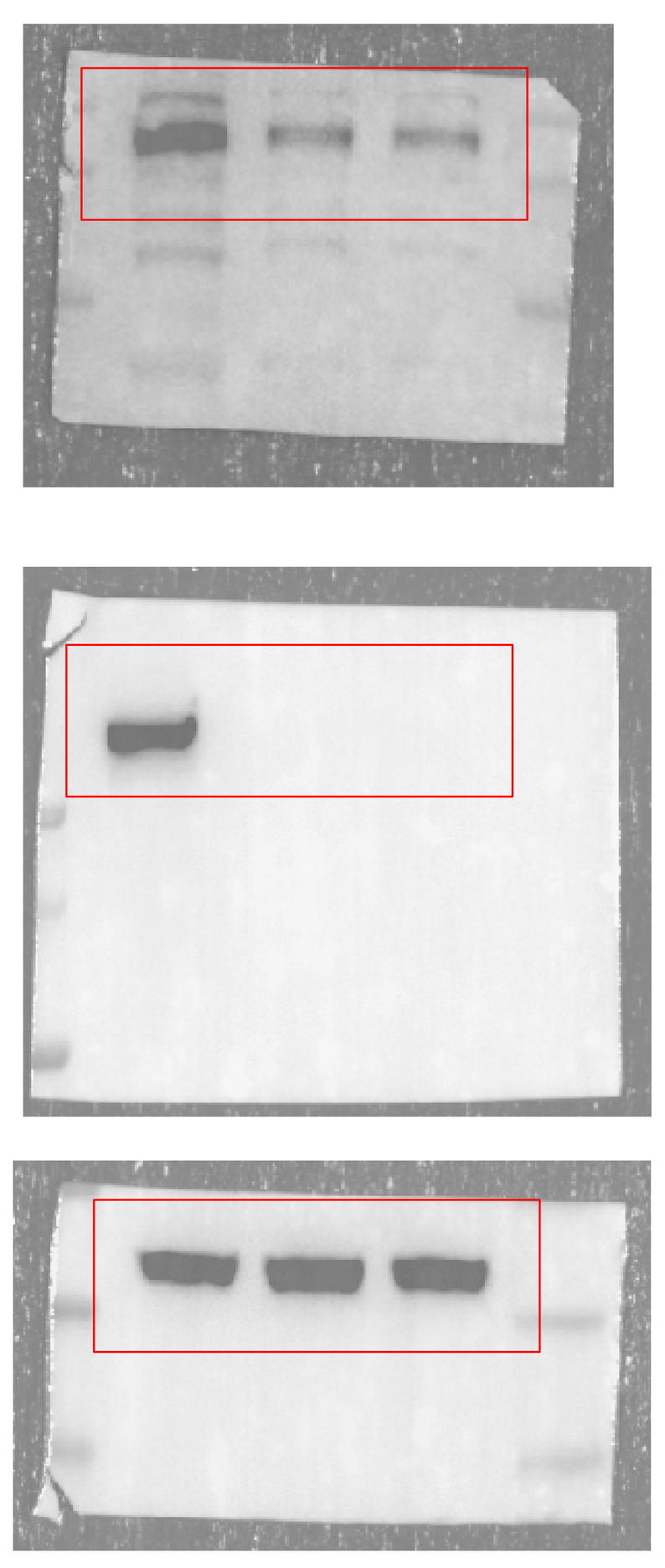

Full unedited blot
for Figure 5A

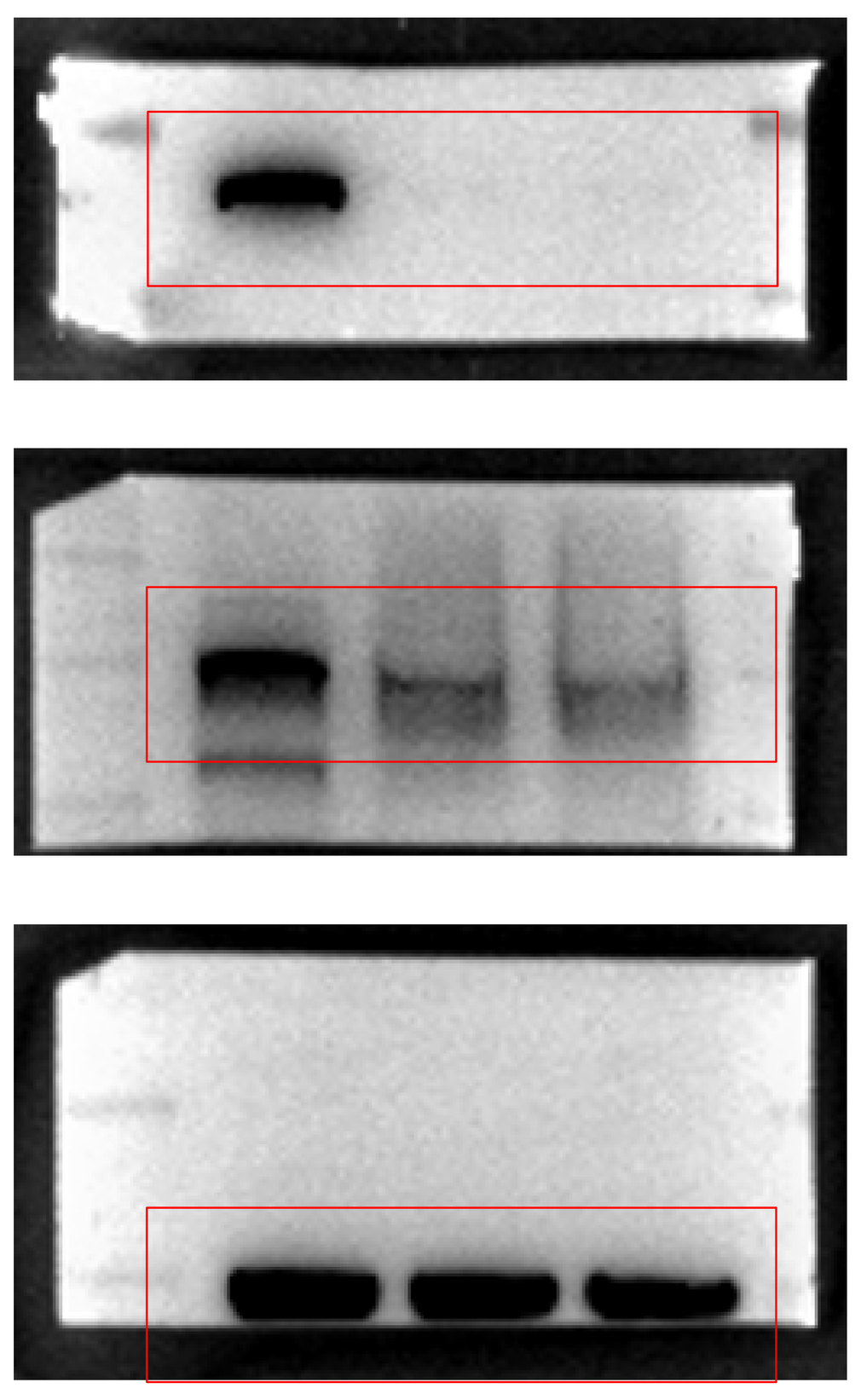

Full unedited blot
for Figure 5D

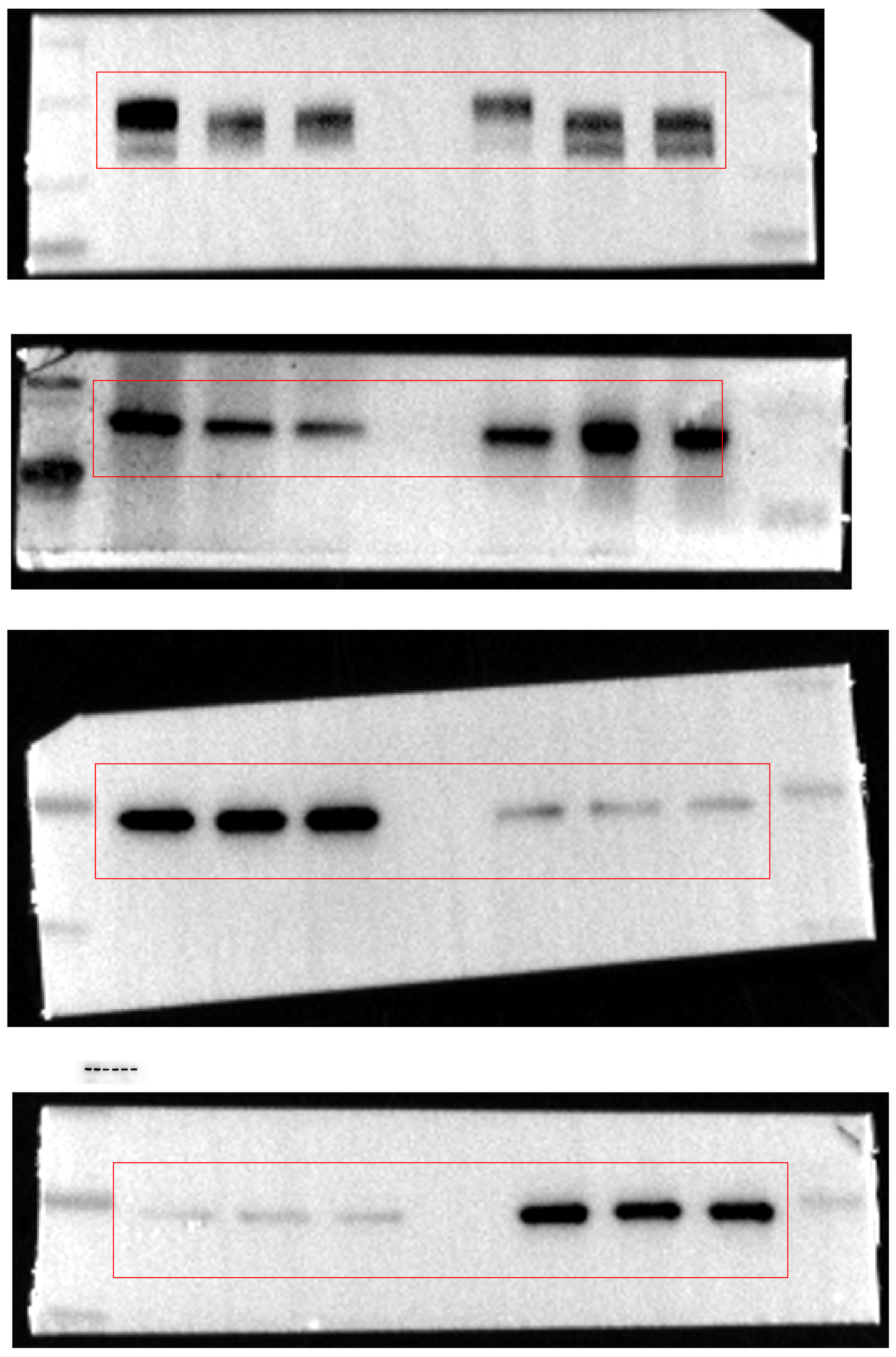

Full unedited blot
for Figure 5G

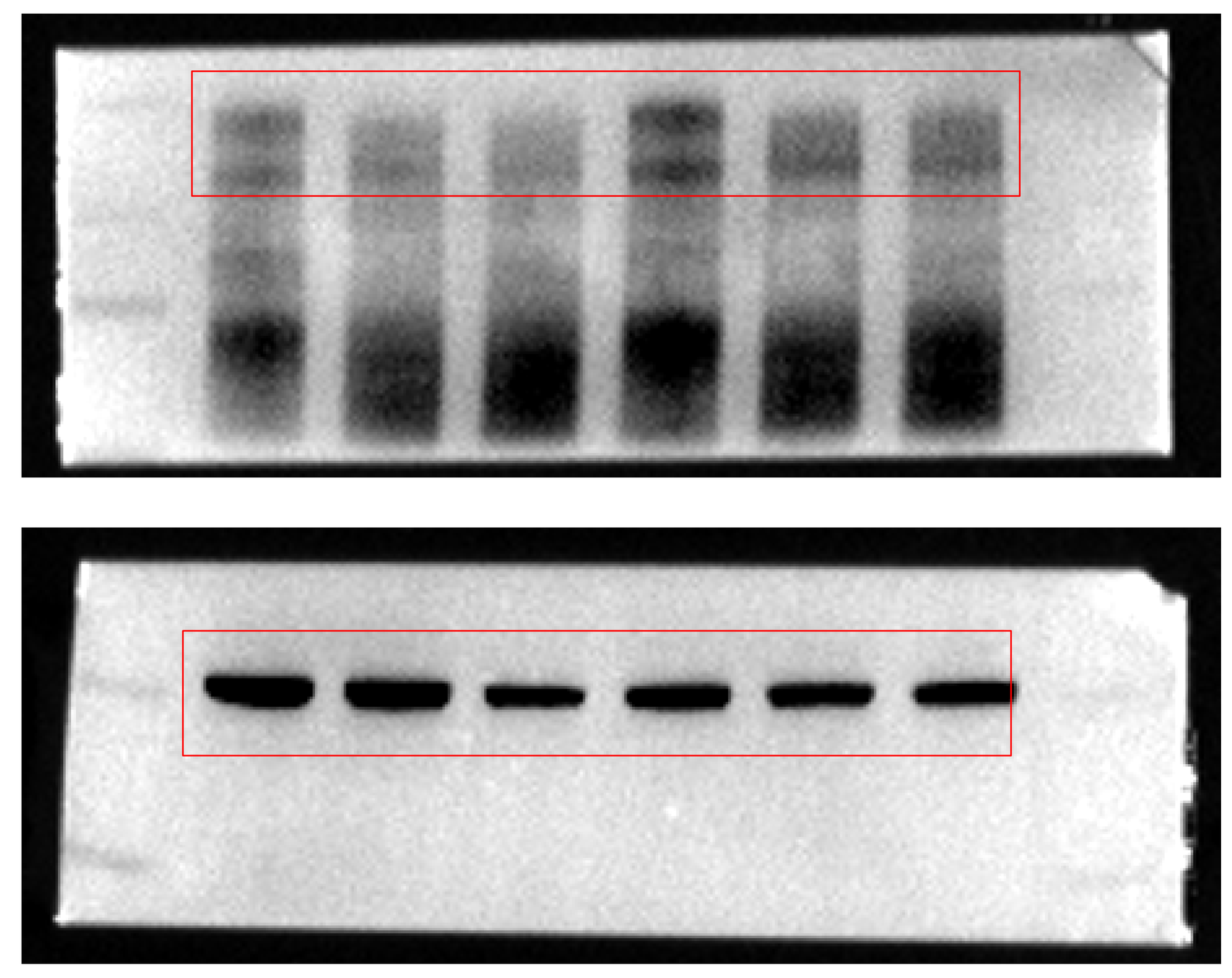

Full unedited blot
for Figure 5H

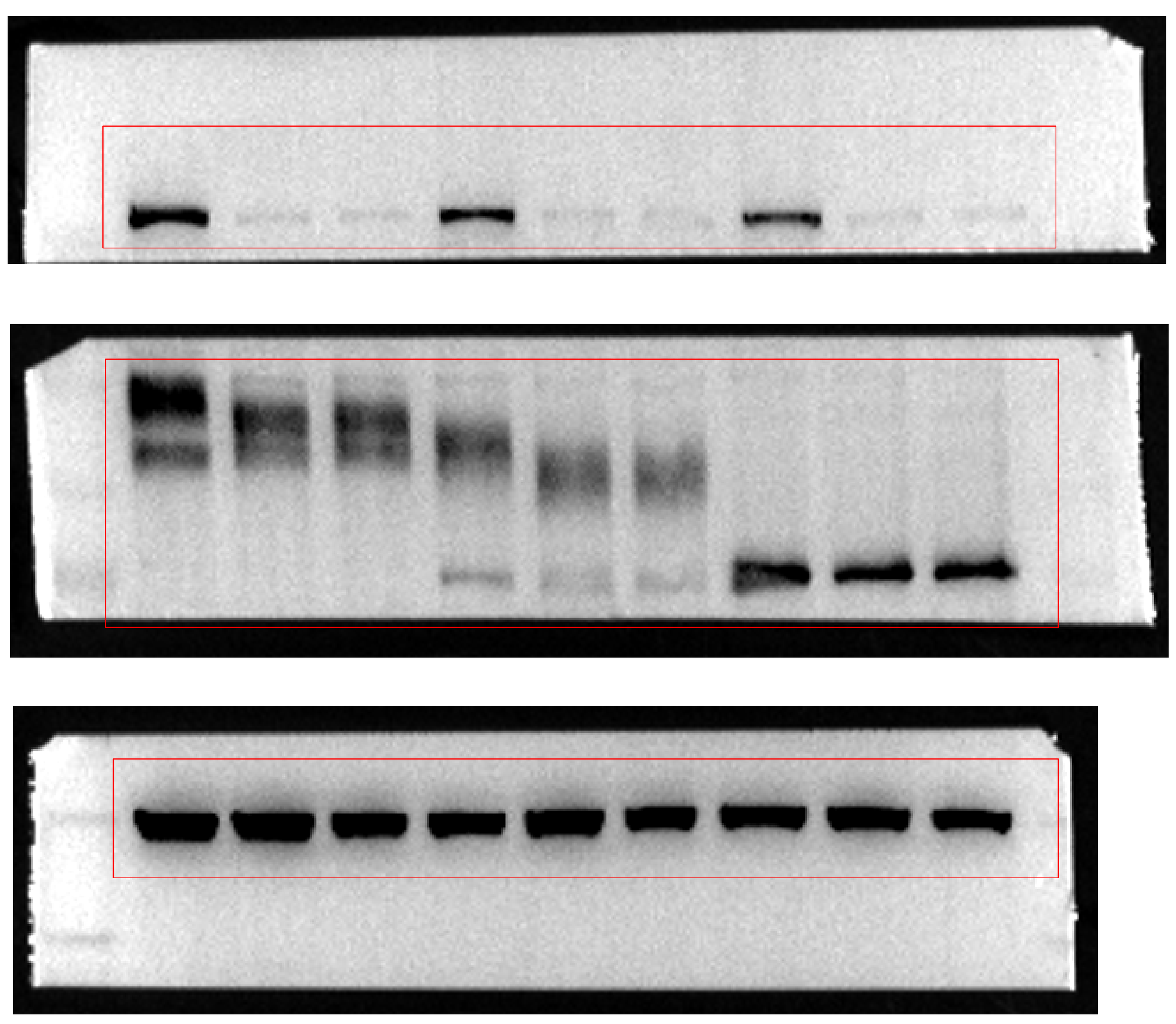

Full unedited blot
for Figure 5I

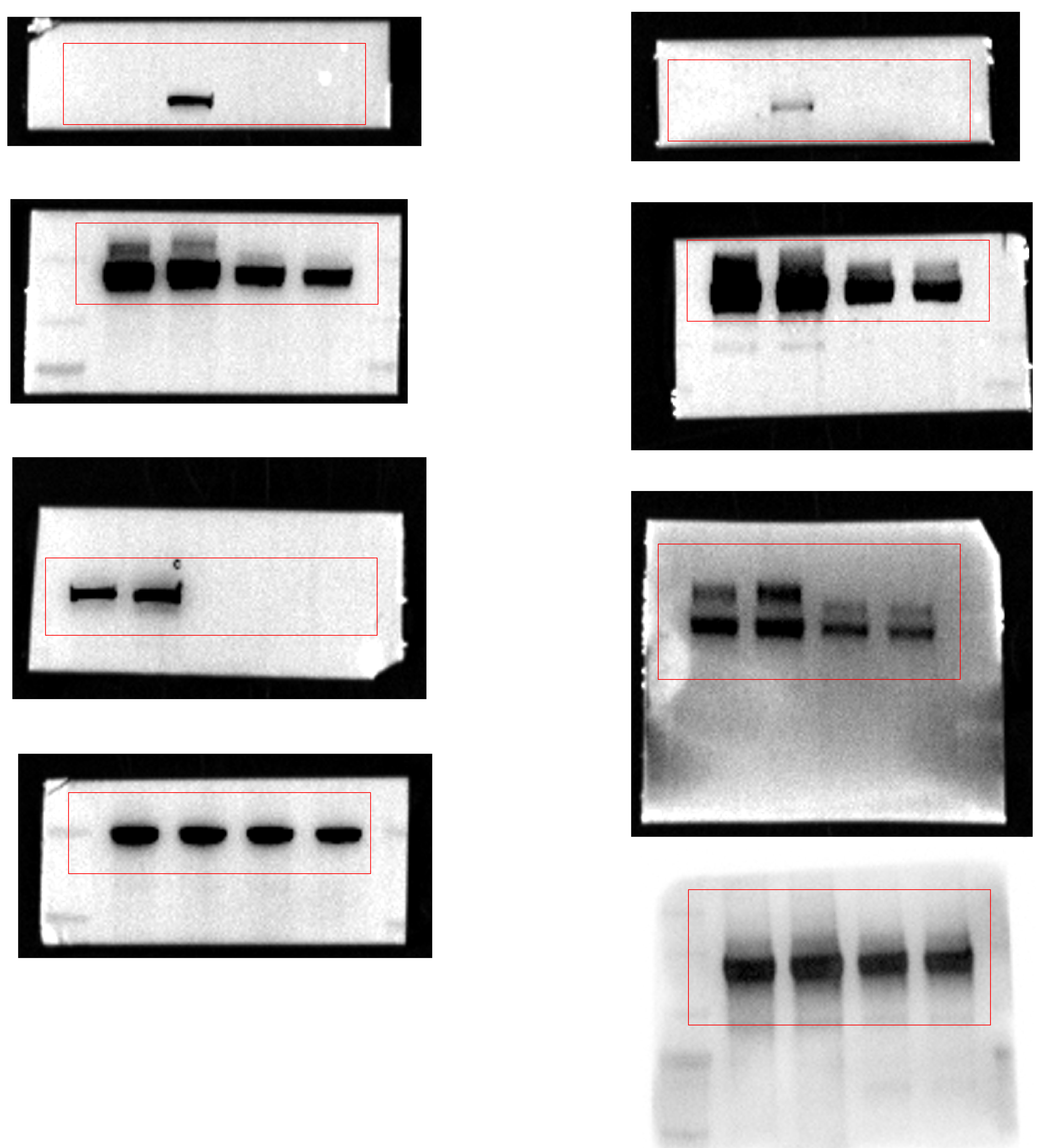

Full unedited blot
for Figure 5J

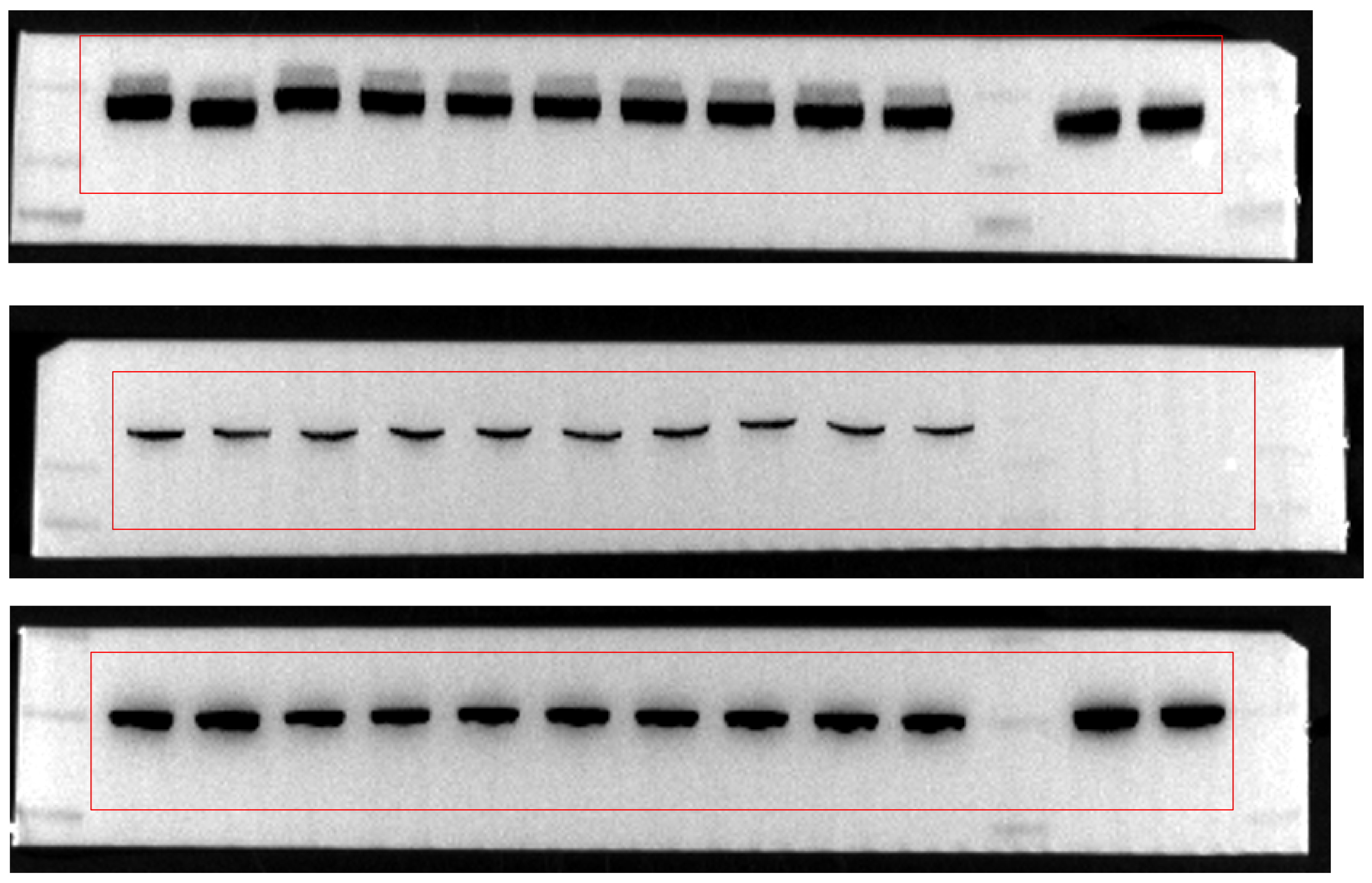

Full unedited blot
for Figure 5K

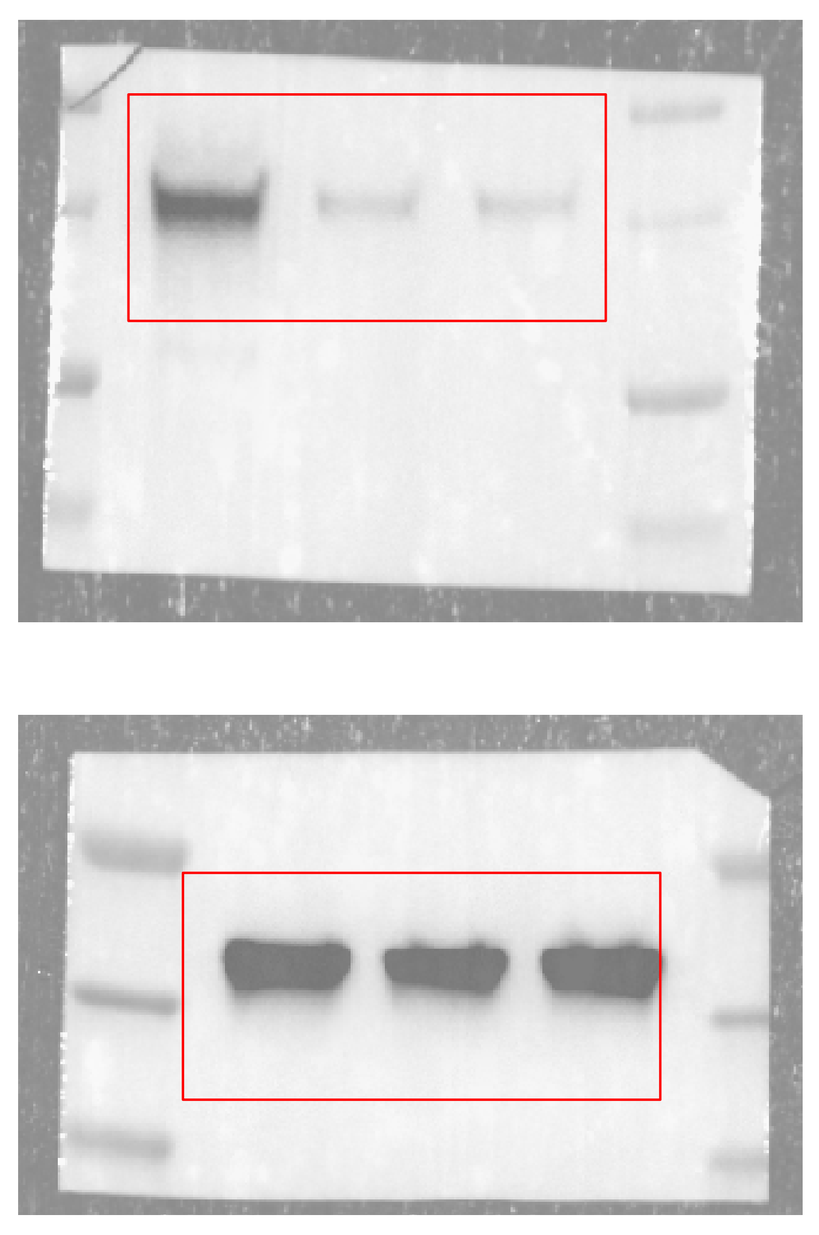

Full unedited blot
for Figure 5N

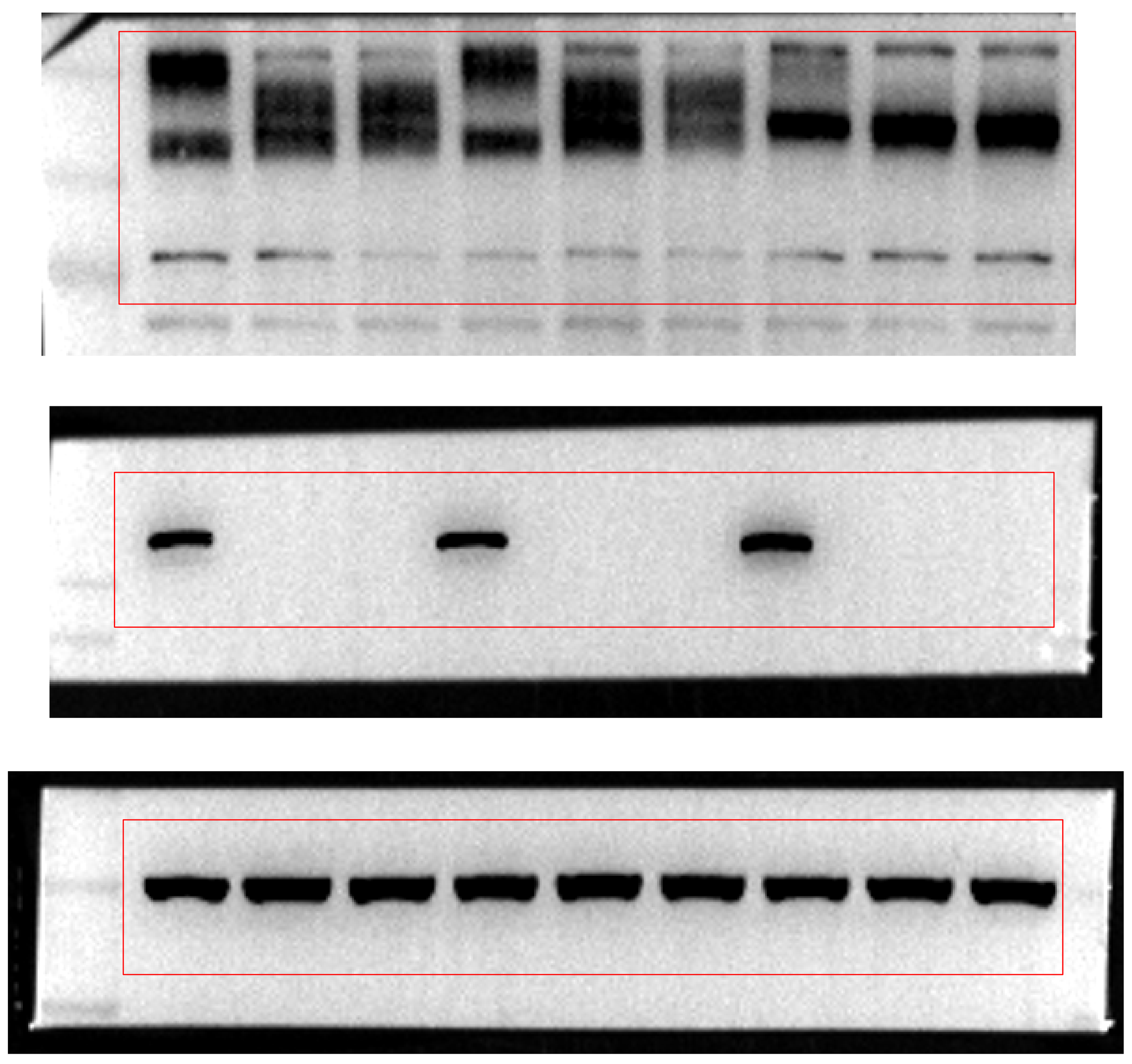

Full unedited blot
for Figure 5O

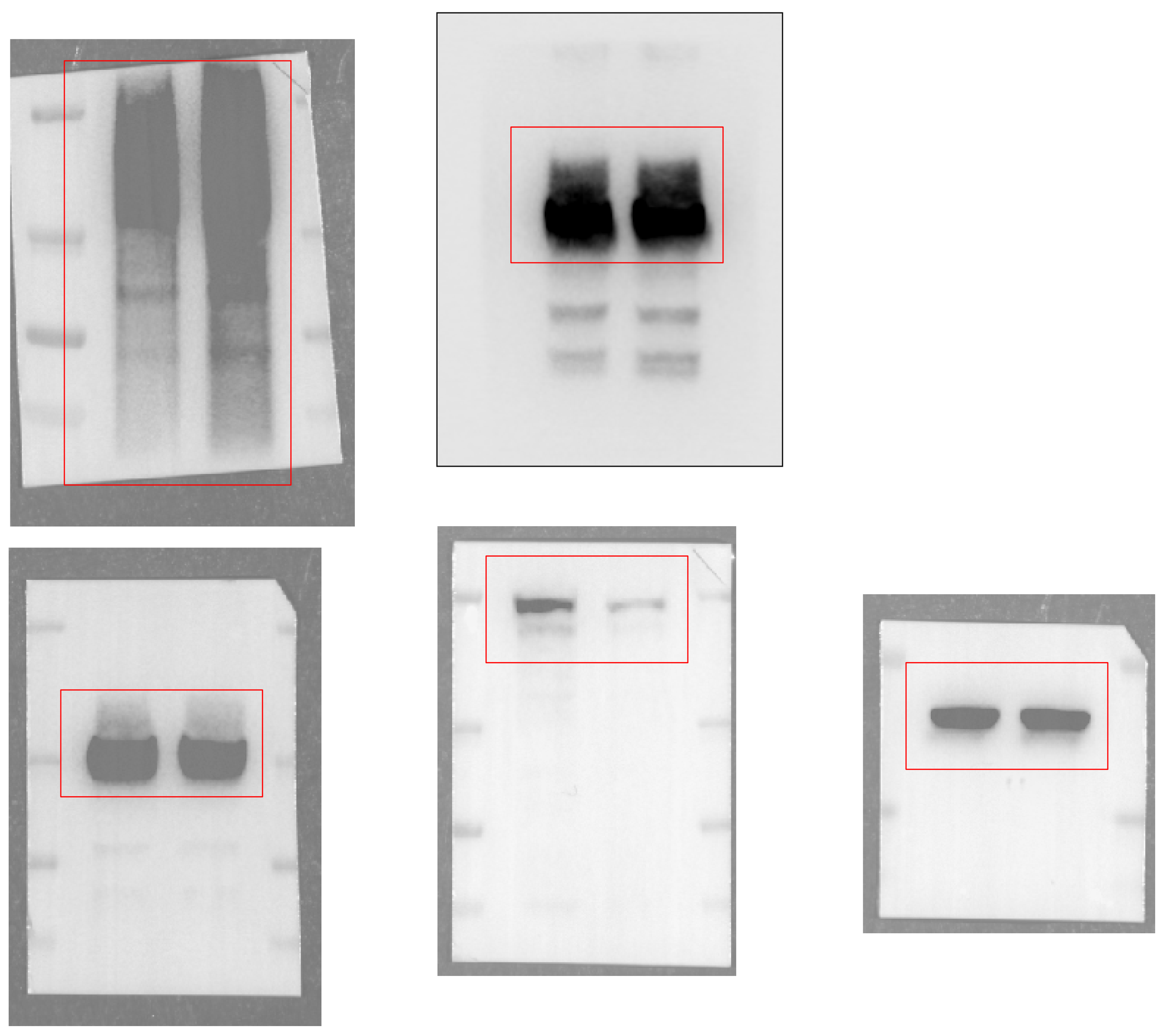

Full unedited blot for
Supplemental Figure S1J

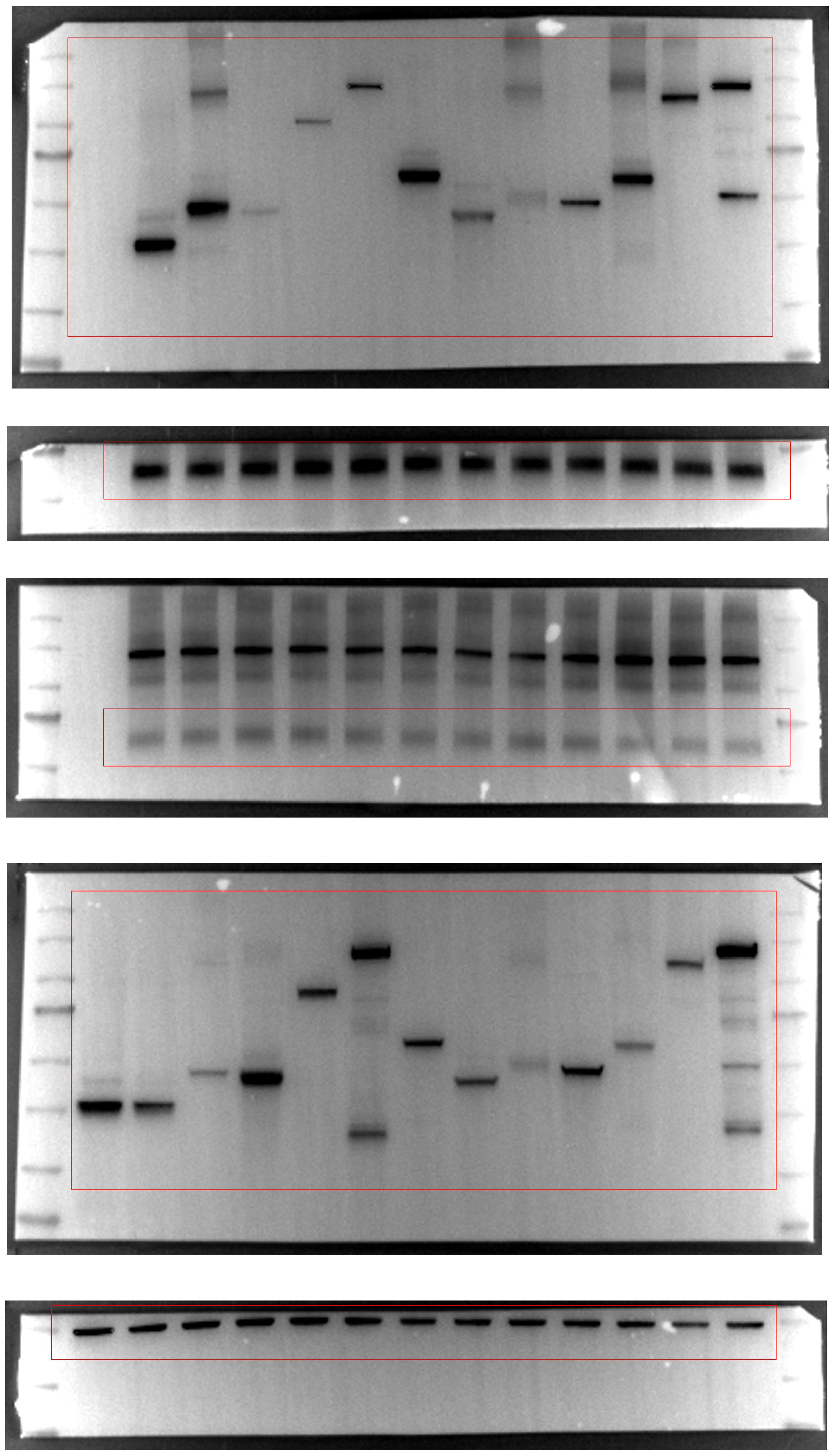

Full unedited blot for Supplemental Figure S5A

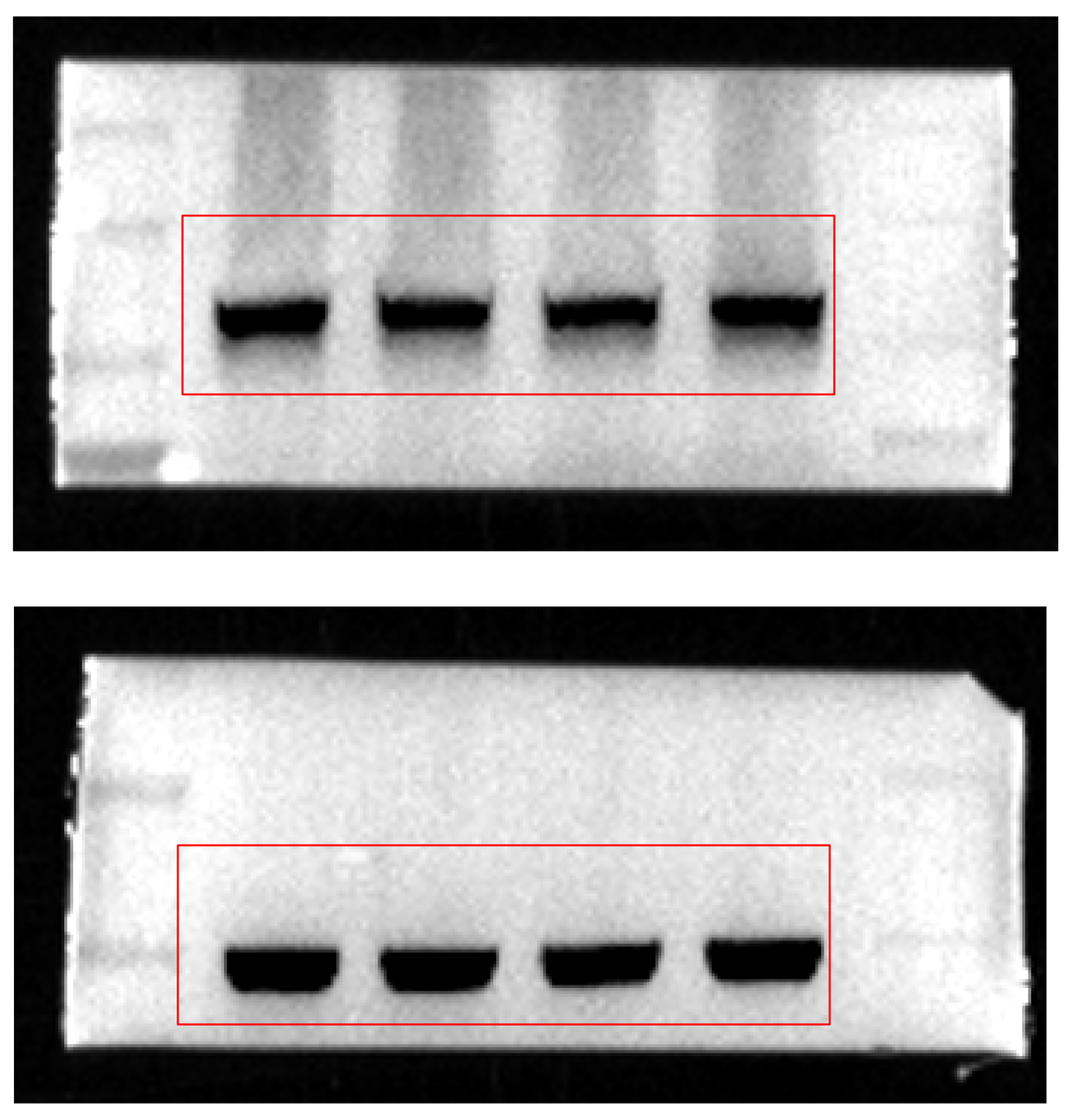

Full unedited blot for Supplemental Figure S5B

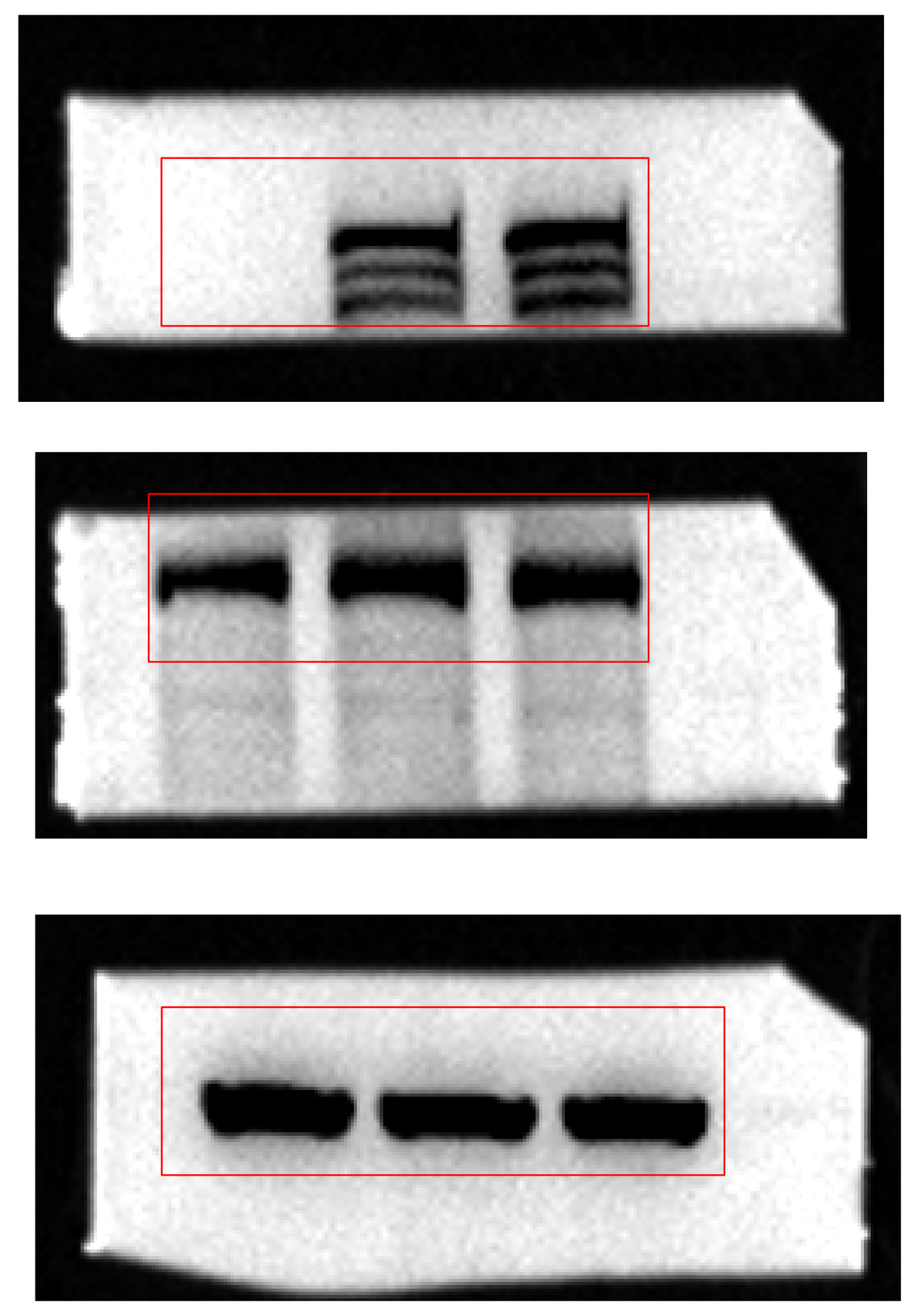

Full unedited blot for Supplemental Figure S5O

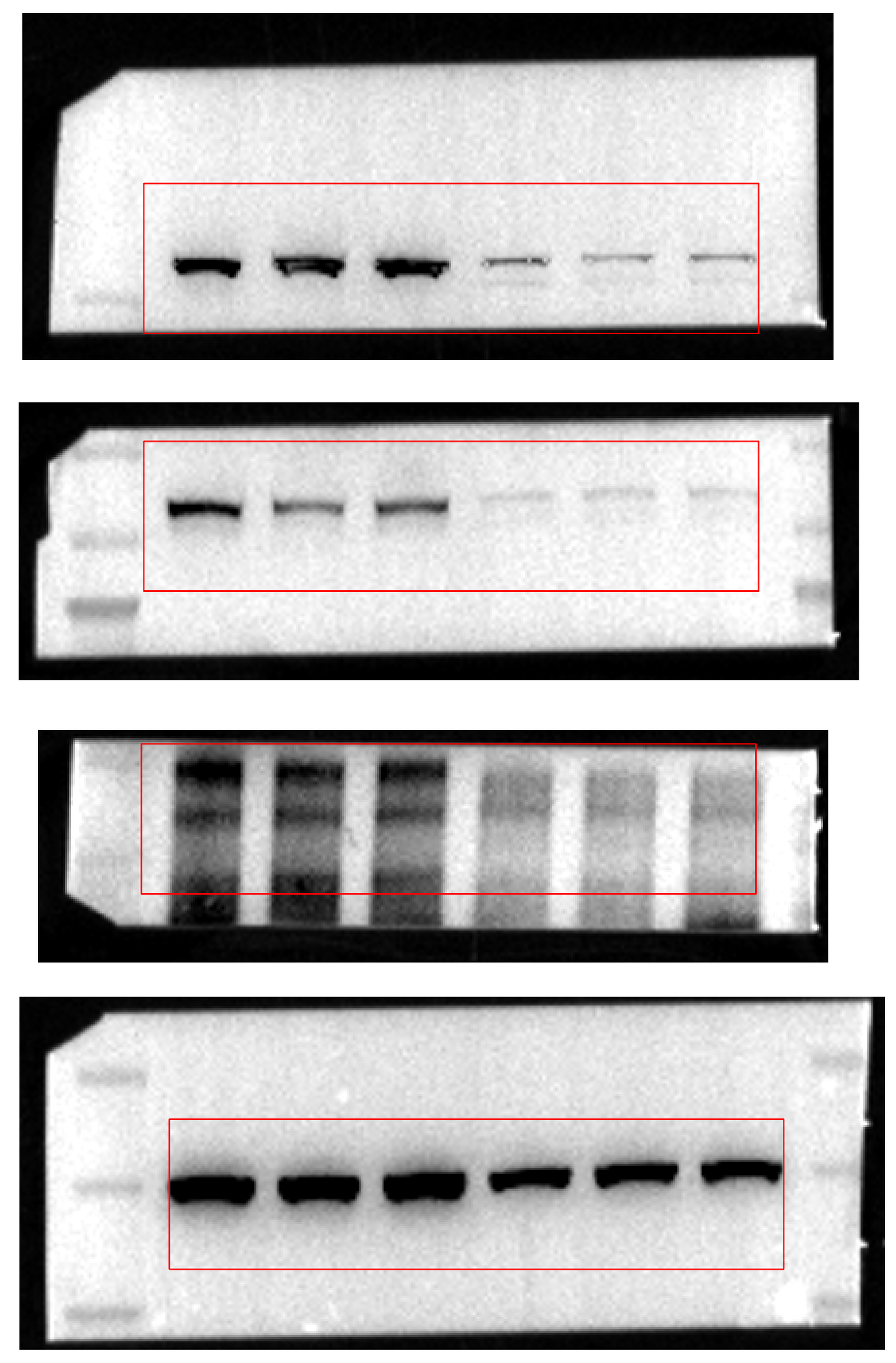

Full unedited blot for
Supplemental Figure S6A

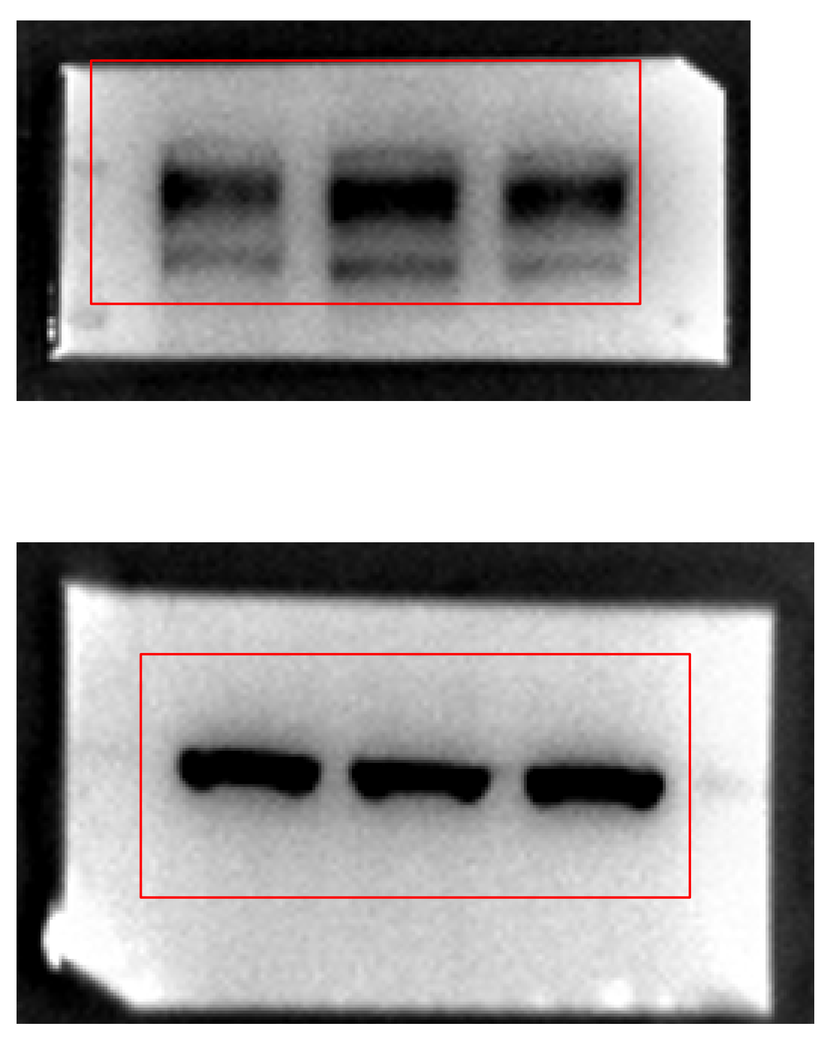

Full unedited blot for
Supplemental Figure S6B

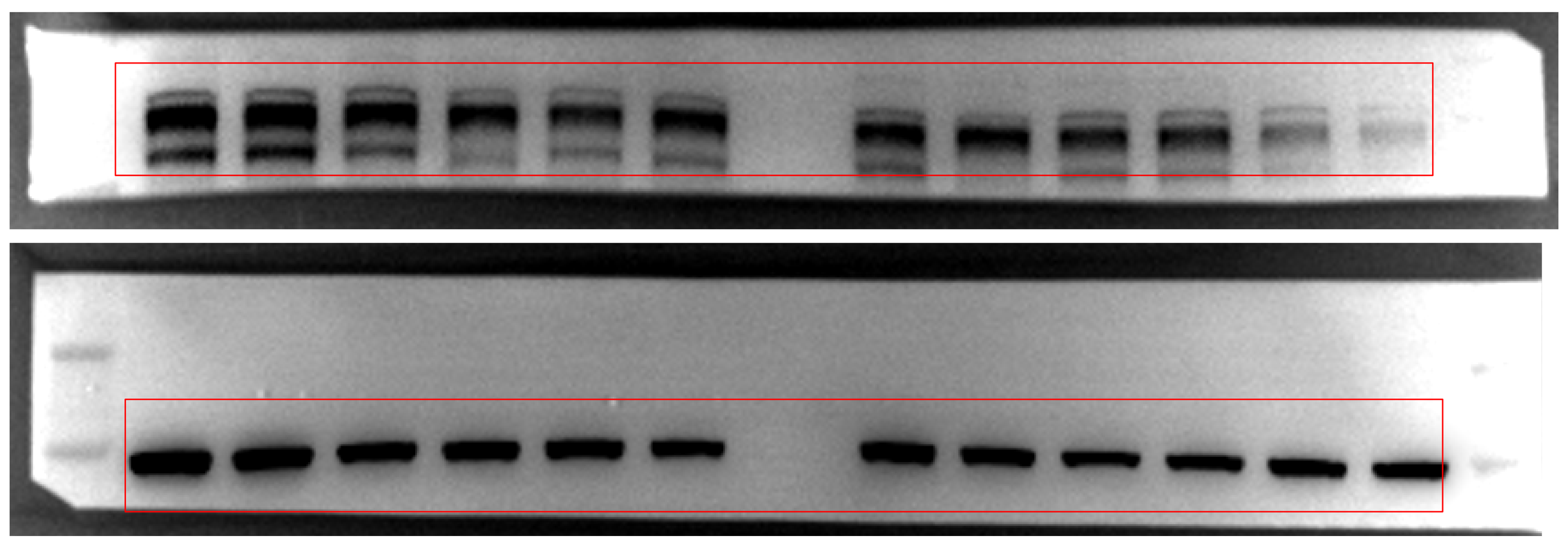

Full unedited blot for
Supplemental Figure S6F

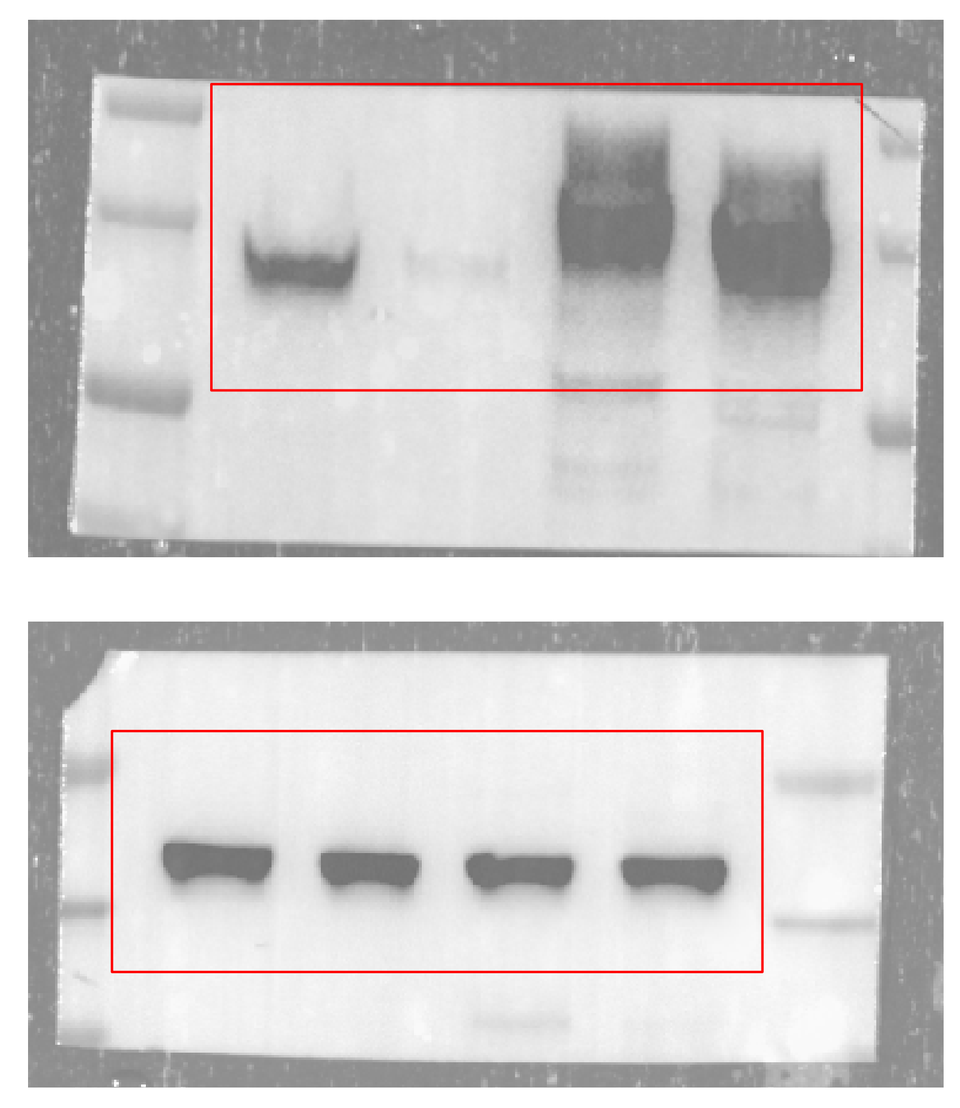

Full unedited blot for
Supplemental Figure S6L

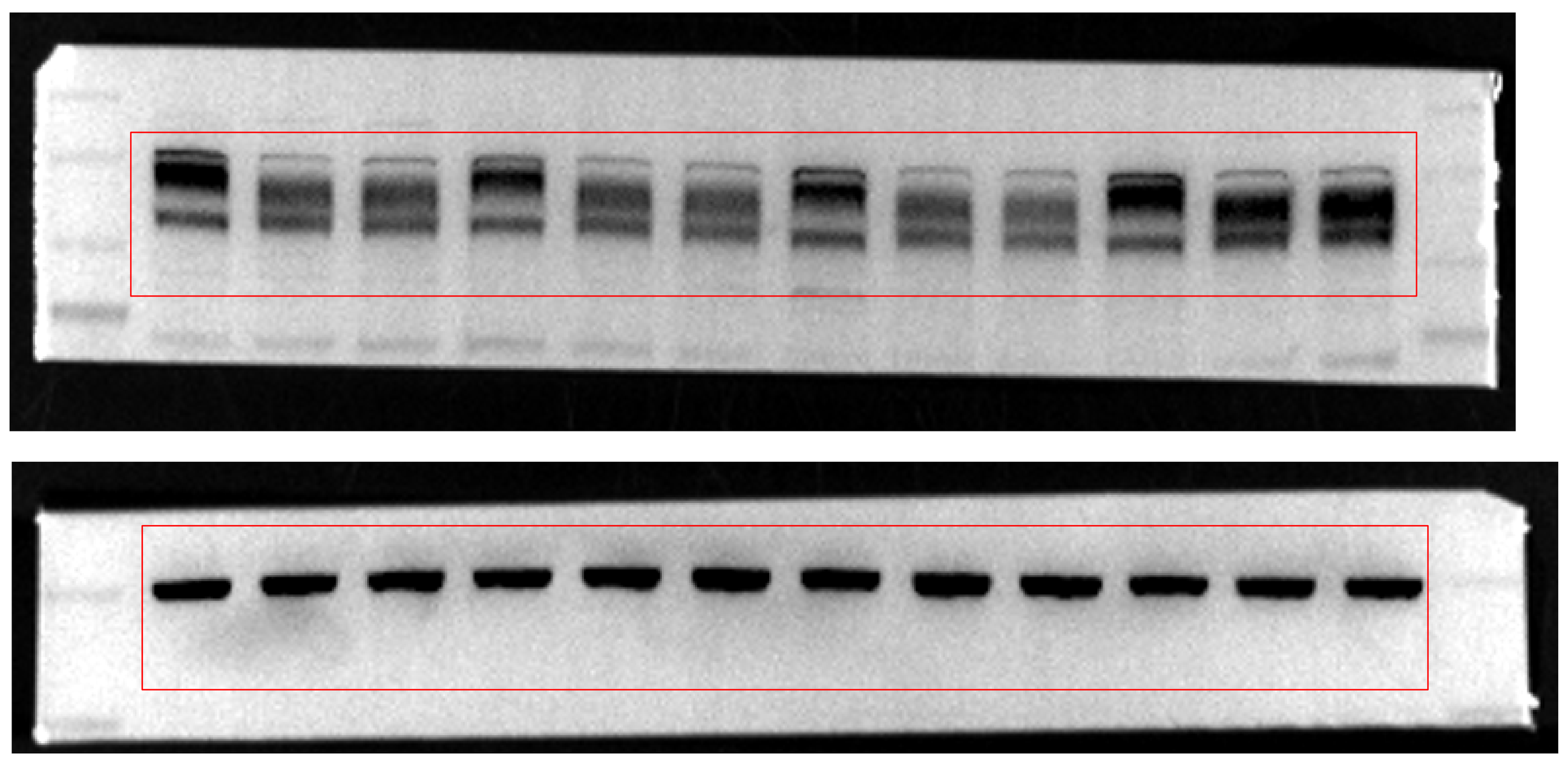

Full unedited blot for
Supplemental Figure S6M

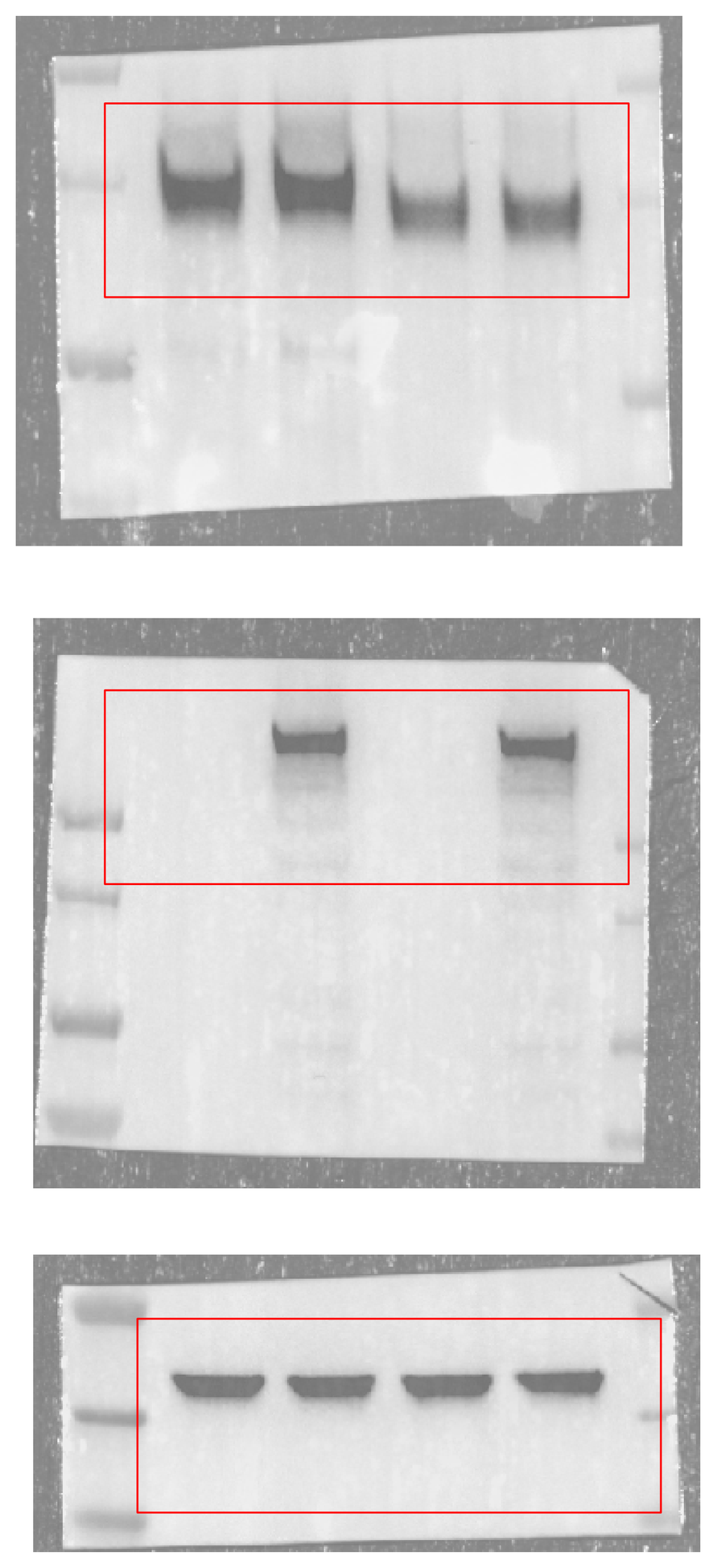

Full unedited blot for
Supplemental Figure S6O

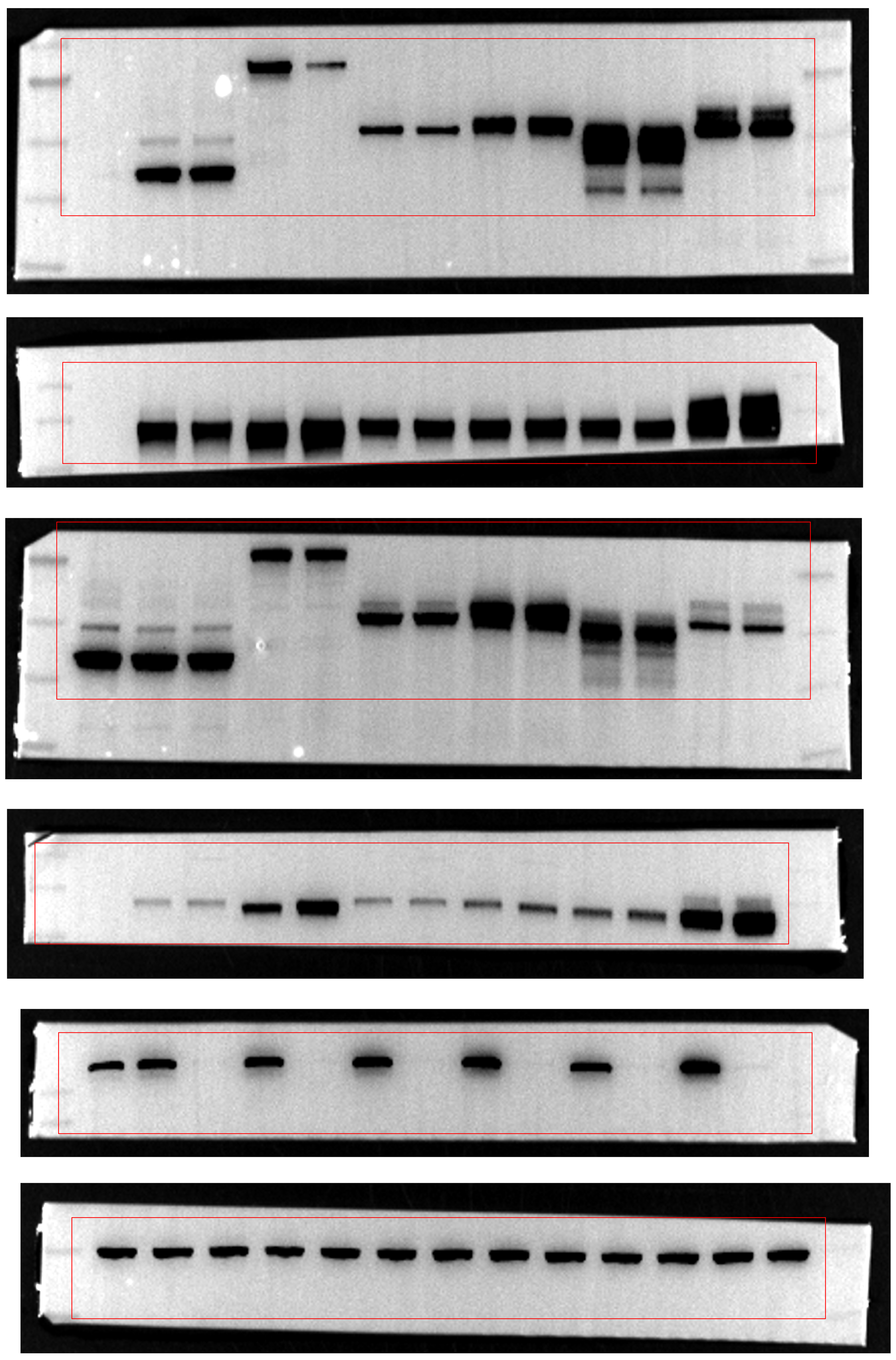

Full unedited blot for
Supplemental Figure S7F

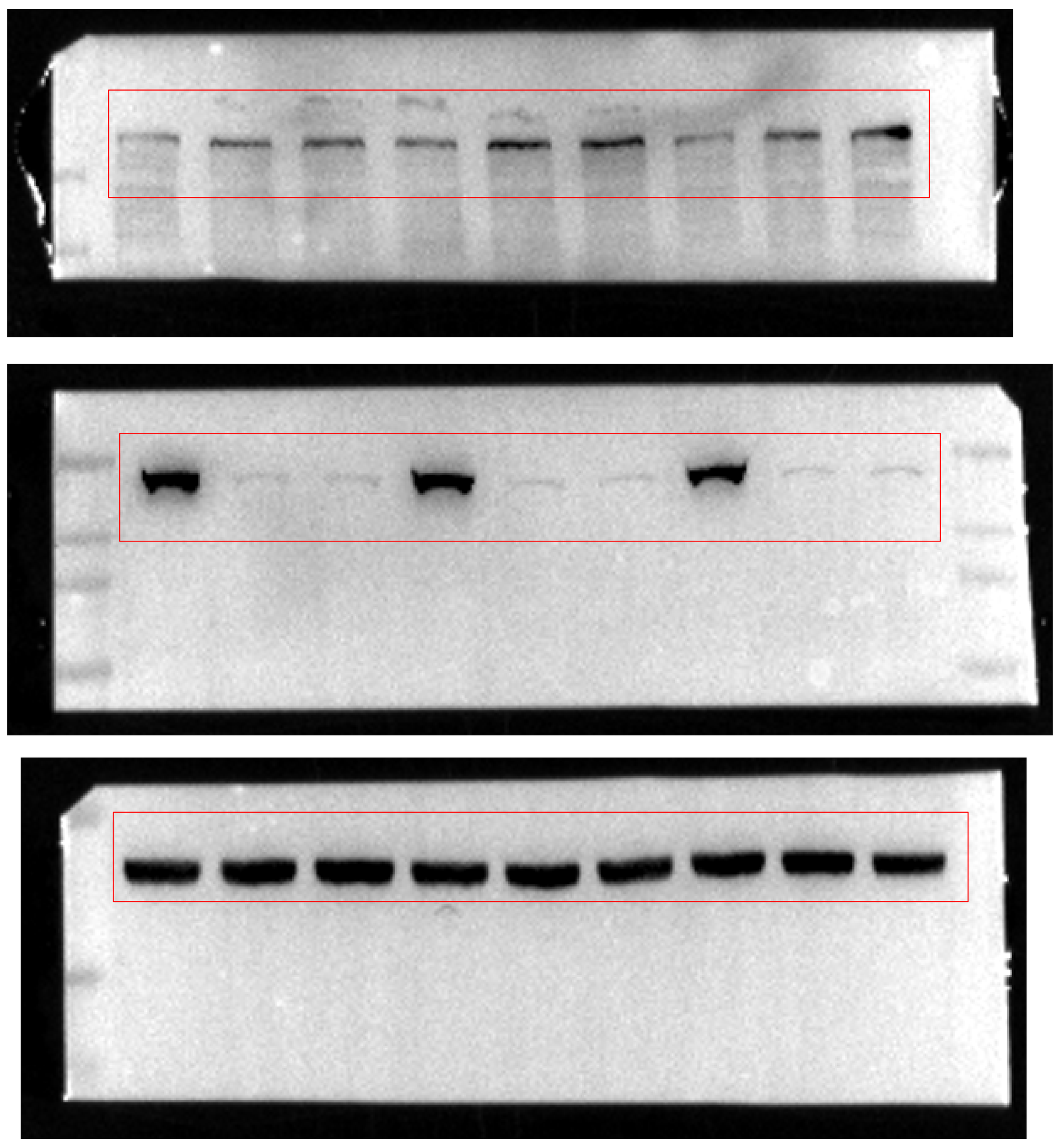

Full unedited blot for
Supplemental Figure S7G

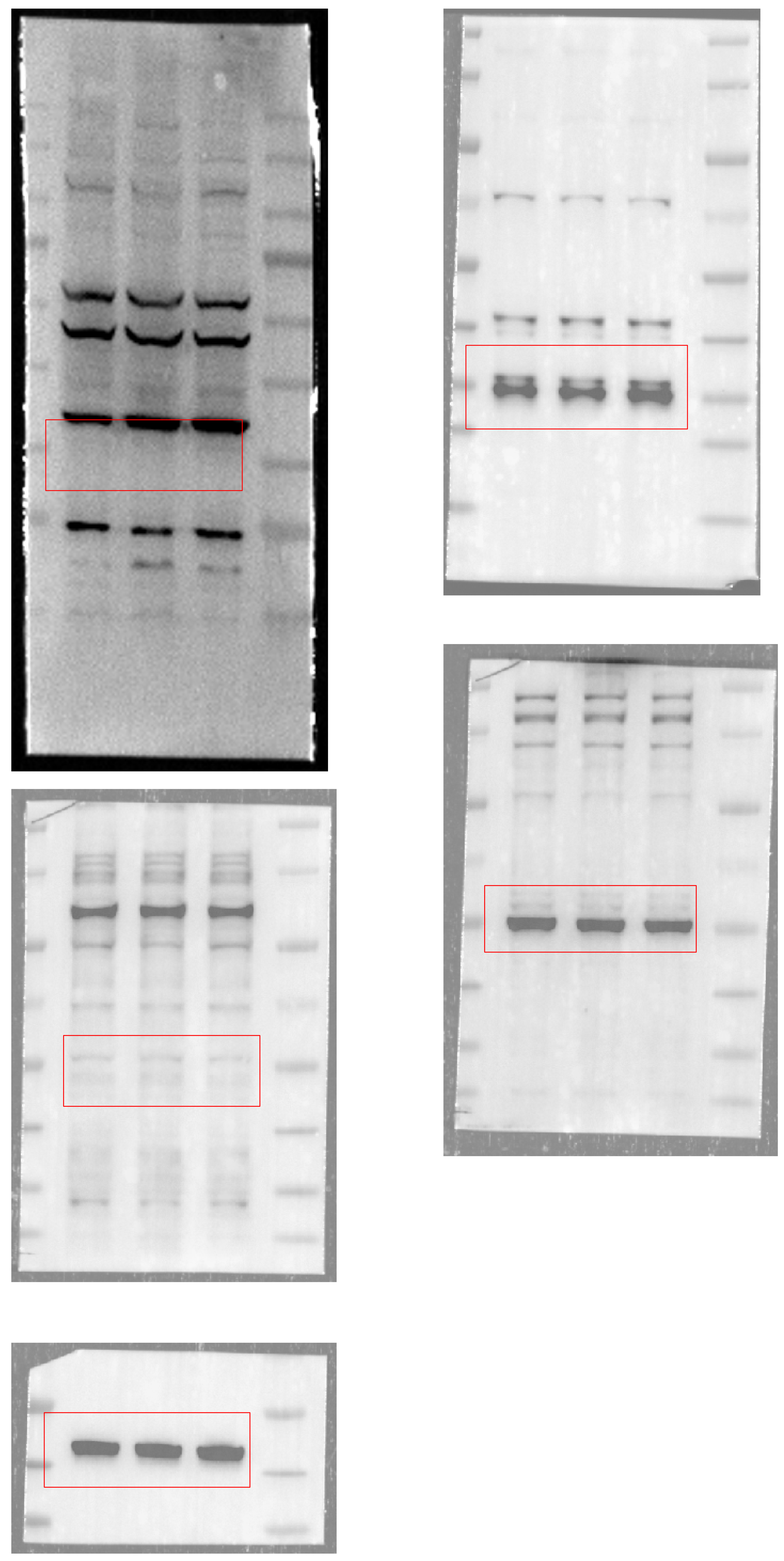

Supplement: Supplementary file 2 [file jci-136-203202-s063.pdf]
